# Anti-tumor effects of an ID antagonist with no observed acquired resistance

**DOI:** 10.1038/s41523-021-00266-0

**Published:** 2021-05-24

**Authors:** Paulina M. Wojnarowicz, Marta Garcia Escolano, Yun-Han Huang, Bina Desai, Yvette Chin, Riddhi Shah, Sijia Xu, Saurabh Yadav, Sergey Yaklichkin, Ouathek Ouerfelli, Rajesh Kumar Soni, John Philip, David C. Montrose, John H. Healey, Vinagolu K. Rajasekhar, William A. Garland, Jeremy Ratiu, Yuan Zhuang, Larry Norton, Neal Rosen, Ronald C. Hendrickson, Xi Kathy Zhou, Antonio Iavarone, Joan Massague, Andrew J. Dannenberg, Anna Lasorella, Robert Benezra

**Affiliations:** 1 Cancer Biology and Genetics Program, Memorial Sloan Kettering Cancer Center, New York, NY USA; 2 Weill Cornell/Sloan Kettering/Rockefeller Tri-Institutional MD-PhD Program, New York, NY 10065 USA; 3 Gerstner Sloan Kettering Graduate School of Biomedical Sciences, New York, NY 10065 USA; 4 Organic Synthesis Core Facility, Memorial Sloan Kettering Cancer Center, New York, NY USA; 5 Proteomics & Microchemistry Core Facility, Memorial Sloan Kettering Cancer Center, New York, NY USA; 6 Department of Medicine, Weill Cornell Medical College, New York, NY USA; 7 Orthopedics Service, Memorial Sloan Kettering Cancer Center, New York, NY USA; 8 Department of Medicine, Memorial Sloan Kettering Cancer Center, New York, NY USA; 9 Tosk, Inc., Mountain View, CA USA; 10 Department of Immunology, Duke University, Durham, NC USA; 11 Evelyn H. Lauder Breast Center, Memorial Sloan Kettering Cancer Center, New York, NY USA; 12 Molecular Pharmacology Program, Memorial Sloan Kettering Cancer Center, New York, NY USA; 13 Department of Healthcare Policy and Research Weill Cornell Medical College, New York, NY USA; 14 Department of Neurology, Department of Pathology, Institute for Cancer Genetics, Columbia University Medical Center, New York, NY USA; 15 Department of Pediatrics, Department of Pathology, Institute for Cancer Genetics, Columbia University Medical Center, New York, NY USA; 16 Present Address: Department of Pathology, Renaissance School of Medicine, Stony Brook University, Stony Brook, NY USA

**Keywords:** Cancer therapy, Metastasis

## Abstract

ID proteins are helix-loop-helix (HLH) transcriptional regulators frequently overexpressed in cancer. ID proteins inhibit basic-HLH transcription factors often blocking differentiation and sustaining proliferation. A small-molecule, AGX51, targets ID proteins for degradation and impairs ocular neovascularization in mouse models. Here we show that AGX51 treatment of cancer cell lines impairs cell growth and viability that results from an increase in reactive oxygen species (ROS) production upon ID degradation. In mouse models, AGX51 treatment suppresses breast cancer colonization in the lung, regresses the growth of paclitaxel-resistant breast tumors when combined with paclitaxel and reduces tumor burden in sporadic colorectal neoplasia. Furthermore, in cells and mice, we fail to observe acquired resistance to AGX51 likely the result of the inability to mutate the binding pocket without loss of ID function and efficient degradation of the ID proteins. Thus, AGX51 is a first-in-class compound that antagonizes ID proteins, shows strong anti-tumor effects and may be further developed for the management of multiple cancers.

## Introduction

The ID proteins, ID1, ID2, ID3, and ID4, are helix-loop-helix (HLH) transcriptional regulators. They function by binding to and sequestering basic-HLH (bHLH) transcription factors (e.g., the E protein E47). ID proteins are expressed during development and inhibit bHLH transcription factors to block differentiation and maintain self-renewal. ID protein expression is largely silenced in adult tissues but can be reactivated in several disease processes, including cancer^1–3^. Initial data associating ID1 and ID3 with cancer emerged from studies of xenografts and spontaneous tumors in genetically engineered mouse models, which typically showed decreased tumor growth and impaired angiogenesis in *Id1*- and/or *Id3*-deficient backgrounds^1,4,5^. ID1 and ID3 are highly expressed in virtually all human cancers, in the vasculature and/or the tumor cells, including solid tumors of the breast^6–8^, pancreas^9^, bladder^6^, cervix/uterus^10–12^, colon^13,14^, endometrium^15,16^, stomach^17,18^, nervous system^4,19,20^, liver^21^, ovary^22^, prostate^23–25^, esophagus^26^, lung^27–29^, and thyroid^30,31^ as well in hematopoietic tumors such as AML^32^. More recently, expression of ID1 and ID3 has been associated with anchorage-independent growth of invasive lobular carcinomas of the breast^33^. In virtually all of these cancer types the presence of ID1 and/or ID3 is associated with an aggressive phenotype and poor clinical outcome. However, mutations in the *ID* genes or their promoters are rarely found. One notable exception is Burkitt’s lymphoma where *ID3* is frequently mutated and appears to act as a tumor suppressor, perhaps due to the growth-promoting properties of E proteins in these cells^34^.

ID overexpression in cancer is largely due to the convergence of several pro-proliferative and pro-oncogenic signaling cascades on the ID promoters such as MAP kinase^35,36^, Myc^37^, BMPs^38,39^, Src^40^, FLT3^32^, VEGF^41^, Tgf-β^42^, Wnt^43^, and Notch signaling^44^. High ID expression is associated with increased metastatic potential. For example, in breast cancer, high levels of ID1, ID3, and ID4 are present in the tumor cells of the highly metastatic, triple-negative subtype^45,46^. In metastatic tumor cells, ID1 and ID3 facilitate the mesenchymal-to-epithelial conversion required for macro-metastatic disease progression, likely through antagonism of the bHLH protein Twist^47^.

We recently identified a small-molecule antagonist of ID proteins based on X-ray crystal structure coordinates and an in silico screen that targeted a hydrophobic crevice adjacent to the loop region of the HLH domain^48^. Approximately 350 molecules with drug-like properties were identified and tested for their ability to perturb ID1 interactions with target bHLH protein E47. The top candidate from this screen, AGX51, caused a change in the secondary structure of purified ID1 and inhibited the ability of ID1 to associate with its target E proteins in cells. This in turn led to the degradation of ID1 via ubiquitin-mediated proteolysis. All 4 members of the ID family were similarly destabilized by AGX51. Consistent with the low-level expression of ID proteins in normal adult tissues, high doses of the drug were well tolerated in mice with no apparent toxicity (see below). Here we report the characterization of AGX51 activity in several cancer models including cell lines, patient-derived xenografts (PDXs), pancreatic ductal adenocarcinoma (PDA) organoid cultures, xenograft and syngeneic mouse models, and a chemically induced murine model of colorectal adenoma. We demonstrate that ID inhibition by AGX51 impairs cell viability, increases reactive oxygen species (ROS) levels, and inhibits tumor growth and cancer cell colonization of the lungs in mice, thus phenocopying the effects seen in genetic animal studies. Importantly, long-term exposure of multiple cancer cell lines to AGX51 fails to demonstrate evidence of acquired resistance likely due to the binding of the drug to a region of the proteins that if mutated renders the protein non-functional.

## Results

### Effects of AGX51 on ID proteins

**Fig. 1:**
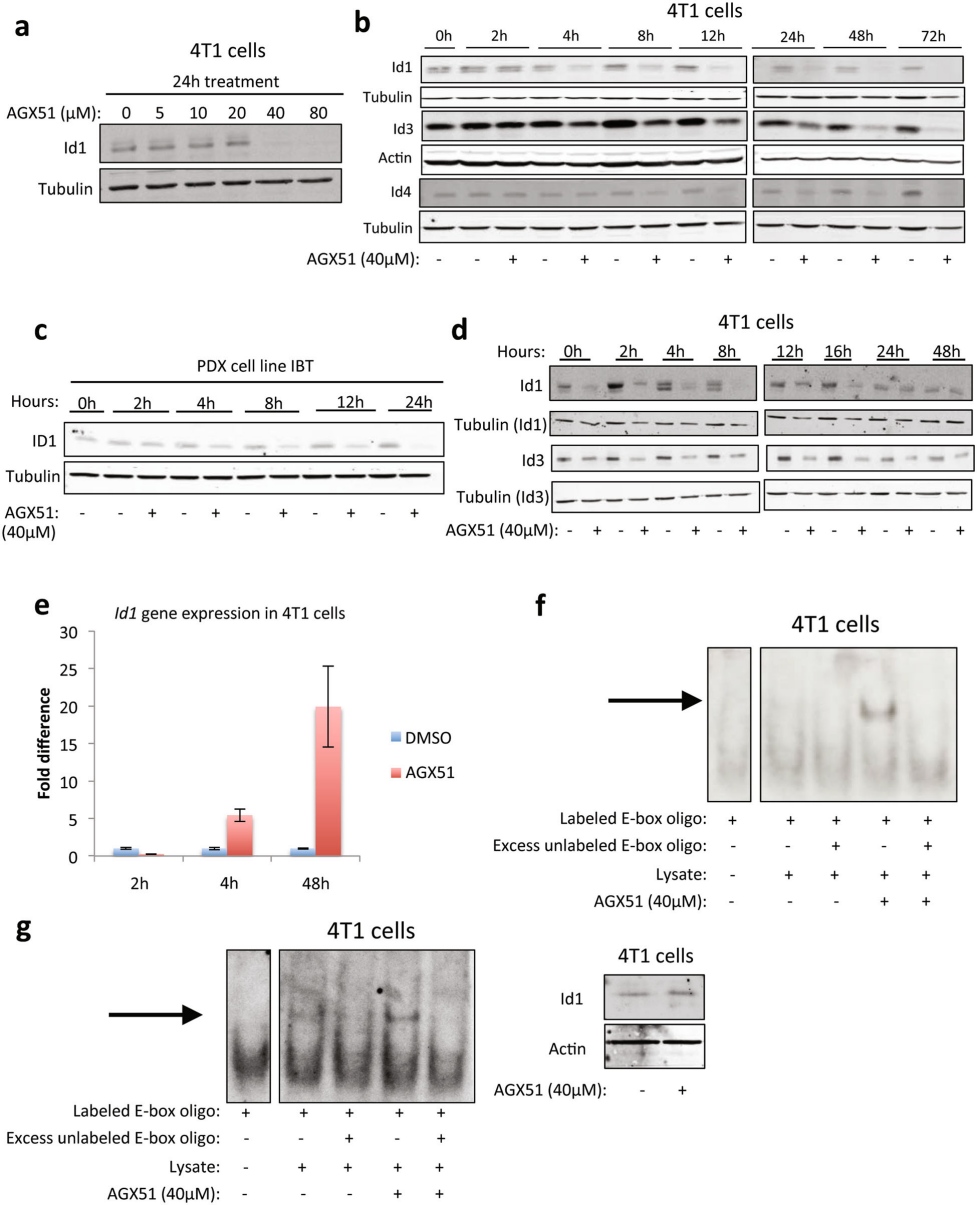
Effects of AGX51 on ID proteins. **a** Western blot for ID1 on whole-cell lysates from 4T1 cells treated with 0–80 µM AGX51 for 24 h. **b** Western blot for ID1, ID3, and ID4 on whole-cell lysates from 4T1 cells treated with 40 µM AGX51 for 0–72 h. Quantification of western blot bands is shown in Supplementary Fig. 1a. **c** Western blot for ID1 on whole-cell lysates from the PDX cell line IBT treated with 40 µM AGX51 for 0–24 h. **d** Western blot for ID1 and ID3 on whole-cell lysates from 4T1 cells treated with 40 µM AGX51 for 24 h, after which compound was washed off and cells were collected for 0–48 h post compound removal. **e** qRT-PCR analysis for *Id1* in 4T1 cells treated with 40 µM AGX51 for 2, 4, or 48 h. Mean fold difference with error bars showing standard error of the mean (SEM) of technical triplicates. The experiment was performed at least three times. **f** Electrophoretic mobility shift assay (EMSA) on whole-cell lysates from 4T1 cells treated with 40 µM AGX51 for 24 h. Arrow indicates binding to DNA. **g** EMSA on whole-cell lysates from 4T1 cells treated with 40 µM AGX51 for 1 h, with corresponding western blot for *Id1* shown to the right of the EMSA blot. Arrow indicates binding to DNA. Tubulin/actin are used as protein loading controls. See also Supplementary Fig. 1.

We recently reported that AGX51 treatment of HUVEC and HCT116 cells leads to disruption of the ID–E protein–protein interaction (PPI), resulting in ubiquitination of ID1 and its subsequent degradation^48^. To further characterize the effects of AGX51 in cancer cells, we treated the 4T1 murine mammary cancer cell line with increasing concentrations of AGX51 (0–80 μM) for 24 h and observed a significant decrease in ID1 protein levels starting at 40 μM (Fig. 1a). 4T1 cells treated with 40 μM AGX51 for 0–72 h showed a decrease in ID1 levels starting at 4 h, with near-complete ID1 loss by 24 h (Fig. 1b and Supplementary Fig. 1a). ID3 and ID4 are also lost albeit with delayed kinetics. ID2 is not expressed in 4T1 cells (data not shown) but we previously showed that ID2 levels decrease in response to AGX51 treatment in HCT116 cells^48^. Similar to 4T1 cells, HMLE *RAS Twist* cells (RAS transformed human mammary epithelial cells overexpressing Twist) also showed reduced levels of ID1, ID3, and ID4 when treated with 20 μM AGX51 over similar time courses (Supplementary Fig. 1b). A decrease in ID1 and ID3 protein levels was also observed in a PDX cell line (IBT) generated from a patient with triple-negative breast cancer (TNBC) (Fig. 1c and Supplementary Fig. 1c). The PDX cell line does not express ID2 and ID4 (data not shown). To determine the duration of ID1 and ID3 protein loss, 4T1 cells were treated with 40 μM AGX51 for 24 h, then cultured in AGX51-free media over 48 h. ID1 protein levels increased over time, reaching untreated levels by 24 h (Fig. 1d). ID3 protein levels showed slower recovery (Fig. 1d).

To determine if the reduction in ID protein levels was secondary to reduced transcription, we measured *Id1* mRNA levels from 4T1 cells treated with AGX51 over a 48 h period. By qRT-PCR, we observed a modest increase of *Id1* mRNA at early time points, with a dramatic increase in expression by 48 h (5x and 20x, respectively, Fig. 1e). A similar result was observed for *Id3* (data not shown). The increase in *Id1 and Id3* mRNA levels suggests that the cell is attempting to compensate for ID1 and ID3 protein loss, likely through the ability of the liberated E proteins to transactivate the *Id* promoters analogous to what is seen in *Drosophila melanogaster*^49^ where daughterless (an E protein orthologue) activates *extramachrochaete* (an *Id* orthologue). To address this possibility we obtained T cells from E2A/HEB double knockout mice^50,51^ and treated them ex vivo with AGX51. These cells express ID3 and ID2 but not ID1. Compared to wild-type T cells, E protein knockout T cells show a reduction in *Id3* mRNA levels following treatment with AGX51 (Supplementary Fig. 1d). A similar trend is seen with *Id2* mRNA. These findings support the hypothesis that liberation of E proteins contributes to the increase in *Id1* mRNA observed after AGX51 treatment of our cancer cells.

We next tested whether loss of ID proteins in response to AGX51 resulted in increased E protein–DNA binding. As expected, treating 4T1 cells with AGX51 for 24 h resulted in an increase in E protein binding (Fig. 1f). Similar electrophoretic mobility shift assay (EMSA) results were obtained using cell lysates from HMLE *RAS Twist* cells, and the PDX cell line (Supplementary Fig. 1e). To determine if the loss of ID activity precedes ID protein loss, EMSAs were carried out after 1 h of AGX51 treatment. An increase in E protein binding occurred at a time when no ID1 protein loss was observed (Fig. 1g), suggesting that the increase in E protein binding is due to disruption of the ID1–E PPI as opposed to decreased overall ID levels. This conclusion is supported by direct evidence of the disruption of the ID1–E47 interaction observed in HCT116 cells^48^.

### Effects of AGX51 on cell growth

**Fig. 2:**
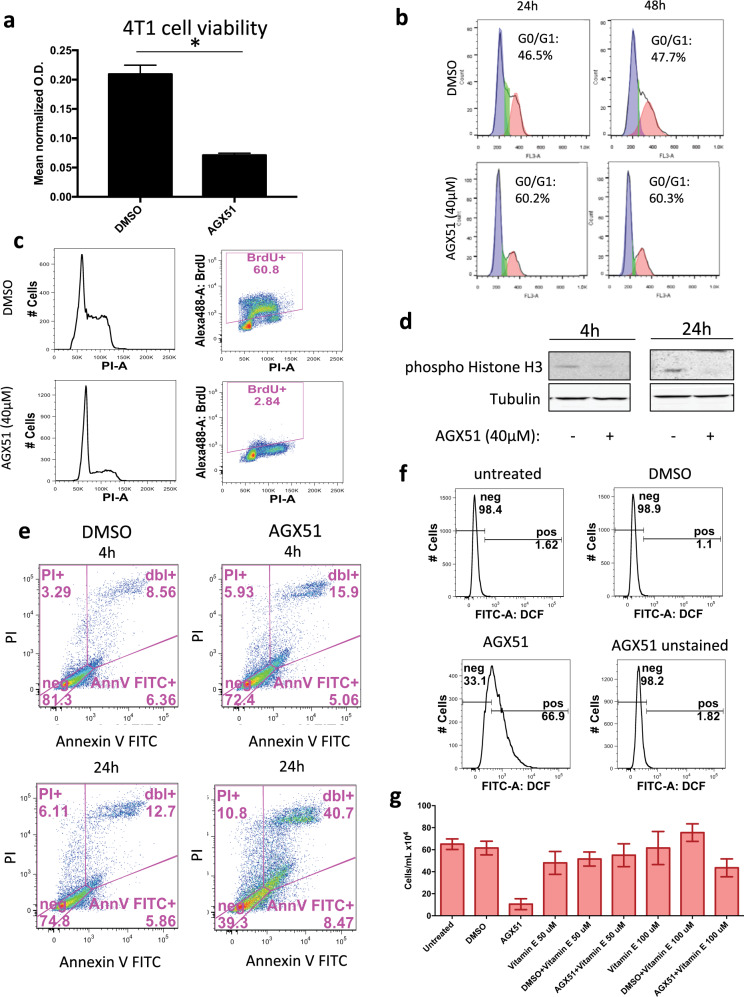
Effects of AGX51 on cells in culture. **a** Alamar blue cell viability assay on 4T1 cells treated with 40 µM AGX51 for 24 h. Mean value of technical triplicates is plotted with error bars representing SEM. * Indicates *p* < 0.001, as determined by unpaired *t*-test. The experiment was repeated at least three times. **b** Cell cycle analysis on 4T1 cells treated with 40 µM AGX51 for 24 and 48 h; the experiment was repeated three times. **c** BrdU incorporation by 4T1 cells treated with 40 µM AGX51 or vehicle, for 24 h as measured by flow cytometry. **d** Western blot for phospho-histone H3 on lysates from 4T1 cells treated with 40 µM AGX51 or DMSO for 4 and 24 h. **e** Annexin V and PI staining of 4T1 cells treated with AGX51 for 4 and 24 h, as analyzed by flow cytometry; the experiment was repeated two times. **f** Reactive oxygen species accumulation in 4T1 cells induced by AGX51 treatment, as determined by H2DCFDA staining followed by flow cytometry analysis. AGX51-treated cells not stained with H2DCFDA were analyzed as an auto-fluorescence control. The experiment was repeated three times. **g** Cell viability, as measured by trypan blue exclusion and cell counting of 4T1 cells treated with 40 µM AGX51 or DMSO for 24 h, with or without 1 h pretreatment with vitamin E (50 or 100 µM). Means are plotted with error bars showing standard deviation (SD) of four technical replicates. See also Supplementary Fig. 2.

To characterize the effects of AGX51-mediated ID protein loss on cell growth, we determined the IC_50_ of AGX51 in 4T1 cells and nine other breast cancer cell lines representing the major breast cancer subtypes (ER+, HER2+, and TNBC), and three breast cancer PDX cell lines (Supplementary Table 1 and Supplementary Fig. 1f, g). Due to the observed viability curves of certain cell lines, the IC50 had to be extrapolated to fit the curve (these approximate values are indicated by asterisk in Supplementary Table 1). Of the different breast cancer subtypes, the TNBC cell lines were most sensitive (~25 μM) (Supplementary Table 1). The IC_50_ of 4T1 cells, a model of TNBC {Aslakson, 1992 #5667; Pulaski, 2001 #5668} was similar to that of the TNBC cell lines (Supplementary Table 1). The effect of AGX51 on 4T1 cells was also determined using Alamar blue viability assays, which show a significant reduction (~3-fold, *p* = 0.0009) in viability after 24 h of treatment with 40 μM AGX51 (Fig. 2a).

It has previously been reported that genetic loss of *Id1* induces a G0/G1 growth arrest in mouse and human fibroblasts {Barone, 1994 #315; Hara, 1994 #314}. After 24 and 48 h of AGX51 treatment, 4T1 cells showed an accumulation in G0/G1 (Fig. 2b). AGX51 treatment also led to a reduction in BrdU incorporation (Fig. 2c) and phospho-histone H3 levels (Fig. 2d). After 24 h of AGX51 treatment, we saw an increase in the Annexin V+/PI+ fraction (12.7% vs. 40.7%), indicative of late apoptosis and necrosis (Fig. 2e). We observed no overt increase in cleaved caspase 3 or cleaved PARP levels, suggesting that the mechanism of cell death is largely non-apoptotic (data not shown).

A feature of necrotic cell death is the production of ROS. To determine the effect of AGX51 on ROS production in 4T1 cells, we treated cells with 40 μM AGX51 for 24 h, stained with 2ʹ,7ʹ-dichlorodihydrofluorescein diacetate (H2DCFDA) and analyzed the cells by flow cytometry. We observed a significant increase in the fluorescein-positive fraction upon AGX51 treatment, indicative of an increase in ROS production (Fig. 2f). In fact, AGX51 increased ROS production to a greater degree than our positive control, H_2_O_2_ (Supplementary Fig. 2a). Similar effects were seen in HCT116 cells (Supplementary Fig. 2b). Furthermore, addition of 50 and 100 μM of the antioxidant vitamin E rescued cell viability, and reduced ROS levels in both cell lines (Fig. 2g and Supplementary Fig. 2c–g). Vitamin E treatment also rescued the AGX51-mediated cell cycle arrest in HCT116 cells (Supplementary Fig. 2h) and 4T1 cells (data not shown), suggesting that the cell cycle arrest is secondary to ROS production. ROS leading to cell cycle arrest has been previously reported^52,53^. Interestingly, other free radical scavengers such as N-acetyl cysteine (NAC), MnTBAP, MitoQ, MitoTEMPO, Apocynin, and Catalase did not rescue the AGX51-induced phenotypes (Supplementary Fig. 3). This observation raises the possibility that an oxidized lipid may be a key mediator of AGX51-mediated killing activity since vitamin E is an efficient inhibitor of lipid peroxidation^54^.

Other potential non-apoptotic mechanisms of cell death such as ferroptosis and necroptosis may be at play in mediating AGX51-induced reduced cell viability. To investigate these possibilities we co-treated 4T1 cells with AGX51 and ferrostatin-1 or AGX with necrostatin-1. Neither ferrostatin-1 nor necrostatn-1 were able to rescue the decreases in cell viability seen with AGX51 treatment (Supplementary Fig. 2i, j), suggesting that these cell death mechanisms are not involved in AGX51-mediated effects. Nonetheless, our findings are consistent with a recent report where siRNA-mediated ID1 and ID3 inhibition was associated with increased ROS levels^55^ and indicate that a main mechanism of AGX51-mediated cell killing is ROS-mediated. We also suggest that the G0/G1 arrest we see prior to cell death may in fact be ROS-mediated as has been observed in other systems^52,53^.

**Fig. 3:**
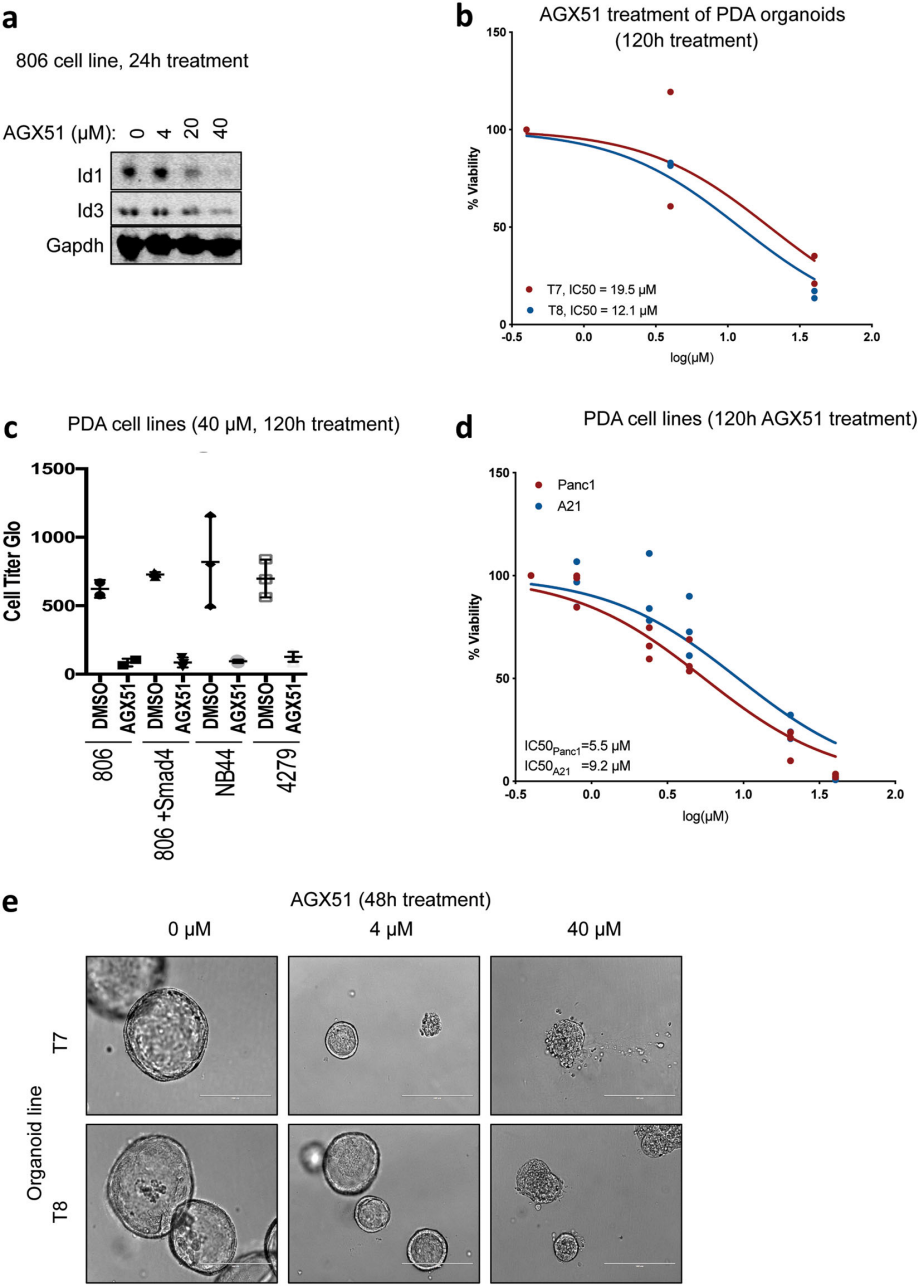
Effects of AGX51 on pancreatic ductal adenocarcinoma cells. **a** Western blot for ID1 and ID3 on whole-cell lysates from 806 pancreatic ductal adenocarcinoma (PDA) cells treated with 4, 20, and 40 µM AGX51 for 24 h. GAPDH used as loading control. **b** Dose–response of PDA organoid lines T7 and T8 to AGX51 following 120 h of treatment, with IC_50_ indicated. **c** Cell viability as assessed by Cell Titer Glo assay for PDA lines 806, 806 + Smad4, NB44, and 4279 treated with 40 µM AGX51 or DMSO vehicle control for 120 h (three replicates, error bars, standard deviations, SD). **d** Dose–response of PDA cell lines Panc1 and A21 to 120 h of AGX51 treatment, with IC_50_ indicated. **e** Bright field images of T7 and T8 PDA organoid lines treated with 4 and 40 µM AGX51 for 48 h. Scale bars represent 200 µm.

Enforcement of ID1 expression has previously been reported to play an important role in circumventing Tgf-β’s tumor-suppressive effect in pancreatic cancer. Depletion of the ID proteins by shRNAs has been shown to decrease the ability of pancreatic cancer cells to survive and form orthotopic tumors^56^. We tested whether AGX51 would have similar effects on pancreatic cancer cells. In line with the results obtained in other cancer models that we tested, AGX51 caused a depletion of the ID1 and ID3 proteins in the pancreatic cancer cell line 806, starting at a dose between 4 and 20 µM (Fig. 3a) with ID3 showing slightly less sensitivity as observed in other cellular and biophysical assays^48^. Furthermore, AGX51 decreased cell viability in a variety of pancreatic cancer cell and organoid lines, including mouse organoids (Fig. 3b), mouse cell lines 806, NB44 and 4279 (Fig. 3c), and human cell lines Panc1 and A21 (Fig. 3d) with an IC_50_ of 5.5–19.5 µM. This corresponds to the range of concentrations of AGX51 required for ID protein loss. Of note, when treated with 40 µM of AGX51, well-formed organoid structures collapsed (Fig. 3e).

### Specificity of AGX51 activity

**Fig. 4:**
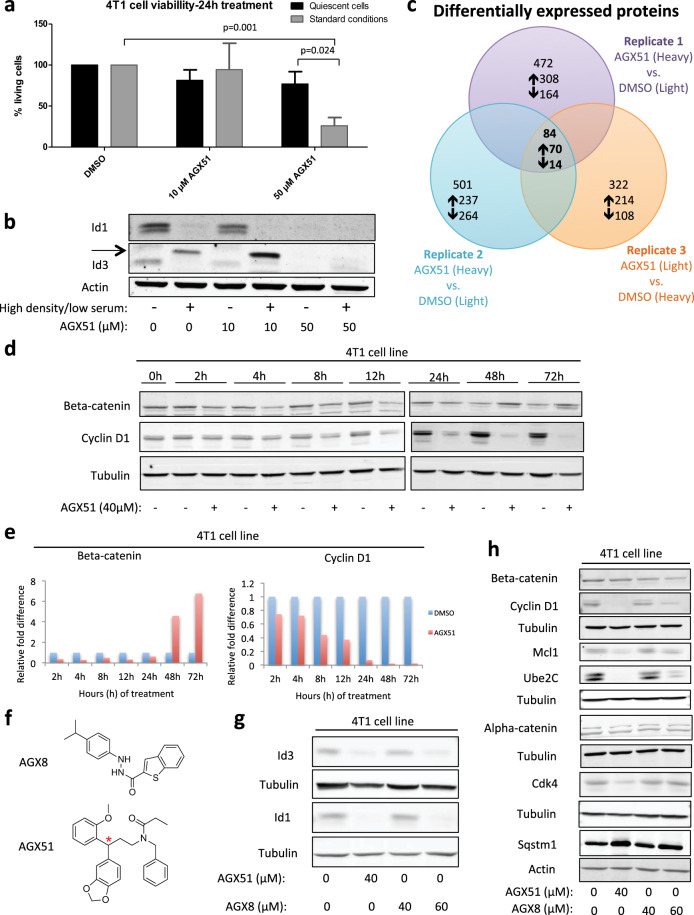
Assessing the effect of AGX51 on quiescent cells and the whole proteome. **a** 4T1 cells were treated with AGX51 (10 or 50 µM), when they were cultured under standard conditions (standard density, standard serum (10%)) or under quiescence-inducing conditions (high density, low serum (1%)). Following 24 h of AGX51 treatment, cell numbers were determined by cell counting and trypan blue exclusion. Means are plotted with error bars showing SD of four technical replicates. **b** Western blot for ID1 and ID3 on whole-cell lysates from 4T1 cells presented in (**a**). Actin serves as the protein loading control. Arrow indicates non-specific band. **c** Venn diagram of differentially expressed proteins, as identified by mass spectrometry, from three replicates of SILAC-4T1 cells treated with 40 µM AGX51 for 4 h. **d** Western blot for Beta-catenin and Cyclin D1 on whole-cell lysates from 4T1 cells treated with 40 µM AGX51 for 0–72 h. **e** Quantification of western blot shown in (**d**). **f** Structures of AGX8 and AGX51 (red asterisk indicating chiral center). **g** Western blot for ID3 and ID1 on whole-cell lysates from 4T1 cells treated with AGX51 and AGX8 at the indicated concentrations. **h** Western blot for the indicated proteins on whole-cell lysates from 4T1 cells treated with AGX51 and AGX8 at the indicated concentrations. Quantification of western blot bands shown in (**g**) and (**h**) are in Supplementary Fig. 7. See also Supplementary Figs. 4–7.

A common concern with any novel small-molecule is specificity. Our results showing loss of ID proteins, an increase in liberated E proteins, and cell phenotypes consistent with genetic models of ID loss indicate that AGX51 is targeting ID proteins. However, AGX51 may also be affecting other, unintended targets. If ID proteins are the primary targets of AGX51 then we reasoned that reducing ID1 or ID3 levels by shRNA should increase the sensitivity of the cells to AGX51 by reducing the concentration of the target. A similar approach was used to show that reduction of Ras genes sensitized cells to a pan-Ras inhibitor^57^. Similarly, cells able to survive in the absence of ID proteins, such as quiescent cells should be resistant to AGX51. Upon partial knockdown of *Id1* or *Id3* by shRNAs (Supplementary Fig. 4a) the cells showed increased sensitivity to AGX51 (~3-fold) (Supplementary Table 1). 4T1 cells allowed to reach confluence under low-serum conditions become quiescent and display near-complete loss of ID expression (Fig. 4a, b). Treating these cells with AGX51 led to a dramatic reduction in cell killing relative to cycling control cells. Together these data support the conclusion that the ID proteins are the primary targets in cycling cells.

Having established that AGX51 disrupted PPI leading to proteasome-dependent degradation of ID proteins^48^, we wondered if levels of other proteins would be affected. To determine the full range of consequences of AGX51 exposure, we performed Stable Isotope Labeling of Amino acids in Cell culture (SILAC) on 4T1 cells treated with AGX51 or DMSO for 4 h (at which point we would expect to see a decrease in ID1) and profiled the whole proteome by mass spectrometry. We identified 6869 proteins/protein clusters with an FDR < 1%, where 2735 proteins were found in all three replicate experiments. As expected, ID1 was underexpressed in all three replicates (fold differences: −1.39, −1.71, and −2.07). Using a 1.35-fold cutoff, we identified 13 other underexpressed and 70 overexpressed proteins (Fig. 4c and Supplementary Table 2). ID2 and ID4 were not detected in this analysis and ID3 showed no significant change at 4 h, consistent with the data shown above (see Fig. 1b). The underexpressed proteins included Beta-catenin, Cyclin D1, Cdk4, and Ube2C, which are all involved in cell cycle progression and were validated by western blot (Fig. 4d, e and Supplementary Fig. 4b). The underexpression of these proteins at early time points is modest by SILAC and hence quantitative differences by western blot do not become prominent until later time points, and in some cases are transient (Beta-catenin). An additional three underexpressed proteins were also validated by western blot (Mcl1, Sqstm1, and Alpha-catenin) (Supplementary Fig. 4b) although the Sqstm1 inhibition was also transient. Sqstm1 encodes the protein Sequestosome-1 which targets other proteins to which it binds for degradation by autophagy^58^. These changes were also observed in the PDX cell line and HMLE *RAS Twist* cells (Supplementary Fig. 5). We saw no change in *Beta-catenin* mRNA levels for 24 h of AGX51 treatment of 4T1 cells, and only saw a significant decrease in *Cyclin D1* message at later time points (~1-fold at 24 h and ~4-fold at 48 h), suggesting that at early time points the changes in Beta-catenin and Cyclin D1 proteins may be occurring post-transcriptionally (Supplementary Fig. 6a). We tested the gene expression of *Cdk4* and *Mcl1*, and also saw no significant decrease in mRNA over time with AGX51 treatment and saw upregulation at later time points (Supplementary Fig. 6b). Importantly, the loss of these proteins takes place prior to the changes in cell cycle distribution (Supplementary Fig. 6c), arguing against the possibility that these protein changes are secondary to a G0/G1 arrest.

Genetic suppression of *Id1–4* could be used to test the possibility that the proteome changes seen after AGX51 treatment are secondary to ID protein loss but a timed, simultaneous reduction of all ID proteins prior to cell cycle arrest has been technically impossible. While overexpression of ID proteins would not necessarily yield the converse result, we nonetheless attempted to assess the proteome changes that occurred in ID1- and ID3-overexpressing 4T1 cells. This approach was also confounding since we found that ID3 overexpression reduced endogenous ID1 levels and that ID1 overexpression reduced ID3 levels so that less-than-expected changes in total ID1/3 levels were observed. Despite this we did see minor increases in Cyclin D1 levels in the overexpressing cell lines as expected (Supplementary Fig. 6d) but many of the other genes were unaffected (data not shown).

Another approach was to examine the effects of a structurally unrelated compound (AGX8) that also scored positive in the initial EMSA screen for perturbation of the ID1–E47 interaction^48^. AGX51 and AGX8 contain 2 distinct chemical scaffolds with different interaction patterns. While AGX51 has only hydrogen acceptor groups, AGX8 has 2 hydrogen donors and 2 hydrogen acceptor groups. Treating 4T1 cells with AGX8 for 24 h also led to reduced levels of ID1 and ID3 although the concentration needed to see this decrease was greater than that for AGX51 (60 vs. 40 μM) (Fig. 4f, g and Supplementary Fig. 7a). In addition, the cell viability effects of AGX8 were less pronounced than those of AGX51 (Supplementary Table 3). Western blot analyses found that the levels of Cyclin D1, Beta-catenin, Ube2C, and Mcl1 decreased when 4T1 cells were treated with 60 μM AGX8, similar to 40 μM of AGX51 treatment. Very modest decreases were observed for Cdk4 and Alpha-catenin with 40 μM AGX51 but were difficult to discern with 60 μM AGX8 (Fig. 4h and Supplementary Fig. 7b). In addition, there was an increase in Sqstm1 expression after 24 h of AGX8 treatment, similar to that seen with AGX51 treatment (Fig. 4h and Supplementary Fig. 7b). The similarity in the effects of AGX51 and AGX8 on many of the proteins identified by SILAC is consistent with the idea that these changes are secondary to ID loss.

### Effects of AGX51 on tumor growth and lung colonization of cancer cells

**Fig. 5:**
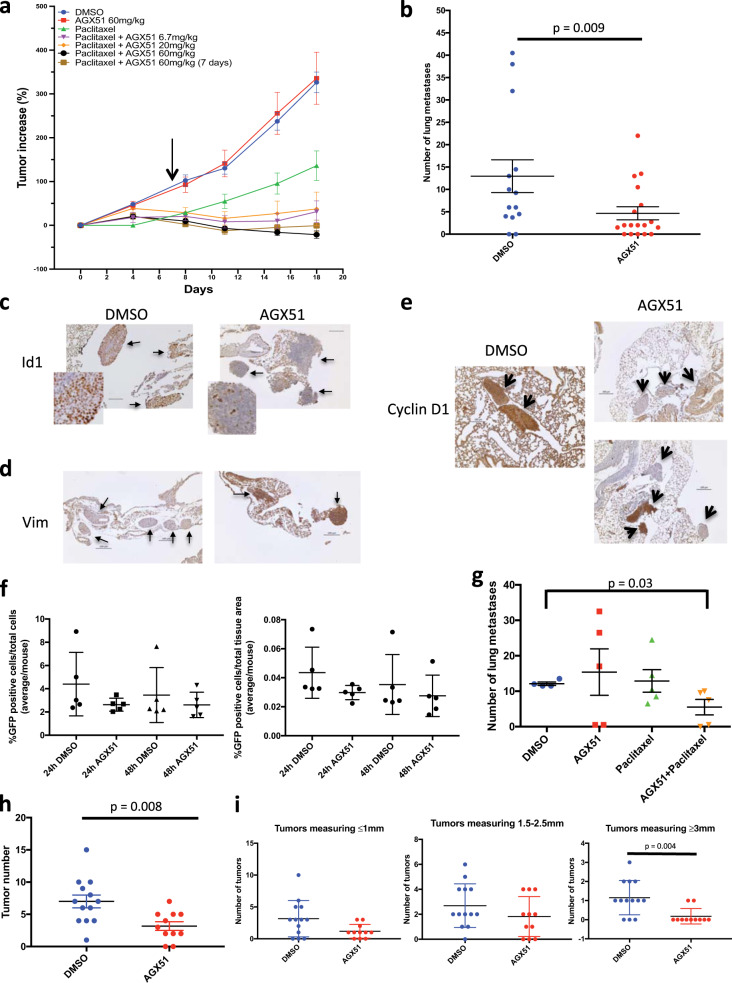
Effects of AGX51 on tumor growth and metastasis. **a** Mean tumor volume increase in athymic nu/nu mice implanted with MDA-MB-231 cells in the mammary fat pad and treated with AGX51, paclitaxel, AGX51 + paclitaxel, or vehicle control (*n* = 5 mice per treatment group for all groups except paclitaxel alone where *n* = 4 due to 1 death). Compared to controls, the effect of all AGX51 + paclitaxel treatments was significant by weighted least squares (*p* ≤ 0.001) and AGX51 + paclitaxel treatments were also significantly different from AGX51 treatment alone (*p* ≤ 0.001). The mean is plotted with error bars representing the SEM. **b** Average number of lung metastases in Balb/c mice injected with 5 × 10^4^ 4T1 cells via tail vein, treated with 50 mg/kg AGX51 bid or vehicle (*n* = 18 or *n* = 14, respectively). Means are plotted with error bars showing SEM. AGX51 treatment had a significant effect on lung tumor development (*p* < 0.009 by weighted least squares). **c** Examples of ID1 IHC staining in lung sections from mice described in (**b**). **d** Examples of Vimentin IHC staining in lung sections from mice described in (**b**). **e** Examples of Cyclin D1 IHC staining in lung sections from mice described in (**b**), with examples of metastases from AGX51-treated mice showing negative (upper panel) and positive (lower panel) staining for Cyclin D1. Bars are 100 µm in length. Staining in (**c**–**e**) was performed on the lesions of 5 animals in each group and representative images are shown. **f** Quantification of the number of GFP-labeled 4T1 cells in the lungs of Balb/c mice injected with 5 × 10^4^ 4T1 cells via tail vein and treated with 50 mg/kg bid or vehicle for 24 and 48 h (*n* = 5 mice per treatment group). Means are plotted with error bars showing SD. **g** Average number of lung metastases in Balb/c mice injected with 5 × 10^4^ 4T1 cells via tail vein and treated with AGX51, paclitaxel, AGX51 + paclitaxel, or vehicle once lung metastases were established (*n* = 5 mice per treatment group). Means are plotted with error bars showing SEM. Compared to vehicle, AGX51 + paclitaxel treatment had a significant effect on lung tumor growth (*p* < 0.03 by weighted linear regression). **h** Number of colon tumors in AOM colon tumor model in A/J mice that were treated with DMSO (*n* = 13) or AGX51. Means are plotted with error bars showing SEM. AGX51 treatment resulted in a significant reduction in the number of tumors (*p* = 0.008). **i** Size distribution of tumors in (**h**), grouped by treatment group (AGX51 or DMSO), where AGX51 treatment resulted in a significant reduction in tumors measuring ≥3 mm (*p* = 0.004). Mean are plotted with error bars showing SD. Colon tumor count data were analyzed using the Wilcoxon rank sum test. For additional data pertaining to the effects of AGX51 on tumor growth and metastases see Supplementary Fig. 9.

In spontaneous primary tumors of the breast in the MMTV-Her2/neu model, genetic analyses indicated that loss of *Id1* and/or *Id3* was insufficient to inhibit tumor growth and needed to be combined with suboptimal doses of chemotherapy to cause regression^59^. We tested AGX51 in a primary tumor xenograft model using MDA-MB-231 cells with and without paclitaxel. Nude mice were implanted with MDA-MB-231 cells and tumors were allowed to grow to ~100 mm^3^ before the initiation of the following treatments: vehicle (DMSO); paclitaxel 15 mg/kg qd (quaque die, i.e., once a day) on days 1–5; AGX51 60 mg/kg bid (bis in die, i.e., twice a day) for all 19 days; or paclitaxel 15 mg/kg on days 1–5 plus AGX51 6.7 mg/kg, 20 mg/kg, or 60 mg/kg bid for all 19 days. Also included was a treatment group that received 15 mg/kg paclitaxel qd on days 1–5 and 60 mg/kg of AGX51 for just 7 days. Paclitaxel treatment alone had a modest inhibitory effect on tumor growth while the addition of 6.7 and 20 mg/kg AGX51 to paclitaxel almost completely inhibited tumor growth and the 60 mg/kg dose resulted in tumor regression (Fig. 5a and Supplementary Table 4). Interestingly, just 7 days of AGX51 treatment, in combination with paclitaxel, was sufficient to cause tumor regression, suggesting a long-lived anti-tumor effect that may persist even after treatment has stopped (Fig. 5a and Supplementary Table 4). AGX51 treatment alone did not suppress tumor growth. Thus, similar to the previously conducted genetic loss-of-function experiment^59^, AGX51 treatment leads to synergistic growth inhibition of primary breast tumors when combined with chemotherapy. This is not due to synergistic killing at the cellular level since we observed no synergistic effect on cell viability when MDA-MB-231 cells in culture are treated with AGX51 and paclitaxel (Supplementary Fig. 8a). While such synergy in vivo may be due to effects of ID loss on neoangiogenesis, in the current in vivo study we observed no obvious changes in angiogenesis or vascularization by gross observation of the tumors, histological evaluation, or CD31 staining by IHC (data not shown, see also ‘Discussion’).

Of note, we have previously shown that after 14 days of daily treatment at the highest dose used in any of our studies (60 mg/kg bid), there were no statistically significant differences between the AGX51-treated animals and the vehicle-treated animals with respect to weight, clinical chemistry and hematology plasma values, and histopathological evaluation of major organs at necropsy^48^. Similarly, when combining AGX51 bid with 15 mg/kg paclitaxel (qd for the first 5 days of a 14-day regimen), a regimen used in the xenograft studies described above, no overt toxicities were observed using any of the above-listed parameters (Supplementary Fig. 8b). Thus, in addition to showing anti-tumor efficacy, AGX51 is well tolerated in mice in this concentration range, alone and in combination with paclitaxel.

Genetic loss of *Id1/Id3* prevents the development of breast cancer lung metastasis by blocking the conversion of micro-metastases to macro-metastases^47^ and by blocking angiogenesis^4,60^. To determine whether AGX51 phenocopies these results, we used lung colonization assays, a model of experimental metastasis, where luciferase-labeled 4T1 cells, known to be metastatic, were injected into the tail veins of Balb/c mice and after 24 h the mice were treated with 50 mg/kg of AGX51 i.p. bid or vehicle. Colonization of the lungs by 4T1 cells and their growth into tumors (referred to as metastases) was monitored weekly by in vivo bioluminescent imaging. The experiment was stopped at 4 weeks following tail vein injection, due to the deteriorating condition of the control group. Bioluminescent imaging showed significant inhibition of lung metastasis development in the AGX51 group, and this correlated with the metastatic burden observed upon histopathological examination of lung tissues (*p* = 0.009) (Fig. 5b and Supplementary Fig. 9a). Treatment with AGX51 50 mg/kg once daily also decreased the number of lung metastases, and while still significant (*p* = 0.0023) the effect was not as dramatic as that seen with the bid regimen (Supplementary Fig. 9b, c). While all macro-metastases showed strong positive IHC staining for ID1, in smaller lung metastases the ID1 staining was decreased in the AGX51 group, relative to metastases of a similar size in the DMSO group (Fig. 5c). Conversely, as predicted from the genetic analyses^47^, Vimentin staining of small metastatic tumors in the AGX51 group was significantly higher than that in the DMSO group (Fig. 5d), consistent with a reduction in *Id1/Id3* levels blocking the mesenchymal-to-epithelial transition during metastatic progression. Interestingly, Vimentin was one of the proteins found overexpressed in our SILAC analysis (see Supplementary Table 2).

To characterize the effects of AGX51 on the expression of EMT-related proteins we assessed Vimentin, E-cadherin, Snail, Twist, and Zeb1 levels in AGX51-treated 4T1 cells. After an 8 h exposure, we observed a decrease in E-cadherin and an increase in Vimentin but by 48 h both proteins were increased. Decreases in Snail and Twist1 were observed throughout the time course, while Zeb1 levels were unchanged (Supplementary Fig. 10). Thus AGX51 modulates the levels of some EMT-related proteins, however, the precise mechanisms leading to these changes and their consequences remain to be elucidated. The changes observed do not appear to adhere to canonical EMT patterns, which may be evidence of AGX51 inducing partial EMT/MET likely secondary to ID protein loss^47^. The occurrence of partial or intermediate EMT phenotypes in cancer is becoming increasingly recognized^61–63^. Finally, consistent with the reduced Cyclin D1 levels shown above, IHC staining for Cyclin D1 in the 4T1 lung metastases of the AGX51 group was significantly reduced relative to the DMSO group (*p* < 0.00001) (Fig. 5e). While all DMSO group metastases stained positive for Cyclin D1, about half of the AGX51 group was negative (Fig. 5e upper panel of AGX51 group).

To determine whether AGX51 inhibits the extravasation and initial seeding at the secondary site or the progression of extravasated cancer cell outgrowth into tumors, we tail vein-injected the mice with GFP-labeled 4T1 cells and treated with AGX51 (50 mg/kg) or DMSO for 24 or 48 h then stained the lungs for GFP (i.e., tumor cells) and quantified the tumor cell number. There was no significant difference in the number of GFP-positive cells between AGX51 and DMSO groups after either 24 or 48 h of treatment (Fig. 5f). These data are consistent with genetic models that found that *Id1/Id3* inhibition does not prevent extravasation of metastasizing breast cancer cells, but rather the conversion of micro- to macro-metastatic lesions^45,47^.

To ascertain whether AGX51 is capable of inhibiting the growth of established lung tumors, we tail vein-injected the mice with 4T1 cells, as described above, and started treatment once lung signal was visible by bioluminescent imaging. Mice were then divided into 4 groups and treated with AGX51, paclitaxel, AGX51 + paclitaxel, or vehicle. Subsequent bioluminescent imaging and histopathological examination of the lungs found that while AGX51 or paclitaxel alone did not have an effect on the lung tumor growth, combination treatment significantly reduced the continued expansion of lung tumors relative to the other groups (*p* = 0.03) (Fig. 5g). In addition to suppressing the number of lung tumors, the tumors in the treated group were smaller in size (data not show). Thus, consistent with the primary tumor data, established lung tumors can be more effectively treated when AGX51 is combined with paclitaxel.

To determine the effects of AGX51 in an autochronous tumor model, we turned to the azoxymethane (AOM) colon tumor model, a chemically induced autochthonous model of adenoma. Mice were administered AOM once a week for 6 weeks, followed by a 3-week treatment break and then treated with AGX51 (15 mg/kg) or DMSO bid for 3 weeks. AGX51 treatment resulted in a significant decrease in the number of colon tumors (*p* = 0.008). In addition, the tumors from AGX51 group were smaller than those in the DMSO group, and this difference was significant when tumors measuring >3 mm were compared (*p* = 0.004) (Fig. 5h, i). Thus AGX51 shows anti-tumor activity in an autochronous cancer setting.

### Investigating mechanisms of acquired resistance to AGX51

The efficacy of therapeutics can be hindered by cells developing resistance. Common mechanisms of acquired resistance include mutation of the drug-binding site or overexpression of the target. As described above, mutation of the binding site would likely result in loss of ID1 activity, thereby blocking this mechanism of escape. Overexpression of the ID1 protein could potentially outcompete the drug. However, as described above, endogenous *Id1* mRNA levels increase 20-fold in response to AGX51 exposure but the protein is still degraded efficiently making this type of resistance unlikely. Nonetheless, we tested the effects of AGX51 on HMLE *RAS Twist* cells lines overexpressing exogenous *ID1* mRNA (HMLE *RAS Twist ID1* cells). Western blot analysis revealed that despite significant ID1 overexpression, AGX51 treatment still dramatically reduced ID1 protein levels (Supplementary Fig. 11a). Similarly, 4T1 cells overexpressing exogenous *Id1* mRNA treated with AGX51 also showed a dramatic decrease in ID1 protein levels (Supplementary Fig. 11b). Furthermore, we generated an ID3-overexpressing 4T1 cell line (Supplementary Fig. 6d) and also observed efficient ID3 degradation in response to AGX51, as well as no evidence of measurable cell viability after long-term drug exposure (Supplementary Fig. 11c, d). Together these data suggest that AGX51 is a potent ID degrader, even in the context of *Id* mRNA overexpression.

**Fig. 6:**
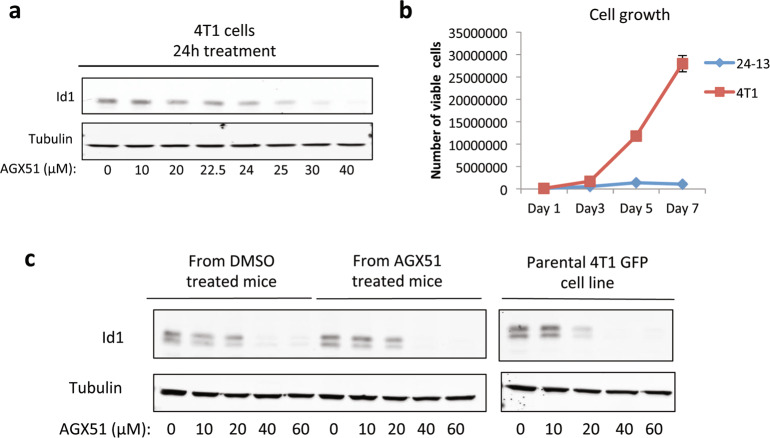
Generation of AGX51-resistant cell lines. **a** Western blot for ID1 on whole-cell lysates from 4T1 cells treated with 0–40 µM AGX51 for 24 h. **b** Cell growth of 4T1 cells and the AGX51 transiently resistant cell line clone 24-13, as determined by cell counting over 7 days. Mean of technical triplicates is shown with error bars representing SEM. **c** Cells were FACS isolated from the lungs of mice that were injected with 5 × 106 GFP-labeled 4T1 cells via tail vein and treated with 50 mg/kg bid or vehicle for 2 weeks. The isolated cells (from DMSO or AGX51-treated mice) and parental GFP-labeled 4T1 cells were treated with 0, 10, 20, 40, or 60 µM AGX51 for 24 h and whole-cell lysates were prepared. Western blot analysis for ID1 was then carried out. Tubulin serves as a protein loading control. See also Supplementary Figs. 11 and 12.

To determine whether tumor cells could spontaneously acquire resistance to AGX51, we sought to identify subpopulations of cells capable of continued growth in the presence of AGX51. 4T1 cells were exposed to various AGX51 concentrations, with media replenished every 2–3 days. Above 25 μM no resistant colonies of cells were observed even after 7 weeks of culture (~3 × 10^7^ cells tested). At 20 μM no overt cell death was observed, indicative of an extremely sharp dose–response curve. We then set up new experimental plates, treating the cells with either 24 or 22.5 μM, where ID loss after 24 h is not as pronounced (Fig. 6a). After 6 weeks in culture, we picked 30 seemingly viable clones but 29 eventually died after an average of ~5 passages. One clone survived the 24 μM condition but grew at reduced rates relative to the starting population (Fig. 6b), and eventually died after 11 passages.

None of the seven transiently resistant clones tested showed wild-type levels of ID1 although about half did show a possible rebound in ID3 (Supplementary Fig. 12a, b). No ID2 expression was detectable in these cells. In 4/7 transiently resistant cell lines, there was no/low ID1 expression, while 3/7 transiently resistant cell lines maintained ID1 expression at the level of cells treated with AGX51 for 24 h (Supplementary Fig. 12a). We also saw a decrease in Beta-catenin and Cyclin D1 levels in the transiently resistant cell lines (Supplementary Fig. 12c). Targeted sequencing of *Id1* and *Id3* coding regions in the three clones expressing detectable levels of ID1 and ID3 found no mutations, suggesting that mutation of the predicted compound-binding site, or other regions of the target proteins, is not the mechanism of transient resistance in these clones. While the growth rate of the clones cultured in 22.5 and 24 μM AGX51 was maintained for a time greater than that of the general population, this is not acquired resistance since all clones grew poorly and none remained viable after prolonged passaging. Furthermore, upon AGX51 removal, the cell growth rate and ID protein levels appeared to rebound to near-untreated 4T1 levels, and fell again upon re-treatment with 22.5 or 24 μM AGX51 (not shown). No acquired resistance was similarly observed in four other cell lines tested: HMLE *RAS Twist*, SW480, HCT15, and HCT116 (data not shown). Thus, despite some cells being capable of propagation in 22.5 or 24 μM AGX51, the cells are under strong growth-inhibitory pressure and fail to acquire genetic or epigenetic changes allowing for normal proliferation and *Id* expression in the presence of continued AGX51 exposure.

To determine if the small lung tumors that develop after AGX51 treatment are a result of acquired resistance in vivo we injected luciferase and GFP-labeled 4T1 cells into the tail veins of mice and 24 h later started treating the mice with AGX51 (50 mg/kg, bid) or vehicle for 2 weeks. This primary lung metastasis model showed significant anti-tumor efficacy with AGX51 as a single agent, as described above. Consistent with previous results, we observed a significant reduction in lung tumor burden in the AGX51 group. After 2 weeks of treatment, we harvested and dissociated the lungs and collected the GFP-positive cancer cells by FACS. After minimal passaging in cell culture (to obtain sufficient numbers of cells for western blot analysis), we re-challenged cells from both the AGX51- and DMSO-treated animals, as well as the parental cell line, with AGX51. We saw no difference in AGX51 sensitivity between parental (no in vivo exposure), DMSO, and AGX51 in vivo treated cells (Fig. 6c). The IC_50_ values for AGX51 in the isolated cells were very similar between DMSO- and AGX51-treated mice (23.48 μM and 22.34 μM, respectively). This result demonstrates that the development of small lung tumors after AGX51 treatment is not due to acquired resistance inherent in the cell population, but rather we suspect it is due to incomplete exposure of the tumor cells to AGX51 or the levels achieved in serum (~4 μM, see^48^) are insufficient to achieve complete regressions.

## Discussion

In the present study, we characterized the effects of a small-molecule antagonist of ID proteins, AGX51, in cancer cell lines and mouse models of breast and colonic neoplasia. Importantly, AGX51 leads to a significant reduction in ID protein levels in all cell lines tested, lung tumors, and ocular neovascularization^48^. ID protein destabilization after perturbation of its ability to antagonize E protein activity is consistent with genetic analyses in which coexpression of E proteins was shown to dramatically increase the stability of ID3^64,65^. AGX51 appears to act as an ID protein antagonist and degrader, thereby accounting for its ability to phenocopy *Id* genetic loss-of-function phenotypes in multiple settings.

Several approaches were taken to address the question of AGX51 target specificity. SILAC analysis showed that after short exposure to AGX51 when the ID proteins first begin to get degraded, only 14/2735 other proteins were underexpressed. This limited effect on the proteome is unlikely to be a purely off-target effect as AGX8, a molecule structurally unrelated to AGX51 (see Fig. 4f), also reduced ID protein levels and concomitantly the majority of the other proteins affected by AGX51. In addition, partial loss of *Id1* and *Id3* function reduced the IC_50_ of AGX51 (see Supplementary Table 1) and quiescent cells in which ID proteins are absent are resistant to AGX51-mediated killing. These data support ID proteins being the primary targets of AGX51. Finally, the ability of AGX51 to recapitulate the effects of *Id* loss-of-function mutations in various settings also suggests that these proteins are the primary targets in cells: AGX51 induces a G0/G1 arrest; inhibits the conversion of micro-metastases to macro-metastases in the 4T1 breast cancer model of experimental lung metastasis; with paclitaxel leads to regressions of paclitaxel-resistant primary breast tumor xenografts; phenocopies the consequences of *Id1/Id3* loss in two models of pathologic neovascularization^48^; and decreases the viability of PDA organoids, as was seen with shRNAs to the ID proteins^56^. AGX51 also showed anti-tumor activity in an autochronous model of colorectal neoplasia.

Despite repeated efforts, acquired resistance to AGX51 did not occur in cultured cells indicating that the escape frequency under our culturing conditions must be less than 1 in 10^7^ cells and perhaps significantly lower. These results suggest that despite the observed dramatic upregulation of *Id1* mRNA, ID protein levels are not restored efficiently nor are other pathways evoked, to compensate for ID loss. We also observed a lack of resistance in culture in cancer cells isolated from primary metastasis that persisted despite a 2-week AGX51 treatment regimen, suggesting that other factors such as drug exposure or the tumor microenvironment allowed for the survival of these cells. Another mechanism of resistance, mutating the binding site for the compound, is also not observed since this likely results in an inactive protein given the highly conserved nature of this pocket and inability to tolerate directed mutations that maintain activity at these *loci* in vitro^66^. Thus, by short-circuiting two major mechanisms of drug resistance (amplification of the ID target or mutation of the drug binding pocket) acquired resistance to AGX51 will be difficult to achieve and will probably serve to enhance the efficacy of AGX51 in a therapeutic setting.

The observed need for combining AGX51 treatment with chemotherapy to see anti-tumor efficacy in established mammary fat pad and lung tumors reflects the situation seen in genetic analyses where Her2/neu breast tumor regression required chemotherapy in *Id* knockout mice^59^. The reasons for this are not yet understood but it may be due to there being very few ID-positive resting stem cells, as has been observed in other models^35^, requiring combination treatment to hit two tumor populations (ID-positive and ID-negative) to see significant effects. Future studies will be required to resolve this question. We note that the use of cannabidiol (CBD), which reduces *Id1* transcription, has been reported to inhibit aggressive breast cancer cell behavior as a single agent^67^. This may in part reflect broad CBD activity against a variety of factors including β-catenin and protein kinase B (AKT) among others^68^.

ID protein expression in normal adult tissue stem cells is required in response to various stresses: in colonic stem cells, ID1 is required to minimize inflammation after DSS-induced chemical injury in a model of colitis^69^; in long-term repopulating hematopoietic stem cells they are required for optimal cellular expansion after ablation of the progenitor pool or to maintain long-term serial transplantation^70,71^; in neural stem cells, ID1 and ID3 are expressed in quiescent stem cell populations but show pronounced phenotypes only when those cells are called into cell cycle upon AraC ablation of progenitor cells^72^; ID proteins are expressed at very low levels in the liver but are reactivated during regeneration in response to injury^73^; ID1 is not expressed in normal human epidermis but may be a key regulator of epidermal wound healing^74^. These observations likely account for the low toxicity of AGX51 in animals but suggest caution in the use under conditions of duress. In cancer, a similar phenomenon may be taking place since in glioma, ID1, and ID3 appear to be in a resting stem-like cell which resists killing of cycling cells by ionizing radiation^35^. We have recently observed ID1 associated with a resting state in cholangiocarcinoma and TNBC (Robert Benezra 2020, “ID1 expression in resting cancer stem cells”, unpublished). It will be of great interest to determine if combining standard cytotoxic therapies and AGX51 would kill the ID1-negative cycling population and the ID1-positive stem-like cells called into cycle, respectively.

Importantly, other PPIs have been shown to be critical for ID activity in the stem cell character of other tumors: ID2, for example, facilitates stemness in glioma by its association with the VHL complex and antagonism of the ubiquitin-mediated proteolysis of Hif2α^75^, so determining the effects of AGX51 on this pathway will be of great interest. Given the complete suppression of gliomagenesis upon loss of *Id1*, *Id2*, and *Id3* in a murine model^76^ and the ability of AGX51 to antagonize all members of the family, it is likely that sufficient exposure of these brain tumors to AGX51 will provide some therapeutic benefit. It should be noted that in some cancer subtypes ID proteins may play tumor-suppressive roles. As stated above, in Burkitt’s lymphoma *ID3* is frequently mutated and appears to act as a tumor suppressor^34^. ID4, for example, may be a tumor suppressor in ER+ breast cancer^77^ and ID2 may reduce invasiveness in TNBC^78,79^. Thus, the lack of specificity of AGX51 for individual ID family member may complicate its use in certain settings.

In conclusion, the first-in-class small-molecule antagonist of the ID protein family, AGX51, dramatically reduces ID protein levels and cancer cell viability, as well as inhibits tumor growth. The ability of AGX51 to phenocopy the consequences of *Id* genetic loss suggests that in addition to being a useful biologic tool, AGX51 can serve as a lead compound that can be developed to provide clinical benefit for a variety of Id-related cancers.

## Methods

### Electrophoretic mobility shift assays

EMSAs were carried out on whole-cell lysates from AGX51-treated cells and the EMSA was performed as described previously^36^.

### Cell lines

The 4T1 murine mammary tumor cell line, MDA-MB-157, MDA-MB-436, MDA-MB-231, MDA-MB-453, MDA-MB-361, BT-474, SK-BR-3, MCF-7, T47-D, and HCT116 were purchased from ATCC (Manassas, VA, USA) and grown in RPMI or DMEM (Dulbecco’s Modified Eagle medium) media supplemented with 10% FBS (fetal bovine serum), 1% penicillin–streptomycin and 1% L-glutamine. MDA-MB-231 cells used in xenograft studies were obtained from NCI NIH (Bethesda, MD). Luciferase-labeled 4T1 cells were described previously^80^, as were luciferase- and GFP-labeled 4T1 cells^81^. The 4T1 cells’ overexpression *Id1* were derived by transducing cells with pBabe-*Id1* plasmids as described previously^47^. HMLE *RAS Twist* and HMLE *RAS Twist ID1* cells were described previously^47^ and grown in MEBM (Mammary Epithelial Cell Growth Medium) (Lonza), supplemented with BPE (bovine pituitary extract) (70 μg/mL), hEGF (human epidermal growth factor) (5 ng/mL), hydrocortisone (0.5 μg/mL), insulin (5 μg/mL), GA-1000 (Gentamicin 30 μg/mL and Amphotericin 15 ng/mL), hTransferrin (5 μg/mL), and isoproterenol (6 μM). 4T1 cells were authenticated by short tandem repeat analysis and karyotyping. Other cell lines have not been authenticated since being obtained from their source. The cell lines were not tested for mycoplasma contamination recently but routine testing had been negative for years prior and no growth defects associated with such contamination have been observed in any of the cell lines used in the studies presented.

### Patient-derived xenograft cell lines BR7, BR11, and IBT

PDXs were established directly from breast cancer bone metastases specimens surgically resected from patients with informed consent (IRB protocol #97-094). BR7 and BR11 specimens are obtained from ER-positive (HER2-negative and PR-negative) metastatic breast cancer patients, while the IBT specimen was obtained from a metastatic, TNBC patient. Fresh tumor tissues were quickly washed with ice-cold PBS and minced into about 1 mm^3^ pieces in MEM medium (without FBS) using sterile razor blades. A fraction of the minced original tumor tissues was incubated with collagenase/hyaluronidase enzyme mix (1000 Units, Voden Medical) in MEM medium without FBS (5 mL per 250 mg tissue) for 2–4 h. The dissociated tumor tissues were then filtered through a 70 μm nylon filter, cells were concentrated by centrifugation at room temperature and seeded to derive respective primary cell cultures in MEM medium with 3% FBS (Sigma). The primary cell cultures were transduced with the fluorescent td tomato-/EGFP-luciferase fusion protein expressing lentiviral vectors for 18–24 h, the primary cells were then maintained in MEM media supplemented with 3% FBS, 1% penicillin–streptomycin and 1% L-glutamine. Aliquots of the primary cell cultures were also cryopreserved following a minimal number (3–4) of in vitro passages.

### E protein knockout cell experiments

DN3/4 thymocytes were isolated from male and female E2A^fl/fl^ HEB^fl/fl^ E8_III_-cre mice^50,51^ by bead-based negative selection. Briefly, single-cell suspensions of total thymocytes were stained with biotinylated antibodies against CD4, CD8, CD11b, CD11c, CD16/32, CD19, CD44, c-Kit, B220, Ly6C, GR-1, NK1.1, TER119, and TCRgd. Antibody-labeled cells were then depleted with SPHERO Streptavidin-coated magnetic beads (Spherotech, cat. SVM-40-10) using the Dynabeads biotin binder beads negative isolation protocol (Invitrogen). Purified DN3/4 cells were co-cultured on an OP9-DL1 monolayer at 5.0 × 105 cells/mL in MEM alpha supplemented with 10% FBS, 1× penicillin/streptomycin and 5 ng/mL recombinant IL-7 with 60 μM AGX-51 or DMSO vehicle. After 24 h, DN3/4 cells were harvested and RNA was purified using the Direct-Zol microprep kit (Zymo Research, cat. R2061). cDNA was generated using SuperScript III reverse transcriptase (Invitrogen) with random hexamers according to the manufacturer’s protocol. qPCR was performed using PowerTrack Sybr Green Master Mix (Invitrogen) with an Eppendorf Mastercycler ep realplex thermal cycler. Expression was calculated relative to 0 μM AGX-51 and normalized to actin.

The primers used for qPCR are as follows: Id2-F: CGACCCGATGAGTCTGCTCTA; Id2-R: GACGATAGTGGGATGCGAGTC; Id3-F: GCCTCTTAGCCTCTTGGACG; Id3-R: GTTCCGGAGAGAGCTCAGC; actin-F: AAGGCCAACCGTGAAAAGAT; actin-R: GTGGTACGACCAGAGGCATAC.

### Pancreatic ductal adenocarcinoma cells and organoids

The human pancreatic cancer cell line Panc1 was obtained from ATCC in 2012, and A21 was obtained in 2016 from Dr. Christine Iacobuzio-Donahue^82^. Mouse pancreatic cancer lines 806 (KrasG12D; Ink4a−/−; Smad4−/−) and NB44 (KrasG12D; Ink4a−/−) were obtained from Dr. Nabeel Bardeesy, and 4279 (KrasG12D; Ink4a−/−) was generated in Dr. Joan Massague’s laboratory. Mouse pancreatic organoid cell lines T7 and T8 were obtained from Dr. David Tuveson^83^. Pancreatic spheroids were grown in Ultra Low Attachment Culture plates (Corning) in DMEM supplemented with Glutamax (2 mM) and heparin (5 µg/mL). Pancreatic organoids were embedded in Matrigel with Advanced DMEM/F12 (Gibco, 12634-028) supplemented with B-27 (Life Technologies, 12587-010), HEPES (10 mM), 50% Wnt/R-spondin/Noggin-conditioned medium (ATCC, CRL-3276), Glutamax (Invitrogen, 2 mM), N-acetyl cysteine (Sigma, 1 mM), nicotinamide (Sigma, 10 mM), epidermal growth factor (Peprotech, 50 ng/mL), gastrin (Sigma, 10 nM), fibroblast growth factor-10 (Peprotech, 100 ng/mL), A83-01 (Tocris, 0.5 µM) as previously described^83^. All cell lines and organoids were maintained at 37 °C and 5% CO_2_. Cell viability was measured using Cell Titer-Glo (Promega) according to manufacturer’s instructions.

### Generating *Id* knockdown and overexpression cell lines

To generate 4T1 cells containing inducible short hairpins against *Id1* or *Id3*, short hairpins against *Id1* (TGCTGTTGACAGTGAGCGAGCGTGTTTCTGTTTTATTGAATAGTGAAGCCACAGATGTATTCAATAAAACAGAAACACGCGTGCCTACTGCCTCGGA) and *Id3* (TGCTGTTGACAGTGAGCGCGCCCTGATTATGAACTCTATATAGTGAAGCCA CAGATGTATATAGAGTTCATAATCAGGGCATGCCTACTGCCTCGGA) were cloned into the tet-regulated all-in-one retroviral miR-E expression vector RT3REVIR^84^. Retroviral particles were produced by lipofectamine-mediated transfection into 293 GP2 cells. Forty-eight hours after transfection, virus-containing media was collected, filtered and added to 4T1 cells in the presence of 8 μg/mL polybrene. Transduced cells were selected for Venus positivity by FACS. To induce short hairpin expression cell culture media was supplemented with doxycycline (5 μg/mL). RT3REVIR expressing a short hairpin against Renilla was used as a control (shCTL) in experiments.

Human cDNA ID3 (hId3) or ID1 (hId1) was cloned into the lentiviral vector pWPI, obtained from Addgene catalog number: 12254 (addgene.org). A day before transfection, 293T cells were seeded in DMEM media on poly-L-lysine-treated plates to achieve 90% confluency then 18.5 μg of psPax2, 12 μg of VSVG, and 152.4 μL of CaCl_2_ (2 M) were mixed and brought to a volume of 600 μL with molecular biology-grade water; 600 μL of 2× HBS was added, mixed and incubated for 60 s. Transfection mix was added to 8.8 mL of pre-warmed D10 media (DMEM, 10% FBS, 1% penicillin and streptomycin, 1% L-glutamine, 1% 100 mM sodium pyruvate, 1% sodium bicarbonate) and added to 293T cells. Plates were incubated at 37° C overnight. On day 3, ~12 h post transfection, cells were rinsed with D10 media before incubating in D10 media with 50 μg/mL G418. At ~16 h post transfection, media was replaced with VPM media (Ultraculture media, 1% penicillin and streptomycin, 1% L-glutamine, 1% 100 mM sodium pyruvate, 1% sodium bicarbonate, 5 mM sodium butyrate). On day 4, ~46 h post transfection, viral supernatants were collected into 15 mL tubes, centrifuged at 1100 rpm for 5 min, passed through 0.40 μM filters into clean tubes and aliquoted for storage at −8 °C until transduction.

### Infection and western blot analysis

One day before infection, 4T1 cells were plated in DMEM media in 10 cm dishes for 50% confluency the next day. On day 2, 1 mL of viral particles and 8 μL polybrene (10 mg/mL stock) were added to 9 mL of DMEM media, mixed and added to the corresponding dish of 4T1 cells. After overnight incubation, media on infected plates was changed. On day 4 or 5, 4T1 cells transduced with lentiviral particles carrying either pWPI-hId3, pWPI-hId1, or pWPI were subjected to FACS sorting for a high GFP intensity. FACS sorted cells were collected and cultured for western blotting analysis. 4T1-pWPI-hId3 cells, 4T1-pWPI-hId1 cells, or 4T1-pWPI cells were analyzed for the expression of exogenous Id3 protein by western blot. Cells were lysed using the protein extraction buffer containing protease inhibitors (Roche, ref: 04 693 132 001) and 35–40 μg of the total protein lysate was loaded per lane of 15% SDS-PAGE. ID3 protein was detected using anti-ID3 antibodies with the dilution of 1:1000 using the Li-CoR system.

### Immunoblotting

For immunoblotting, cells were collected by trypsinization, washed with PBS and lysed in homogenization buffer (0.3 M sucrose, 10 mM Tris (pH 8.0), 400 mM sodium chloride, 3 mM magnesium chloride, 0.5% NP40/IGEPAL, 100 μg/mL Aprotinin+ Protease Inhibitor Cocktail (Roche # 11 836 153 001). Proteins were separated by SDS-PAGE, transferred to a membrane (LI-COR), probed with primary antibodies overnight at 4 °C, and probed with secondary antibodies (LI-COR) for 1–2 h at room temperature. Proteins were visualized using the LI-COR Odyssey Infrared Imaging detection system. The following primary antibodies were used: Id1, Id2, Id3, Id4 (195-14, 9-2-8, 17-3, 82-12, respectively, all from Biocheck, all used at 1:1000), Phospho-Histone H3 (9701, Cell Signaling, 1:500), Cyclin D1 (2978, Cell Signaling, 1:1000), Beta-catenin (9562, Cell Signaling, 1:1000), Cdk4 (sc-260, Santa Cruz, 1:200), Mcl1 (5453, Cell Signaling, 1:500), Ube2C (14234, Cell Signaling, 1:500), Sqstm1 (H00008878-M01, Abnova, 1:1000), Alpha-catenin (C2081, SIGMA-ALDRICH, 1:5000), E-cadherin (3195 Cell Signaling, 1:1000), Vimentin (5741, Cell Signaling, 1:1000), Snail (3879, Cell Signaling, 1:1000), Twist (ab50887, Abcam, 1:200), Zeb1 (NBP1-05987, Novus Biologicals, 1:2000), Actin (A2066, SIGMA, 1:10,000), Tubulin (T4026, SIGMA, 1:10,000). Western blot quantification was carried out using channel 700 and channel 800 intensity data from Odyssey application software version 3.0.30 (LI-COR), subtracting blank values and normalizing to tubulin. All individual blots were derived from the same experiment and processed in parallel.

### qRT-PCR

RNA was extracted using the RNeasy kit (Qiagen, Valencia, CA, USA) and cDNA was generated from 1 μg of RNA using SuperScript IV First-Strand Synthesis System (Invitrogen, Grand Island, NY, USA). Quantitative PCR was performed using SYBR Green QuantiTect Primer Assay (Qiagen) according to manufacturer’s instructions in a 7900HT Fast-Real Time PCR System Instrument (Applied Biosystems, Grand Island, NY, USA). Primer pairs for the individual genes were obtained from the bioinformatically validated QuantiTect Library and are as follows: *Id1* (QT01743756), *Actb* (QT00095242), *Ctnnb1* (QT00160958), *Ccnd1* (QT00154595), *Cdk4* (QT00103292), *Mcl1* (QT00107436), *Idh2* (QT00147301), *Idh3b* (QT00117341), *Nbas* (QT00280483), *Ifrd1* (QT00093401), *Atp5h* (QT00175070), *C1qbp* (QT01057777), *Cox5a* (QT00164234), *Dlst* (QT00163275), *Glud1* (QT00103411), *Vim* (QT00159690). The fold changes in gene expression were calculated using the delta-delta CT method.

### Cell viability assays

Alamar blue cell viability assay (Invitrogen) was carried out according to manufacturer’s instructions. Briefly, 5000 4T1 cells were seeded in a 96-well plate, the next day they were treated with 40 μM AGX51 for 24 h, after which point a 1:10 dilution of Alamar blue cell viability reagent was added to the cells and absorbance was measured 2, 3, 4, 5, and 6 h after addition of the reagent using a plate reader (Synergy 2, BioTek).

For IC_50_ determination, cancer cell lines were seeded in a 96-well plate (5000 cells per well). After overnight incubation, cells were treated with AGX51 (0, 5, 10, 20, 40, 60 μM) and incubated for 24, 48, and 72 h, each condition was done in triplicate. At each time point, 40 µL of MTT reagent (5 mg/mL) was added per well and the cells were incubated for 4 h. Following incubation, media was aspirated and 200 µL DMSO was added per well. Absorbance was then measured at 570 nm using a plate reader (Synergy 2, BioTek). Data were analyzed and visualized using GraphPad Prism.

To determine whether vitamin E could rescue AGX51 cell viability effects, cells were pretreated with vitamin E (( ± )-α-Tocopherol, SIGMA) (50 or 100 µM) for 1 h. Following the 1 h treatment, cells were treated with 40 µM AGX51/DMSO for 24 h. Cell viability was then determined by trypan blue exclusion and cell counting.

To measure ferroptosis, 4T1 cells were seeded in 150 μL DMEM media per well of a 96-well plate and treated with vehicle, 1 μM erastin (Millipore Sigma, 329600), 1 μM ferrostatin-1 (Xcessbio, M60042-2), 40 μM AGX51, 1 μM erastin + 1 μM ferrostatin, and 40 μM AGX51 + 1 μM ferrostatin-1. Celltiter-Glo (Promega G8461) was used to determine cell viability using the manufacturer’s instructions. Ferroptosis was observed 12 h after treatment.

To measure necroptosis, 4T1 cells were seeded in 150 μL DMEM media per well of a 96-well plate and treated with vehicle, 40 μM AGX51, 10 ng/mL TNF (R&D Systems, 410-MT-010) + 20 μM z-VAD-FMK (Enzo Life Sciences, ALX-260-020-M001), 30 μM necrostatin-1 (Millipore Sigma, 480065-5MG), 10 ng/mL TNF + 20 μM z-VAD-FMK + 30 μM necrostatin-1, and 40 μM AGX51 + 30 μM necrostatin-1. Celltiter-Glo (Promega G8461) was used to determine cell viability using the manufacturer’s instructions. Necroptosis was observed 12 h after treatment.

Cell proliferation of the AGX51-resistant clone (24-13) was determined by plating 60,000 24-13 or 4T1 cells and collecting and counting the cells on days 1, 3, 5, and 7 after plating using a hemocytometer with the addition of trypan blue to exclude dead cells.

To assess synergistic effect of AGX51 and Paclitaxel (PCT) in vitro, MDA-MB-231 cells were grown in two replicates in DMEM (Dulbecco’s Modified Eagle medium) media supplemented with 10% FBS, 1% L-glutamine and 1% penicillin–streptomycin. When cells reached confluency suitable (40–50%) for drug treatment in 48 h, the cells were treated with DMSO (vehicle), or predetermined optimum PCT concentration (20 nM) alone or various concentrations of AGX51, or combination of various concentrations of AGX51 with PCT for 24 h. The cell numbers were counted using Trypan Blue exclusion to assess the effect of the drugs.

### Cell cycle analysis

Cells were treated with AGX51 or DMSO, or vitamin E (as described above), collected by trypsinizaton, washed with 1× PBS, resuspended in 500 μL 1× PBS and then diluted with 6 mL 70% ethanol and stored at −20 °C until analysis. For cell cycle analysis cells were centrifuged 1000 rpm for 5 min, washed with 1× PBS and then resuspended in 0.5 mL PI/RNase staining buffer (550825, BD Biosciences), incubated for 15 min at room temperature and analyzed by flow cytometry (LSR II).

AGX51- or DMSO-treated cells were stained with 10 μM BrdU reagent (550891, BD Biosciences) for 1 h. The cells were then collected, resuspended in 70% ethanol, washed with 1× PBS, incubated with 2 M HCl, washed in 1× PBS and 1× PBS-Tween-BSA, and then incubated with an Alexa Fluor 488 anti-BrdU antibody (558599, BD Biosciences) for 15 min and finally resuspended in RNase/PI buffer (550825, BD Biosciences) and analyzed by flow cytometry.

### Annexin/PI staining

To detect apoptosis and necrosis we used the BioVision Annexin V-FITC Apoptosis detection kit according to the manufacturer’s instructions. Briefly, 4T1 cells were treated with 40 μM AGX51, trypsinized and resuspended in 1× Binding Buffer to which 5 μL Annexin V-FITC and 5 μL propidium iodide (PI) (50 μg/mL) were added. The cells were incubated for 5 min protected from light and then analyzed by flow cytometry (LSR II).

### Measuring ROS

4T1 cells were treated with 40 μM AGX51 for 24 h, and then 10 μM 2ʹ,7ʹ-dichlorodihydrofluorescein diacetate (H2DCFDA) (Life Technologies) was added to the cells and they were incubated for 1 h. Cells were washed twice with PBS, trypsinized and resuspended in PBS–5% FBS. H2DCFDA is converted to fluorescent DCF upon oxidization, and serves as a readout for ROS generation. Fluorescence was measured using flow cytometry (LSR II). As a positive control for ROS production, cells were treated with 100 μM of H_2_O_2_.

### Testing the effects of AGX51 on quiescent cells

To induce a senescent state prior to AGX51 treatment, 4T1 cells were plated to reach high density at the time of AGX51 treatment. The day after plating the cells, media was changed to low-serum media (1% FBS). After 24 h in low-serum media, the cells were treated with AGX51. Cells plated at standard density and maintained in 10% FBS served as controls. Following the 24 h of AGX51 treatment, cell numbers were determined by cell counting and trypan blue exclusion and cells collected for whole-cell lysate extraction for western blot analyses.

### Generation of SILAC cell lines, and in-gel digestion for mass spectrometry

4T1 cells were grown in DME media supplemented with 10% FBS and penicillin and streptomycin, and either unlabeled L-arginine (Arg0) and L-lysine (Lys0) at 50 mg/L or equimolar amounts of the isotopic variants [U-13C6,15N4]-L-arginine (Arg10) and [U-13C6]-L-lysine (Lys6) (Cambridge Isotope Laboratories). After five cell doublings, cells were >99% labeled with the isotopes, as determined by mass spectrometry. Cell lysates were prepared as described above; 1 mg of total protein from each light and heavy labeled cell line was mixed and the total 2 mg of protein was fractionated using 4–12% gradient SDS-PAGE. Each gel lane cut in equal pieces and gel slices were washed with 1:1 (Acetonitrile: 100 mM ammonium bicarbonate) for 30 min. Gel slices were then dehydrated with acetonitrile for 10 min until gel slices shrunk and excess acetonitrile was removed before slices were dried in speed-vac for 10 min without heat. Gel slices were reduced with 5 mM DTT for 30 min at 56 °C in an air thermostat, then chilled to room temperature, and alkylated with 11 mM IAA for 30 min in the dark. Gel slices were washed with 100 mM ammonium bicarbonate and acetonitrile for 10 min each. Excess acetonitrile was removed and dried in a speed-vac for 10 min without heat and gel slices were rehydrated in a solution of 25 ng/μL trypsin in 50 mM ammonium bicarbonate on ice for 30 min. Digestions were performed overnight at 37 °C in an air thermostat. Digested peptides were collected and further extracted from gel slices in extraction buffer (1:2 v/v) 5% formic acid/50% acetonitrile) at high-speed shaking in an air thermostat. Supernatant from both extractions were combined and dried down in a vacuum centrifuge. Peptides were desalted with C18 resin-packed stage-tips. Desalted peptides were lyophilized and stored at −80 °C until further use.

### LC-MS/MS and whole-proteome analysis

Desalted peptides were dissolved in 3% acetonitrile/0.1% formic acid and were injected onto a C18 capillary column on a nano ACQUITY UPLC system (Waters) that was coupled to the Q Exactive plus mass spectrometer (Thermo Scientific). Peptides were eluted with a non-linear 200 m gradient of 2–35% buffer B (0.1% (v/v) formic acid, 100% acetonitrile) at a flow rate of 300 nL/min. After each gradient, the column was washed with 90% buffer B for 5 min and re-equilibrated with 98% buffer A (0.1% formic acid, 100% HPLC-grade water) for 4 min. MS data were acquired with an automatic switch between a full scan and 10 scan data-dependent MS/MS scan (TopN method). Target value for the full scan MS spectra was 3 × 106 charges in the 380–1800 *m*/*z* range with a maximum injection time of 30 ms and resolution of 70,000 at 200 *m*/*z* in profile mode. Isolation of precursors was performed with 1.5 *m*/*z*. Precursors were fragmented by higher energy C-trap dissociation (HCD) with a normalized collision energy of 27 eV. MS/MS scans were acquired at a resolution of 17,500 at 200 *m*/*z* with an ion target value of 5 × 104 maximum injection time of 60 ms and dynamic exclusion for 15 s in centroid mode. A total of 6346 proteins (5604 clusters) with an FDR < 1% were identified across the three independent pairs of samples analyzed (4 h treatment of 4T1 cells with AGX51 or DMSO vehicle control). The data were analyzed using Scaffold Q+ version 4.4.5 (Proteome Software, Portland, Oregon, USA). Briefly, fold differences of the fold change ratios were calculated for each pair of samples (AGX51 vs. DMSO) and proteins with fold differences (at least 1.35-fold) in the same direction of change (overexpressed or underexpressed) in each of the three pairs were identified (see Supplementary Table 2).

### Animal studies

Animal studies were carried out in accordance with institutional regulations (IACUC protocol 06-10-025) in a non-blinded fashion. Weight measurements and standard blood analyses were carried out on 8–12-week-old male CD1 mice (*n* = 5 per treatment group) dosed q5d with DMSO, 15 mg/kg qd paclitaxel, 60 mg/kg AGX51 bid, or a combination of paclitaxel and AGX51. Blood samples were collected by retro-orbital puncture on day 6, 12 h after the last dose. Plasma was analyzed for clinical chemistry (albumin, alkaline phosphatase, alanine aminotransferase, amylase, total bilirubin, blood urea nitrogen, calcium, phosphorus, creatine, glucose, sodium, potassium, total protein, and globulin) and hematology (white blood cell, lymphocyte, monocyte, granulocyte, red blood cell, hemoglobin, hematocrit, and platelet) concentrations.

Orthotopic mammary fat pad tumors were generated by injecting 5 × 106 MDA-MB-231 cells (in 1:1 PBS:Matrigel) into the right caudal mammary fat pad of 8–12-week-old, female athymic nu/nu mice (*n* = 30). Mice were obtained from Simonsen Laboratories (Gilroy, CA). Tumors were allowed to grow to ~100 mm^3^, at which point they were divided into 6 groups of 5 mice with approximately the same tumor burden and treatment was initiated. Group 1 was vehicle (DMSO) control, group 2 received 5 days of 15 mg/kg paclitaxel, group 3 received 19 days of 60 mg/kg AGX51 bid, group 4 received a combination of paclitaxel and 6.7 mg/kg AGX51 bid, group 5 received a combination of paclitaxel and 20 mg/kg AGX51 bid, group 6 received a combination of paclitaxel and 60 mg/kg bid, and group 7 received a combination of paclitaxel and 60 mg/kg AGX51 but only received AGX51 for 7 days (all other AGX51 groups had 19 days of AGX51 treatment). The AGX51 and paclitaxel were administered i.p. Tumor volumes were determined throughout the study using a digital caliper and the formula: tumor volume = ½ (length × width^2^) where the greatest longitudinal diameter is the length of the tumor and the greatest transverse diameter is the width. At study termination the mice were sacrificed by cervical dislocation.

Lung metastases were generated by injecting 6–8-week-old, female, Balb/c mice with 50,000 luciferase-labeled 4T1 cells into the tail vein. Twenty-four hours after tail vein injections, mice were treated with DMSO vehicle or 50 mg/kg AGX51 qd (5 mice per treatment group) by i.p. injection. One mouse in the AGX51-treatment group died from the isoflurane anesthesia during the first luciferase imaging session. In another experiment mice were treated bid. The bid experiment was performed three times. In the first experiment *n* = 5 mice per group, in the second experiment *n* = 4 vehicle-treated mice and *n* = 8 AGX51-treated mice, and in the third experiment *n* = 5 mice per group. No statistically significant differences in the effect was observed across experiments. Development of lung metastases was monitored using the IVIS-200 in vivo imaging system. In the experiment testing the effects of AGX51 on established lung metastases (*n* = 5 mice per group), once evidence of lung metastases was observable by in vivo imaging, mice were divided into 4 groups of 5 mice with approximately the same tumor burden per treatment group. The groups were: group 1, vehicle; group 2, 50 mg/kg AGX51 bid; group 3, 15 mg/kg paclitaxel qd for 5 days; and group 4, combination of AGX51 + paclitaxel. At the end of the experiments mice were euthanized and tissues were collected for further analyses. Lung tumor burden was quantified in a blinded fashion by a pathologist.

To assess effects of AGX51 on seeding of the lungs, 20 6–8-week-old female Balb/c mice were injected with GFP-labeled 4T1 cells as described above and on the next day treated with AGX51 (50 mg/kg) (*n* = 10) or DMSO (*n* = 10). Twenty-four hours later, one set of mice (5 from the AGX51 group and 5 from the DMSO group) was euthanized and their lungs collected for histological analysis. After 48 h of treatment, the remaining 10 mice were euthanized and their lungs collected for histological analysis.

To assess whether in vivo treatment results in acquired resistance to AGX51, 10 6–8-week-old female Balb/c mice were tail vein-injected with GFP-labeled 4T1 cells and treated with AGX51 or DMSO for 2 weeks as described above. After 2 weeks of treatment, the mice were euthanized and the lungs were dissociated according to the kit instructions (Mouse Lung Dissociation Kit, MACS Miltenyi Biotec) and the cells were sorted for the GFP-positive fraction. The cells were allowed to expand to generate enough cells for AGX51 treatment and western blot analysis, at which point the cells were harvested and whole-cell lysates were made.

Spontaneous colon tumors were induced by treating 30, 4-week-old male A/J mice (Jackson Laboratory) with AOM (10 mg/kg; Sigma Aldrich) once a week by i.p. injection for 6 weeks. Mice were maintained on AIN-93G purified diet (Research Diets) for the duration of the experiment. After a 3-week treatment break, mice were treated i.p. with DMSO (*n* = 13) or AGX51 (*n* = 11) (15 mg/kg) bid for 3 weeks. Following the last injection, the mice were euthanized and colon tumors were formalin fixed to assess tumor burden. Tumor numbers and size were determined in whole mounts of the tissues following methylene blue staining.

All tumor growth and treatment studies complied with MSKCC Institutional Guidelines and received IACUC approval.

### Immunohistochemistry

Slides mounted with sections from formalin-fixed and paraffin-embedded mouse tissues were deparaffinized, rehydrated, and stained with hematoxylin and eosin (H&E), as well as with the following primary antibodies: Id1 (37-2, Biocheck 1:1000), Vimentin (5741, Cell Signaling 1:500), Cyclin D1 (2978, Cell Signaling 1:500). Detection was performed using the Vectastain Elite ABC kit (Vector), diaminobenzidine was used as the chromogen, and Harris Modified Hematoxylin was used as the nuclear counterstain.

### Immunofluorescence

The lungs from mice from the seeding experiment were perfused with 1× PBS, followed by 4% PFA. The lungs were then removed, washed in cold PBS, and fixed in 4% PFA overnight. The lungs were dehydrated in a sucrose solution series (20–30%) and then embedded in OCT and flash frozen. Ten micron cryosections were then boiled for 10 min in citrate buffer, for antigen retrieval, washed with 1× PBS, blocked in PBS, 10% goat serum, 0.05% triton and then incubated with anti-GFP antibody (GFP-1020, Aves labs, Inc.) overnight at 4°. The next day the slides were washed and incubated with Alexa Fluor 555 anti-chicken antibody (A-21437, Thermo Fisher Scientific), washed and counterstained with DAPI followed by mounting and coverslip application. The slides were scanned and ten random fields from each section were analyzed for GFP-positive cell content.

### AGX51-resistant clone generation

To generate resistant cell lines, cells were maintained in AGX51-containing media (20–40 μM range). AGX51-containing media was replaced every 2–3 days. Any clones that grew out were expanded, and maintained in AGX51-containing media. Transiently resistant clones expressing *ID1/ID3* had their cDNA sequenced for the coding regions of *ID1* and *ID3* by Sanger sequencing using the primers:

Id1-F:CTTCTTGTTCTCTTCCCACA

Id1-R:GATCAAACCCTCTACCCACT

Id3-F:CACTGTTTGCTGCTTTAGGT

Id3-R:CGTTGAGTTCAGGGTAAGTG

### Statistical analyses

Three replicates were generally used for each experimental condition for in vitro experiments and 5 mice per group were typically used in each mouse experiment. The sample sizes were determined based on an expected large effect size. With 3 replicated per condition, an effect size as small as 3 can be detected with 80% power at a two-sided significance level of 0.05 using a two-sample *t*-test. With 5 mice per group, an effect size as small as 2 can be detected with 80% power at a two-sided significance level of 0.05 using a two-sample *t*-test. Additional experiments may be performed when larger variation in data was observed and data were pooled for analysis. In general, Welch’s *t*-test was used to examine differences between two groups. ANOVA was used to examine differences across multiple experimental groups. Data may be transformed to ensure the underlying normality assumptions were met. For example, for tumor number data, square root transformation was used. Weighted linear regression analysis was used when heteroscedasticity was observed and data points in each group were typically weighted by the reciprocal of the standard deviation of data in each group. For data pooled from multiple experiments, the model included both experiments and experiments by treatment group interaction as covariates to account for potential differences in experiments. Significance of linear contrasts of interest was assessed based on estimates obtained from the weighted least squares. *Q*–*Q* plot of the residuals was examined to ensure the underlying model assumptions were met. *p*-value < 0.05 was considered statistically significant. For the colon tumor counts, the data were analyzed using the Wilcoxon rank sum test.

### Reporting summary

Further information on research design is available in the Nature Research Reporting Summary linked to this article.

## Supplementary information

Supplementary information.

Reporting summary.

## Data Availability

The data generated and analyzed during this study are described in the following data record: 10.6084/m9.figshare.14229986^85^. The proteomics data of anti-tumor effects of an ID antagonist with no observed acquired resistance are openly available in the PRIDE Archive via the following accession: https://identifiers.org/pride.project:PXD024593^86^. The raw data underlying the following figures are openly available as part of the data record: 1E, 2A, 3B, C, D, 4A, E, 5A, B, F, G, 6B, S1A, D, F, G, S2E, I, J, S5A, B, S6, S7A, S8C. A comprehensive list of which file is associated with each figure is also included in the Excel spreadsheet ‘Wojnarowicz_et_al_underlying_data_files_list.xlsx’. All uncropped western blots are in the Supplementary Information section.
