# Supplementary material for: Anti-tumor effects of an ID antagonist with no observed acquired resistance
Source: NPJ Breast Cancer. 2021 May 24;7:58. doi: 10.1038/s41523-021-00266-0 (PMC8144414; doi:10.1038/s41523-021-00266-0)
Supplement: Supplementary file 1 — Supplementary information. [file 41523_2021_266_MOESM1_ESM.pdf]

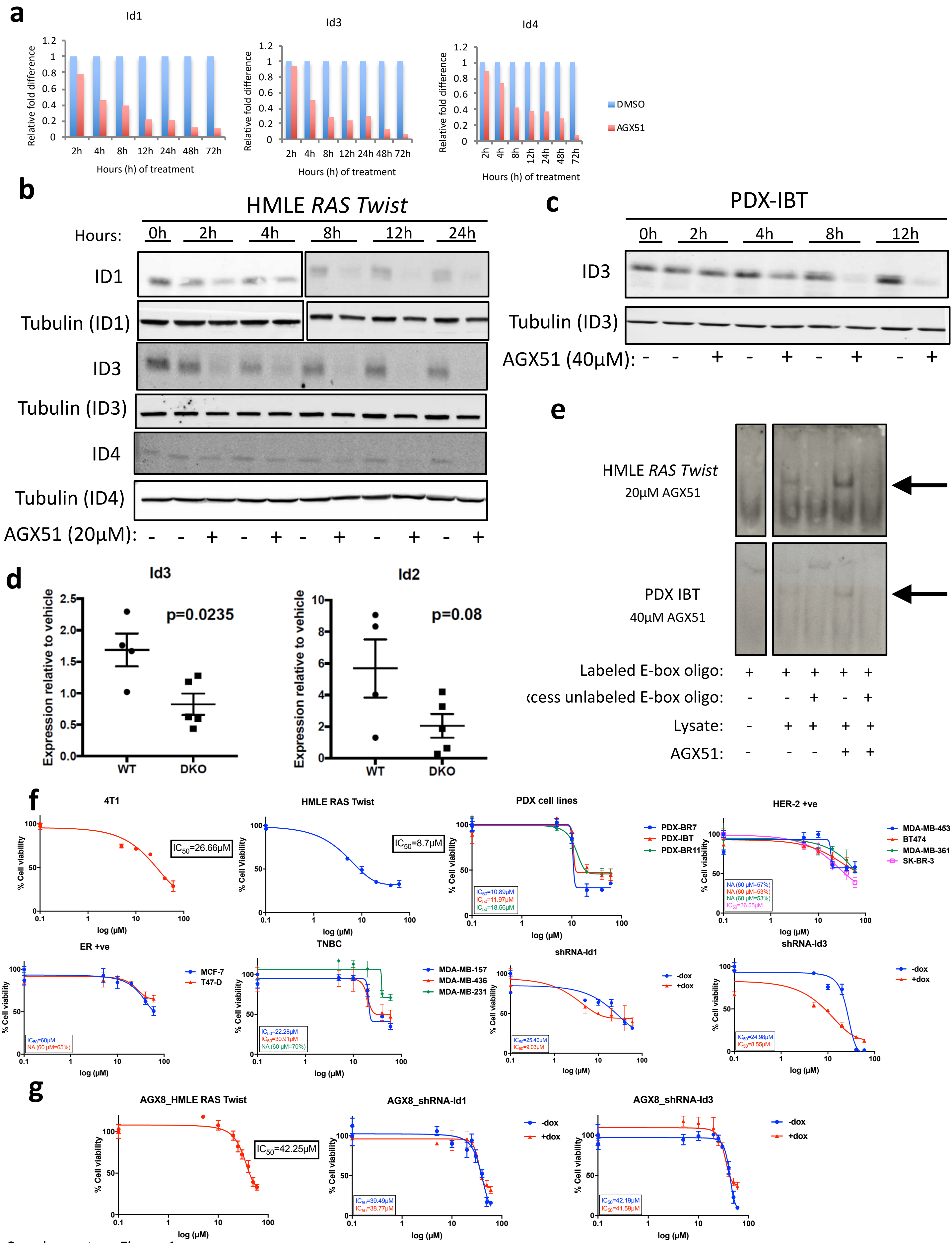

Supplementary Figure 1

Supplementary Figure 1. Protein expression and EMSAs in HMLE RAS Twist and PDX IBT cell lysates. Related to Figure 1. (A) Quantification of Western blot data presented in Figure 1b. (B) Western blot for ID1, ID3 and ID4 on whole cell lysates from HMLE RAS Twist cells treated with 20 μM AGX51 for 0-24 hours. (C) Western blot for ID3 on whole cell lysates from PDX IBT cells treated with 40 μM AGX51 for 0-12 hours. (D) mRNA levels of Id3 and Id2, as measured by qPCR, in E protein knockout (DKO) and wild-type (WT) T cells following treatment with AGX51 or vehicle for 24 hours. Each point is the normalized expression for technical triplicates of one biological replicate. The error bars are SEM. (E) EMSA on lysates from HMLE RAS Twist (upper blots) and PDX IBT (lower blots) cells treated with 20 or 40 μM AGX51, respectively, for 24 hours. Arrows indicate binding to DNA. Tubulin is used as a protein loading control. (F) Cell viability curves used to calculate IC50 values of cell lines treated with AGX51. Cells were treated in triplicate and error bars represent SD. (G) Cell viability curves used to calculate IC50 values of cell lines treated with AGX8. Cells were treated in triplicate and error bars represent SD.

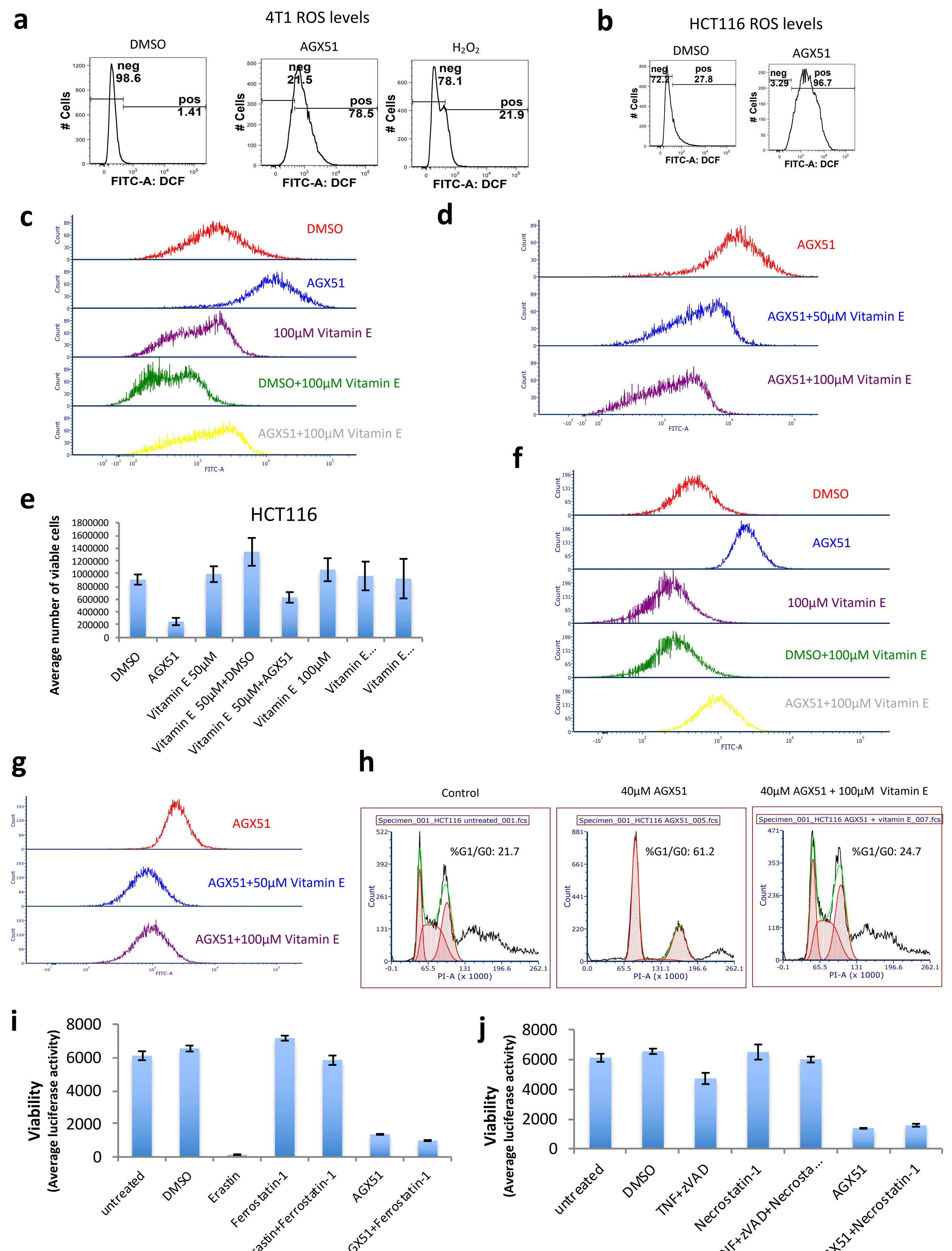

Supplementary Figure 2

Supplementary Figure 2. ROS levels in HCT116 and 4T1 cells following AGX51 treatment with and without vitamin E. Related to Figure 2. (A) Reactive oxygen species accumulation in 4T1 cells induced by 24 hour treatment with AGX51 (40 μM) or H2O2 (100 μM), as determined by H2DCFDA staining followed by flow cytometry analysis, with FITC positivity indicative of ROS presence. (B) Reactive oxygen species accumulation in HCT116 cells induced by AGX51 treatment (C) Reactive oxygen species levels in 4T1 cells treated with 40 μM AGX51 for 24 hours with or without 1 hour vitamin E pre- treatment (100 μM). (D) Reactive oxygen species levels in 4T1 cells treated with 40 μM AGX51 for 24 hours with or without 1 hour vitamin E pre-treatment (50 or100 μM). (E) Viable cell numbers, as determined by trypan blue exclusion and cell counting, of HCT116 cells pretreated with vitamin E (50 or 100 μM) for 1 hour followed by 24 hours of AGX51 (40 μM), or DMSO control. Five replicates were performed and error bars represent SEM. (F) Reactive oxygen species levels in HCT116 cells treated with 40 μM AGX51 for 24 hours with or without 1 hour vitamin E pre-treatment (100 μM). (G) Reactive oxygen species levels in HCT116 cells treated with 40 μM AGX51 for 24 hours with or without 1 hour vitamin E pre-treatment (50 or 100 μM). (H) Cell cycle analysis of HCT116 cells treated with 40 μM AGX51 for 24 hours, with or without 1 hour pretreatment with 100 μM vitamin E. (I) 4T1 cells were treated with vehicle, erastin (to induce ferroptosis), ferrostatin-1 (a ferroptosis inhibitor), AGX51 or combinations thereof and cell viability was determined via cell titer glo. Six replicates were performed and error bars represent SEM. (J) 4T1 cells were treated with vehicle, TNF +zVAD (to induce necroptosis), necrostatin-1 (a necroptosis inhibitor), AGX51 or combinations thereof and cell viability was determined via celltiter-glo which measures ATP as an indicator of metabolically active cells. Six replicates were performed and error bars represent SEM.

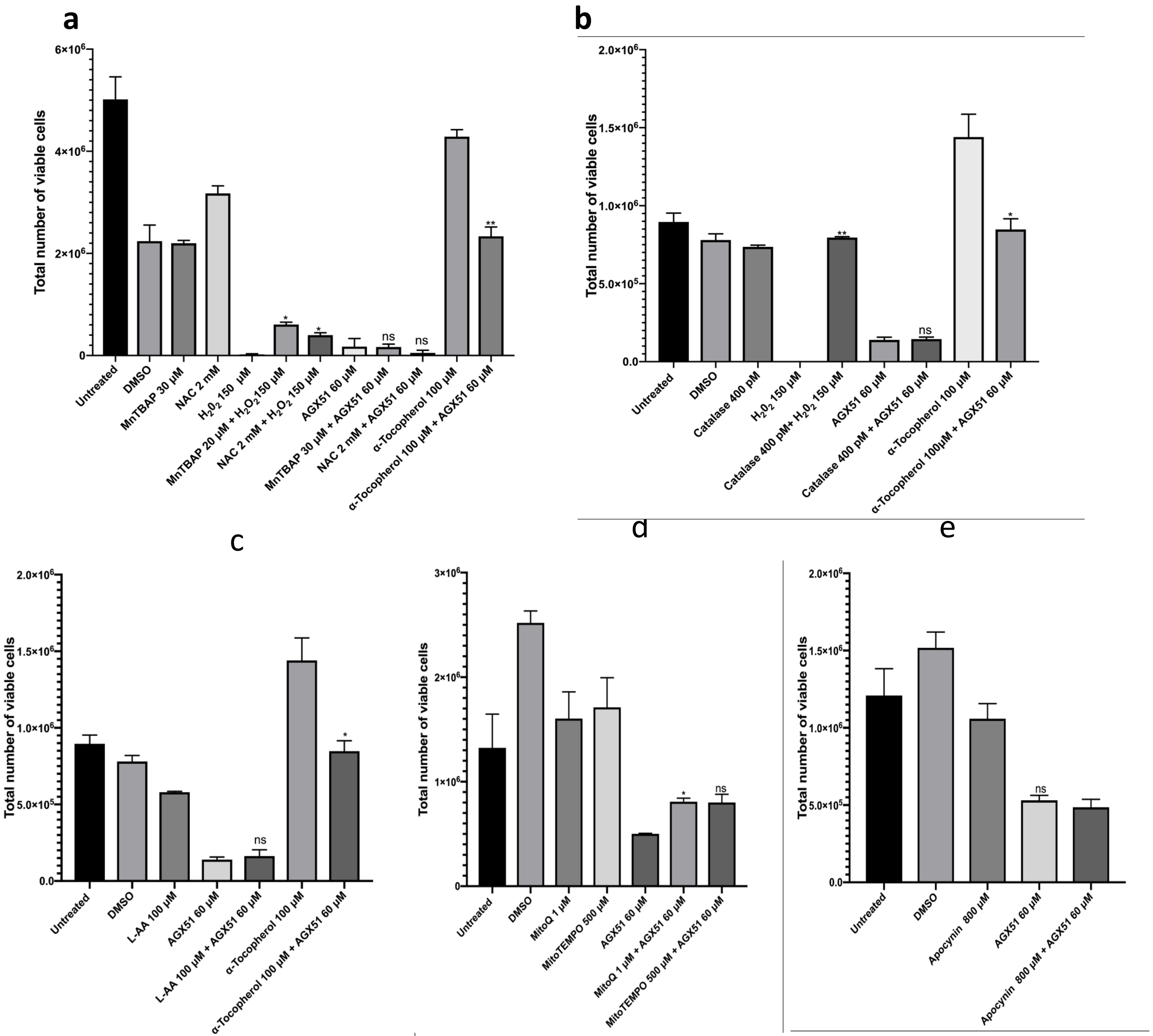

Supplementary Figure 3

Supplementary Figure 3. Catalase, MnTBAP or N-Acetyl-Cysteine (NAC) partially rescue cells from H2O2 oxidative stress but not AGX51. Related to Figure 2. All treatments were performed in duplicate and error bars are SD.
A) 4T1 cells in a 6-well dish were treated with 150 μM hydrogen peroxide or with 60 μM AGX51 in the presence of MnTBAP (30 μM) or N-Acetyl-Cysteine (2 mM). The cell viability was measured by tryptophan blue staining exclusion 48 hours after the treatment. -Tocopherol (100 μM) was used as a positive control, which rescues 4T1 cells from the AGX51-induced phenotype. B) 4T1 cells in a 6-well dish were treated with 150 μM hydrogen peroxide or with 60 μM AGX51 in the presence of Catalase (400 pM). Cell viability was measured with trypan blue staining exclusion 20 hours after treatment. Tocopherol (100 μM) was used as a positive control, which rescues 4T1 cells from the AGX51-induced phenotype. C,D,E) As in A,B with the use of L-ascorbic acid (L-AA), MitoQ, MitoTEMPO and apocynin. Statistical differences were represented $p > 0.05$ (ns), $p < 0.05$ (*), $p < 0.01$ (**). Statistical significance was assessed using unpaired Student’s t-TEST with Welch's correction.

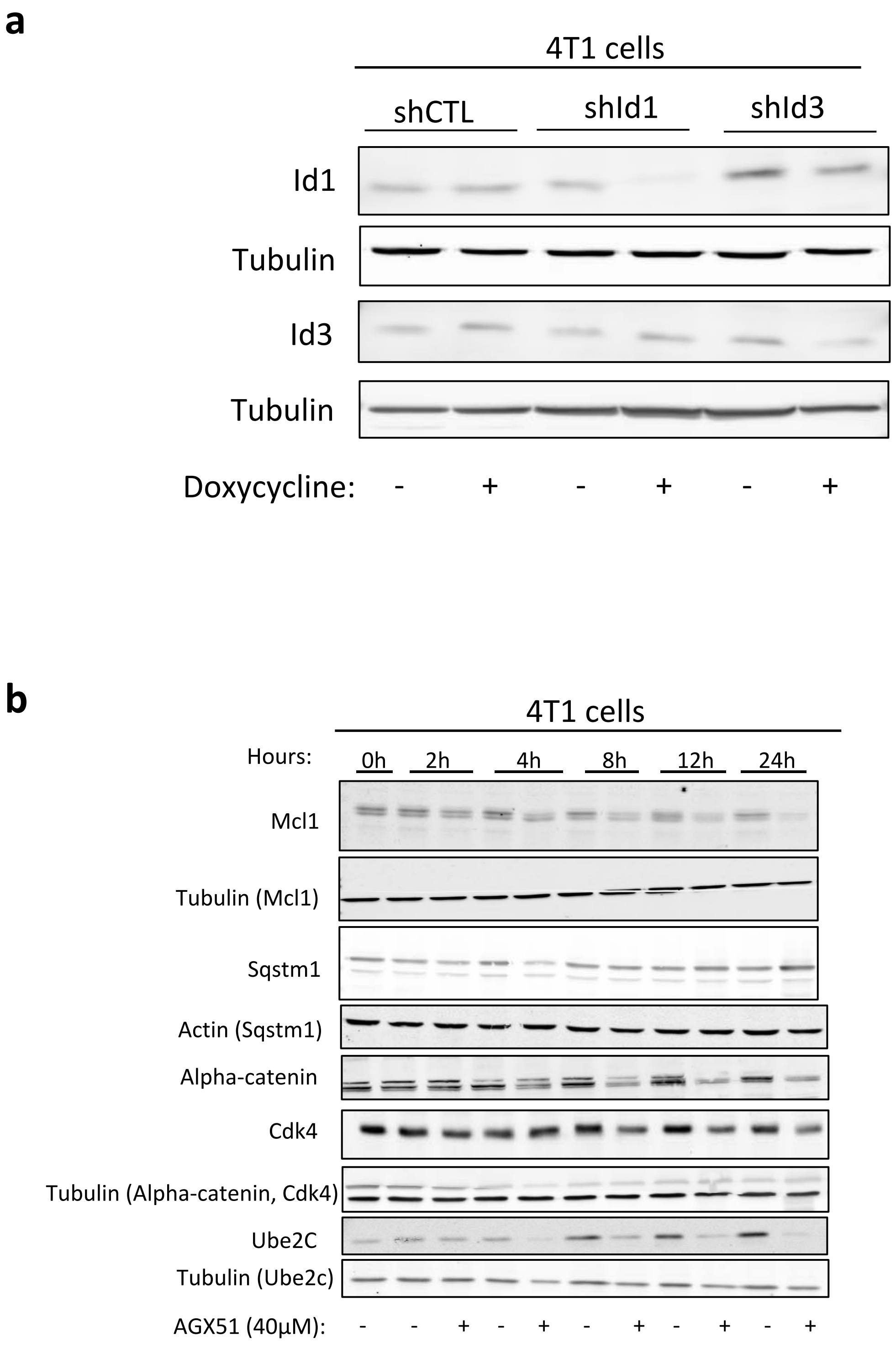

Supplementary Figure 4. Protein expression in 4T1 cells. Related to Figure 4. (A) Western blot for ID1 and ID3 on whole cell lysates from 4T1 cells transduced with doxycycline-inducible constructs expression short hairpins against Renilla (shCTL), Id1 (shId1) or Id3 (shId3) with or without doxycycline treatment. (B) Western blot for Mcl1, Sqstm1, Alpha-catenin, Cdk4, and Ube2C on whole cell lysates from 4T1 cells treated with 40 µM AGX51 for 0-24 hours.

a

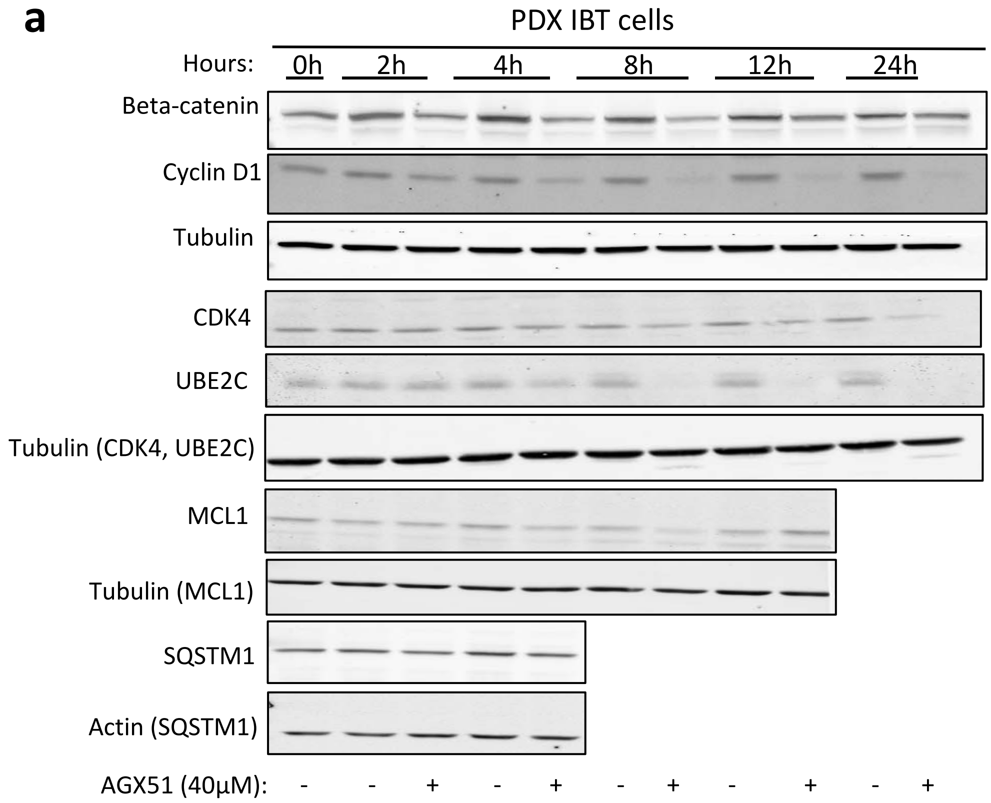

b

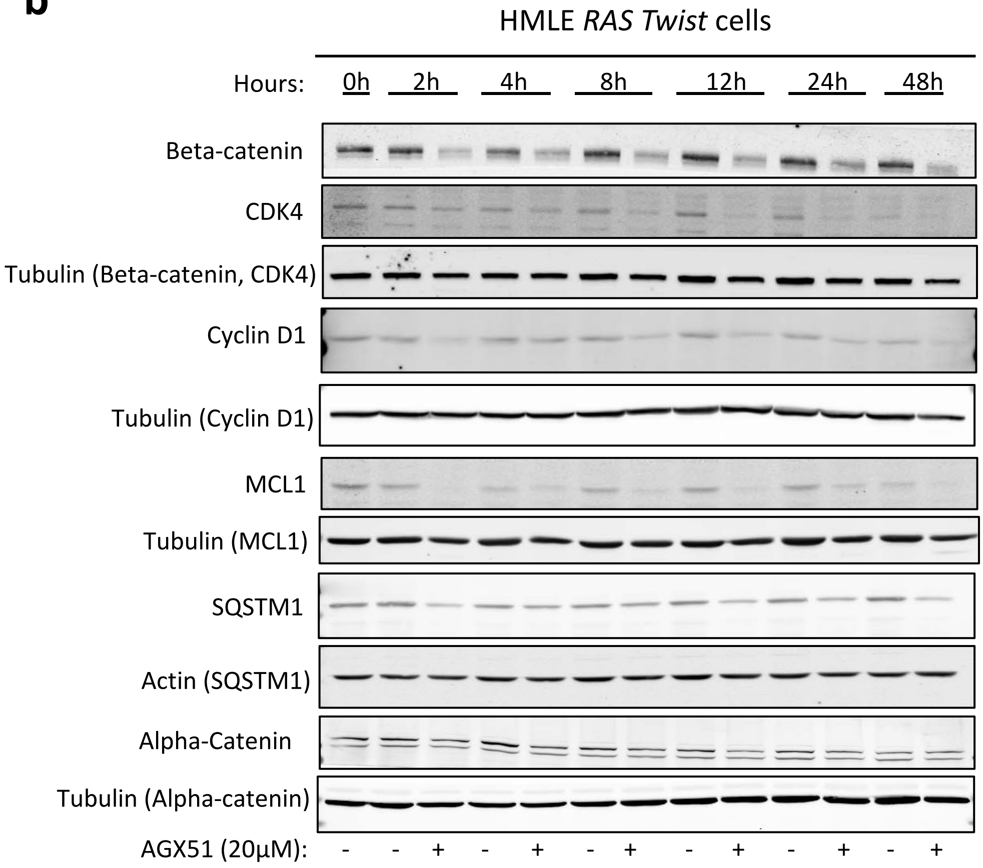

Supplementary Figure 5

Supplementary Figure 5. Protein expression in PDX IBT and HMLE RAS Twist cells. Related to Figure 4. (A) Western blot for Beta-catenin, Cyclin D1, CDK4, UBE2C, MCL1 and SQSTM1 on whole cell lysates from PDX IBT cells treated with 40 μM AGX51 for 0-24 hours. Tubulin/Actin are used as protein loading controls. (B) Western blot for Beta-catenin, Cyclin D1, CDK4, SQSTM1, MCL1 and Alpha-catenin on whole cell lysates from HMLE RAS Twist cells treated with 20 μM AGX51 for 0-48 hours.

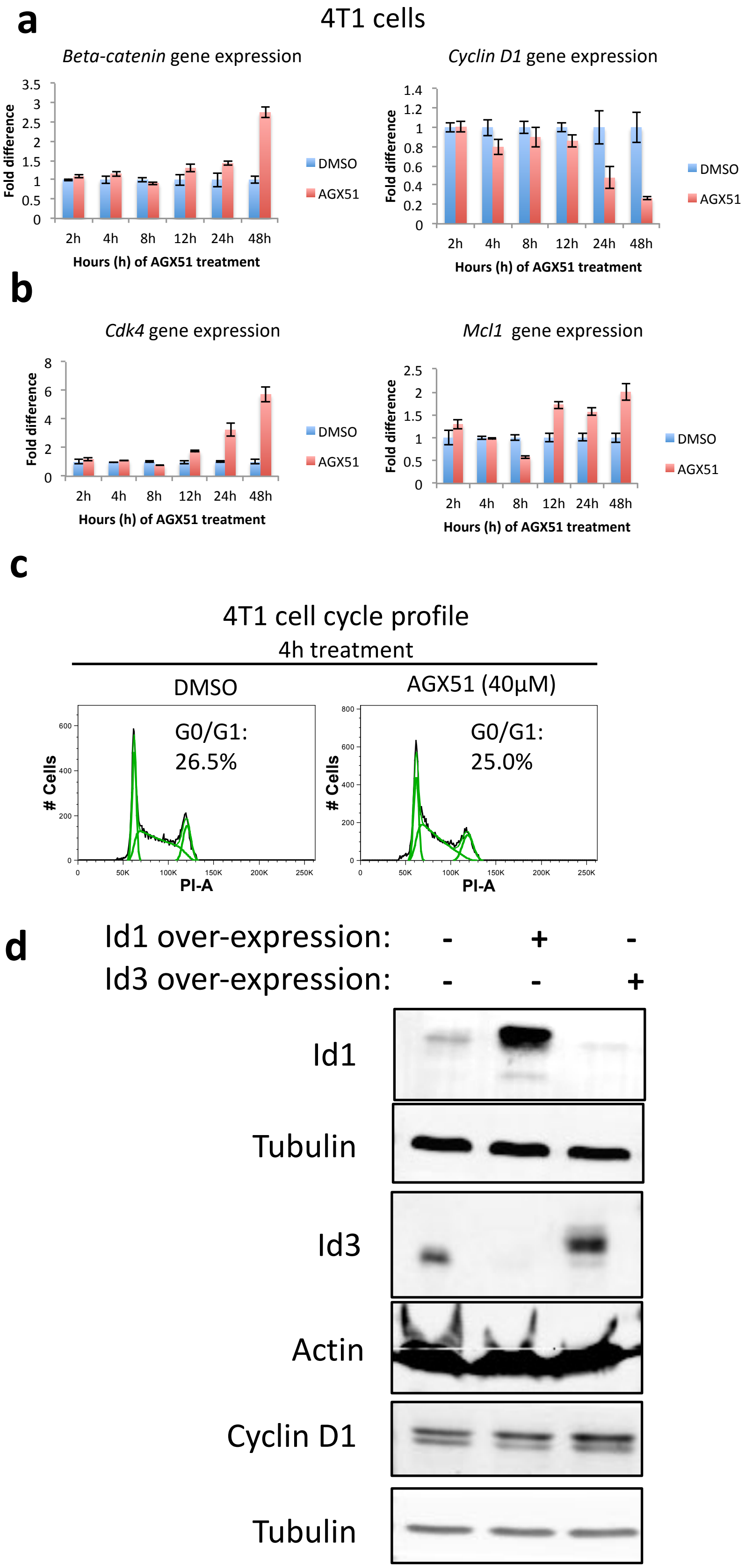

Supplementary Figure 6

Supplementary Figure 6. Gene expression analysis of genes found differentially expressed in SILAC analysis of AGX51-treated 4T1 cells. Related to Figure 4. (A) qRT-PCR analysis for relative Beta-catenin and Cyclin D1 mRNA levels in 4T1 cells treated with 40 μM AGX51 for 2-48 hours. Data shows mean of technical triplicates with error bars representing SEM. (B) qRT-PCR analysis for Cdk4 and Mcl1 expression in 4T1 cells treated with 40 μM AGX51 for 2-48 hours. Data shows mean of technical triplicates with error bars representing SEM. (C) Cell cycle analysis of 4T1 cells treated with 40 μM AGX51 for four hours. (D) Western blot for ID1, ID3 and Cyclin D1 on whole cell lysates from 4T1 cells overexpressing either ID1 or ID3 (as indicated). Tubulin/Actin are used as protein loading controls.

4T1 cells

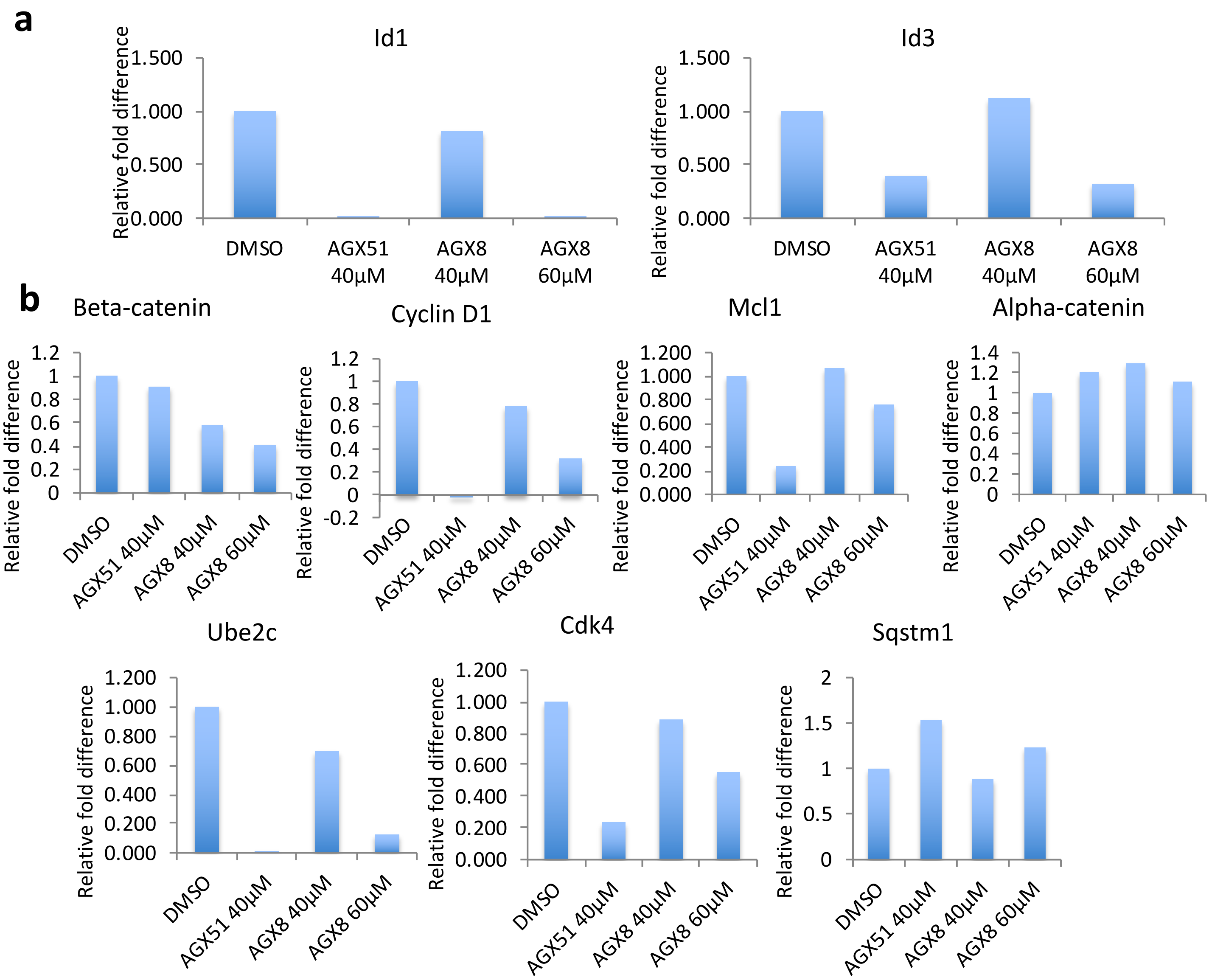

Supplementary Figure 7. Western blot quantification. Related to Figure 4. (A and B) Western blot quantification for Western blots presented in Figure 4G and H, respectively.

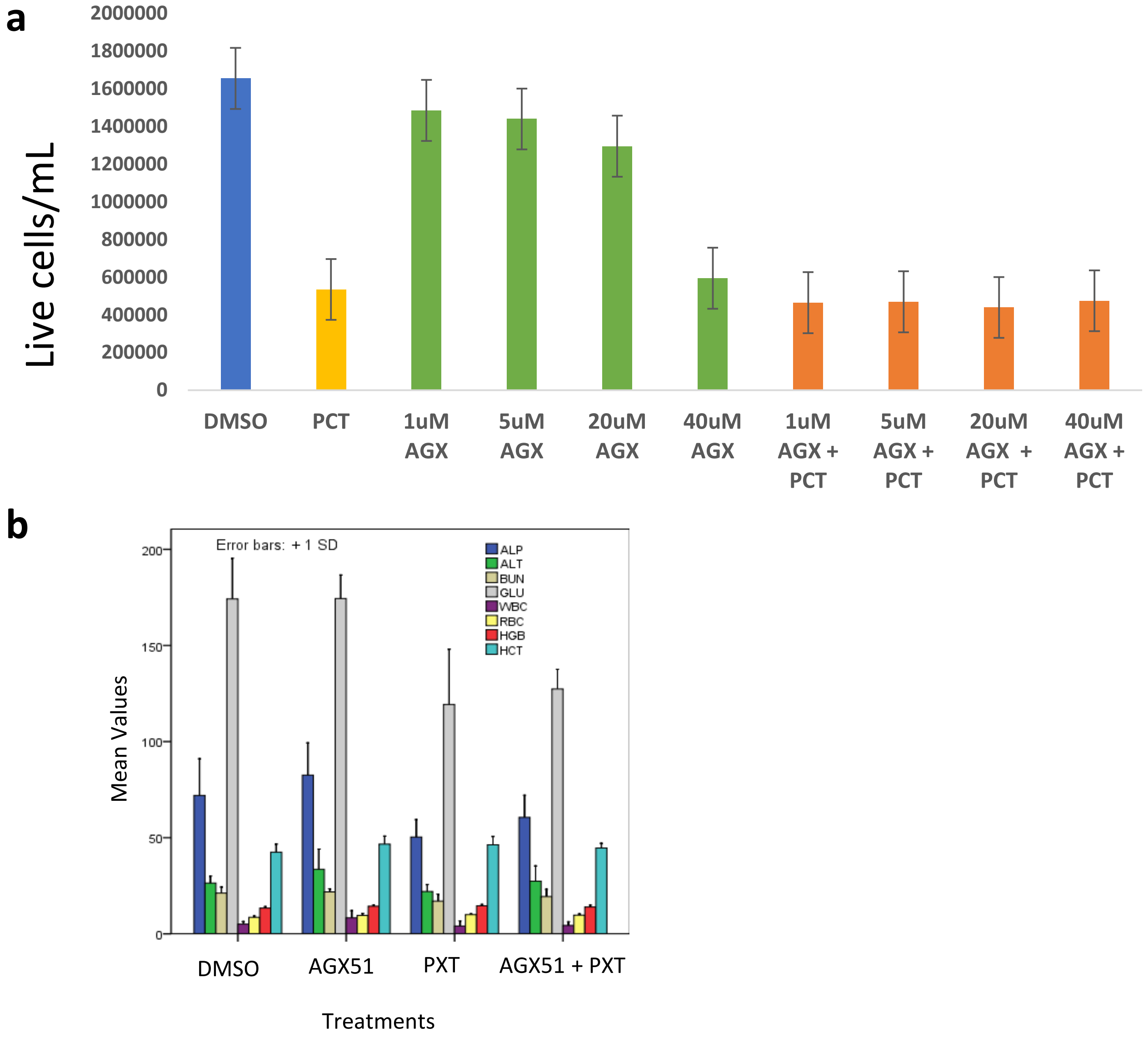

Supplementary Figure 8. Paclitaxel and AGX51 in vitro synergy and in vivo toxicity analysis of AGX51. Related to Figure 5. (A) MDA-MB-231 cells were treated with AGX51 (at indicated doses) and 20 nM paclitaxel (PCT) for 24 hours and cell number was determined by counting trypan blue excluding cells. The cells were treated in duplicate and error bars represent SEM. (B) Systemic clinical chemistry and hematology concentrations were determined in mice treated for 14 days with vehicle, AGX51 (60 mg per kg, qd), paclitaxel (15 mg per kg for 5 days), or paclitaxel plus AGX51 (n=5 mice per group). Mean values (with error bars showing SD) for ALP, alkaline phosphatase; ALT, alanine aminotransferase; BUN, blood urea nitrogen; GLU, glucose; WBC, white blood cell count; RBC, red blood cell count; HGB, hemoglobin; HCT, hematocrit are shown. Compared to vehicle, no effect of the AGX51 treatment on clinical chemistry or hematology values was observed. Similarly, values from the paclitaxel group and the AGX51+paclitaxel were statistically similar.

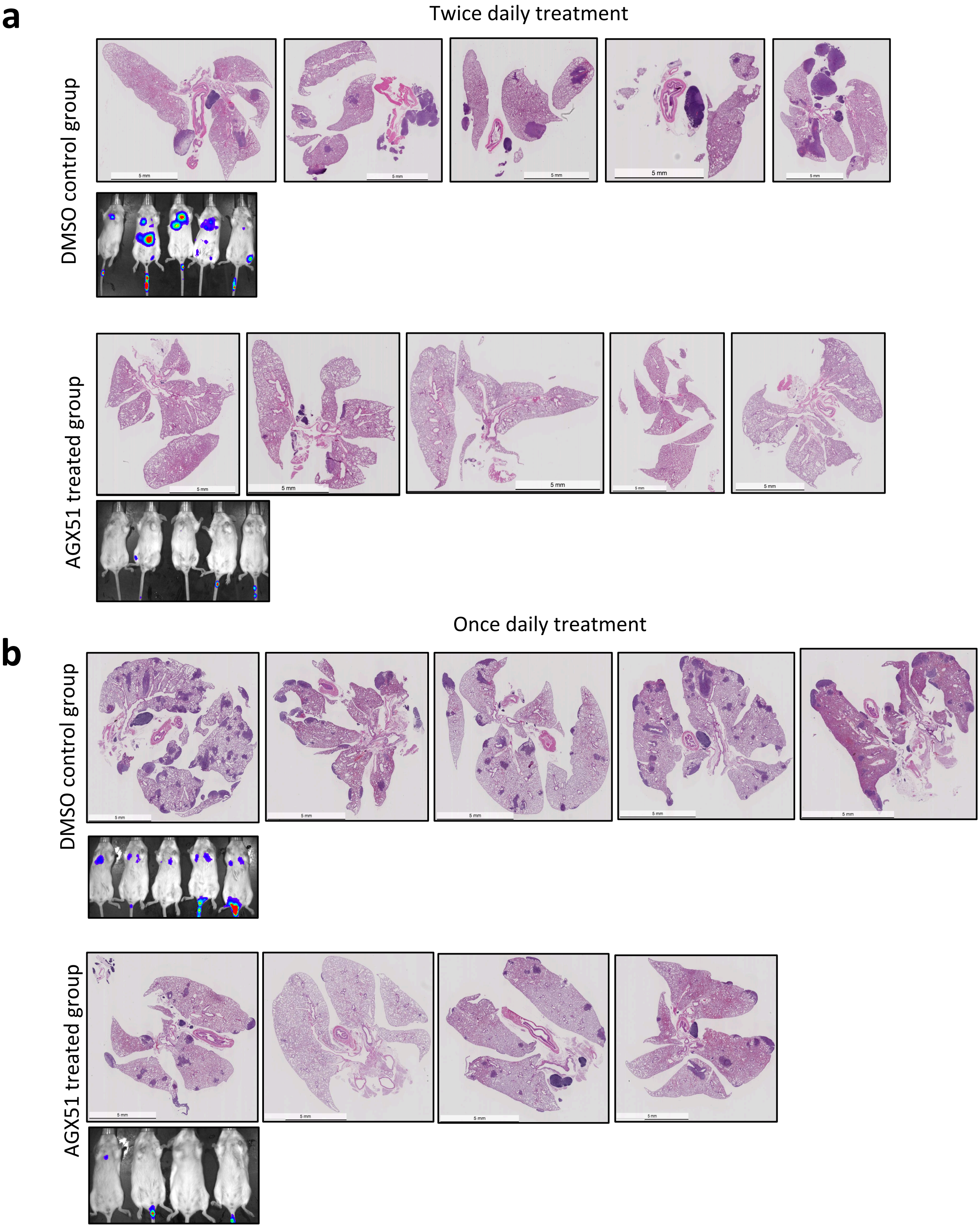

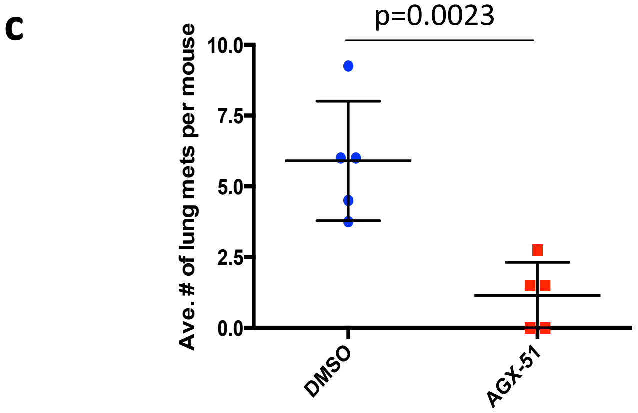

Supplementary Figure 9

Supplementary Figure 9. Lung metastases in AGX51 treated mice. Related to Figure 5. (A) H&E staining, and corresponding bioluminescent imaging from mice injected with 5x104 4T1 cells via tail vein and treated with 50 mg per kg bid or vehicle (n=5 mice per treatment group). (B) H&E staining, and corresponding bioluminescent imaging from mice injected with 5x106 4T1 cells via tail vein and treated with 50 mg per kg qd or vehicle (n=5 mice per treatment group, where one mouse died in the AGX51 treatment group following isoflurane anesthesia during the first bioluminescent imaging session). (C) Average number of lung metastases in mice injected with 5x104 4T1 cells via tail vein, treated with 50 mg per kg AGX51 qd or vehicle (n=5 or n=5, respectively). Means are plotted with error bars showing SEM. AGX51 treatment had a significant effect on lung tumor development ($p<0.0023$).

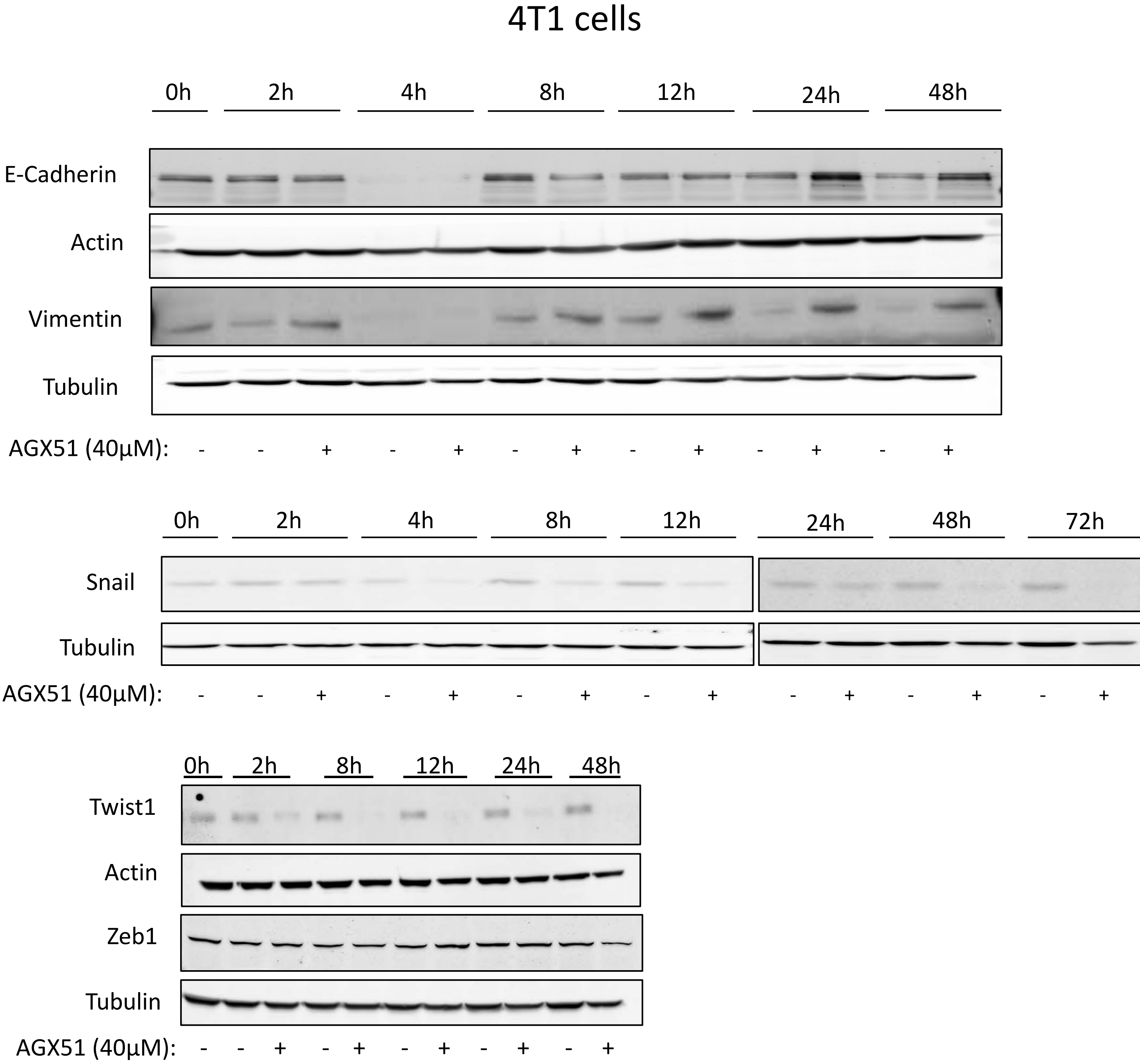

Supplementary Figure 10. Effects of AGX51 on EMT signatures. Related to Figure 4. (A) Western blot for E-cadherin, Vimentin, Snail, Twist1 and Zeb1 on whole cell lysates from 4T1 cells treated with 40 µM AGX51 for 0-48, or 0-72 hours. Tubulin/Actin serve as protein loading controls.

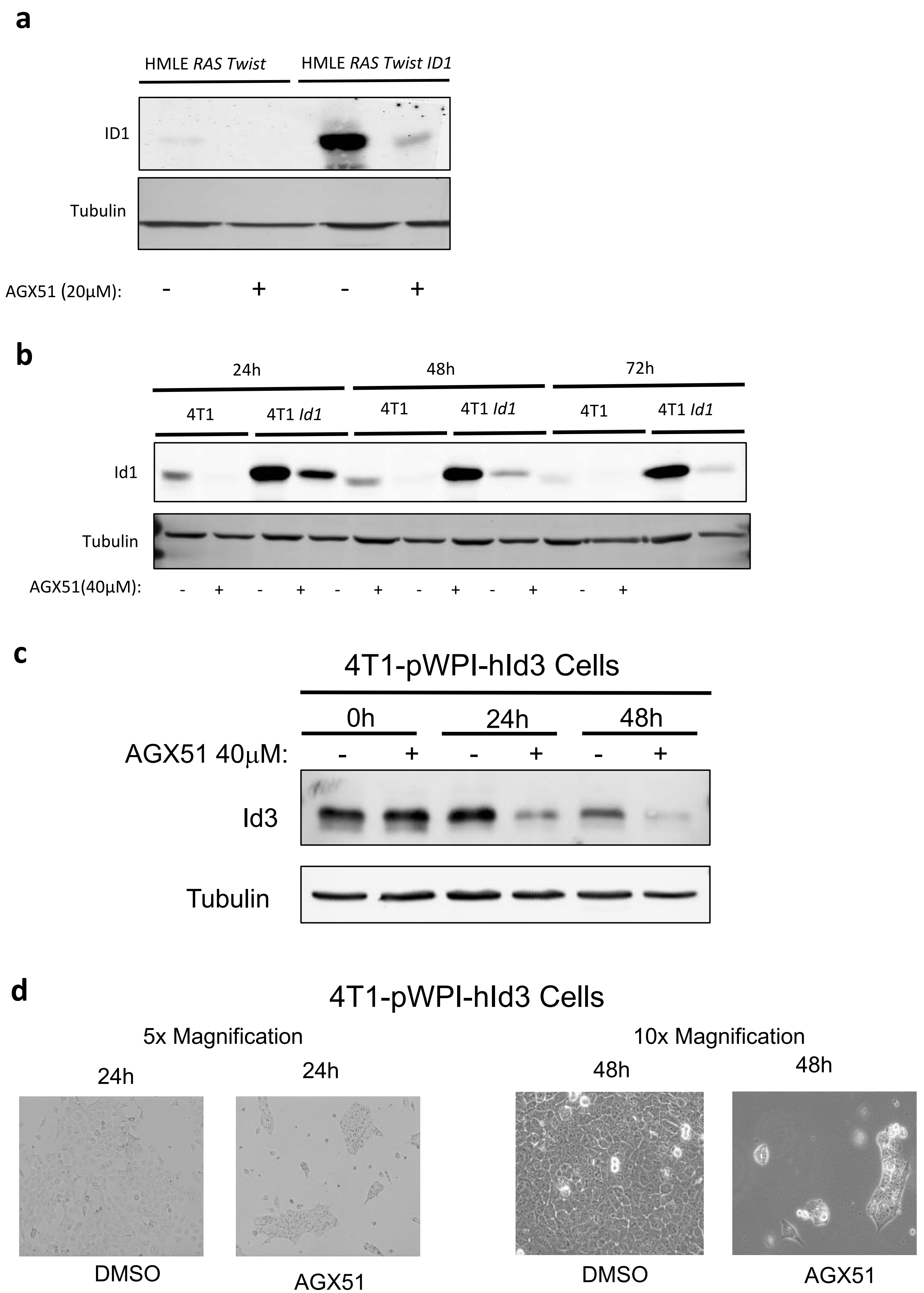

Supplementary Figure 11

Supplementary Figure 11. Effects of AGX51 on ID1-overexpressing cells. Related to Figure 6. (A) Western blot for ID1 on whole cell lysates from HMLE RAS Twist and HMLE RAS Twist ID1 cells treated with 20 μM AGX51 for 24 hours. (B) Western blot for ID1 on whole cell lysates from 4T1 and 4T1 Id1 cells treated with 40 μM AGX51 for 24, 48 and 72 hours. (C) Western blot for ID3 on whole cell lysates from 4T1-pWPI-hId3 cells treated with 40 μM AGX51 for 0, 24 and 48 hours. Tubulin shown for loading control. (D) Bright field images of 4T1-pWPI-hId3 cells treated with 40 μM AGX51 for 24 and 48 hours.

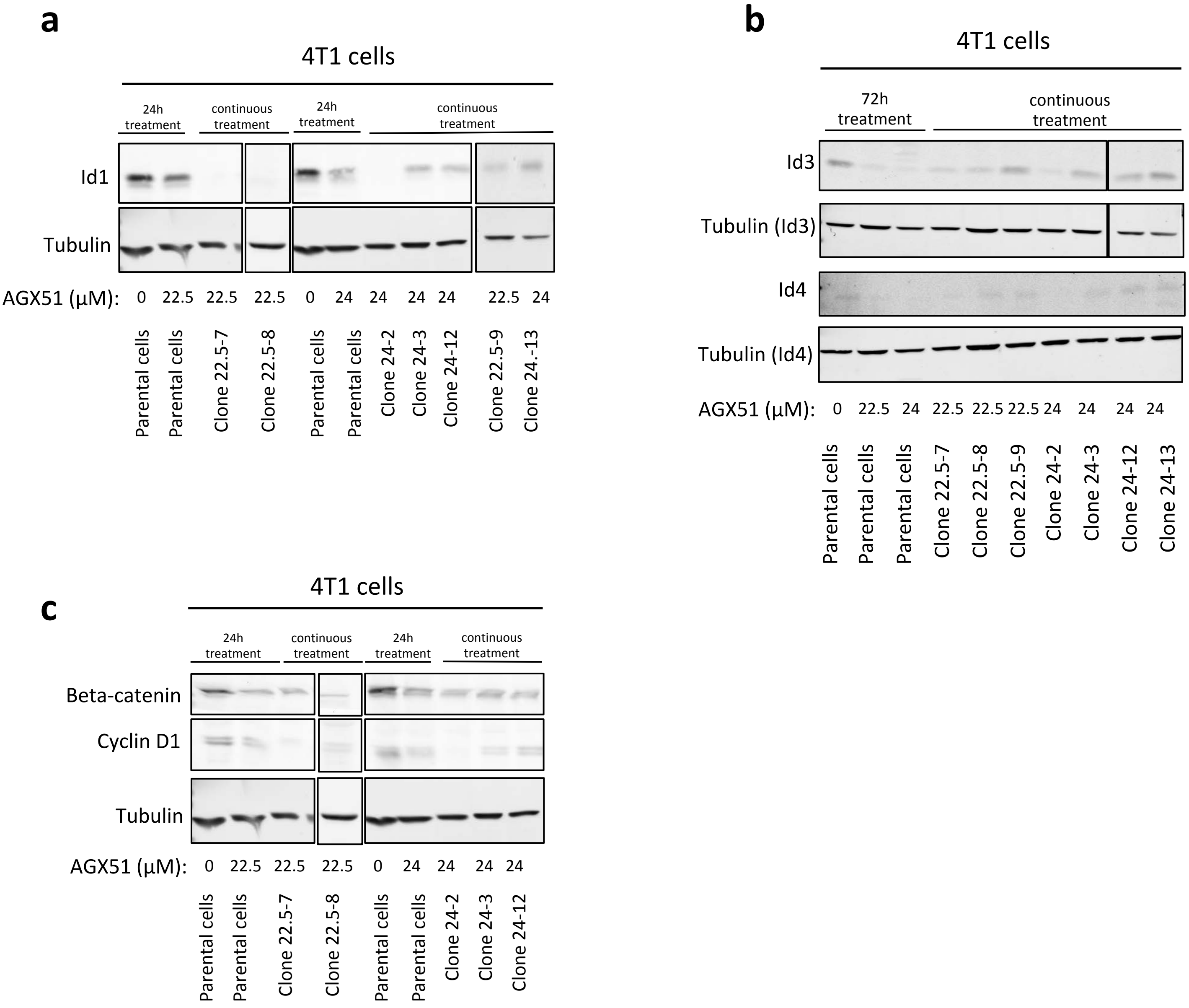

Supplementary Figure 12. Beta-catenin and Cyclin D1 expression in AGX51 transiently resistant clones. Related to Figure 6. (A) Western blot for ID1 on parental 4T1cells (treated for 24 hours) and transiently resistant clones treated (continuously) with 22.5 or 24 µM AGX51. (B) Western blot for ID3 and ID4 on parental 4T1 cells (treated for 72 hours) and transiently resistant clones treated (continuously) with 22.5 or 24 µM AGX51. (C) Western blot analysis for Beta-catenin and Cyclin D1 on cell lysates from parental 4T1 cells or transiently resistant clones treated with 22.5 or 24 µM AGX51 for 24 hours or continuously, as indicated.

Supplementary Figure 13. Uncropped images of Western blots/EMSAs show in figures

Figure 1a

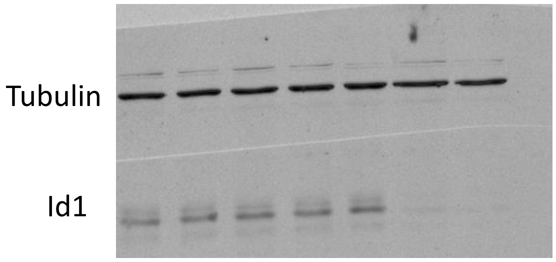

Figure 1b

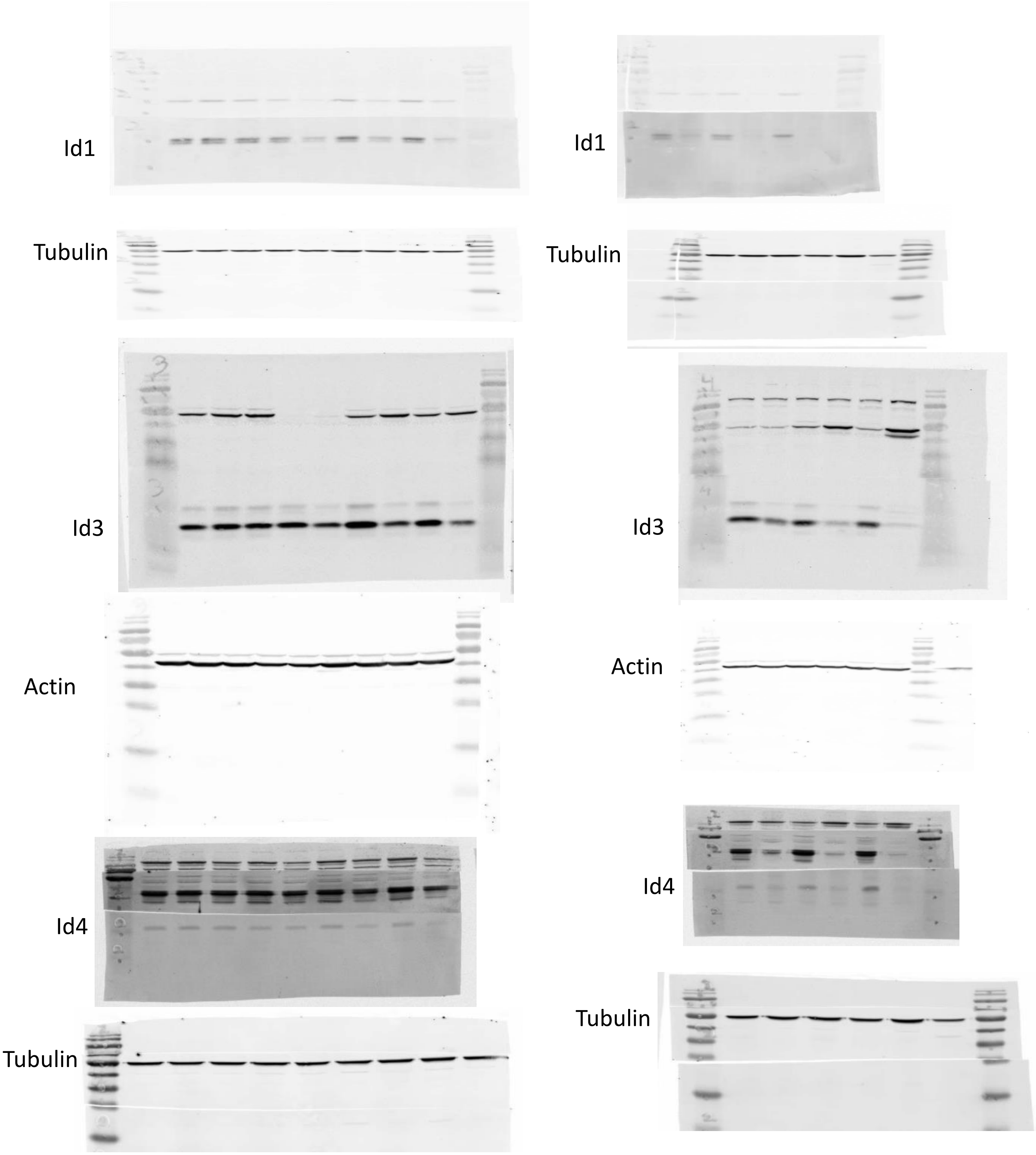

Figure 1c

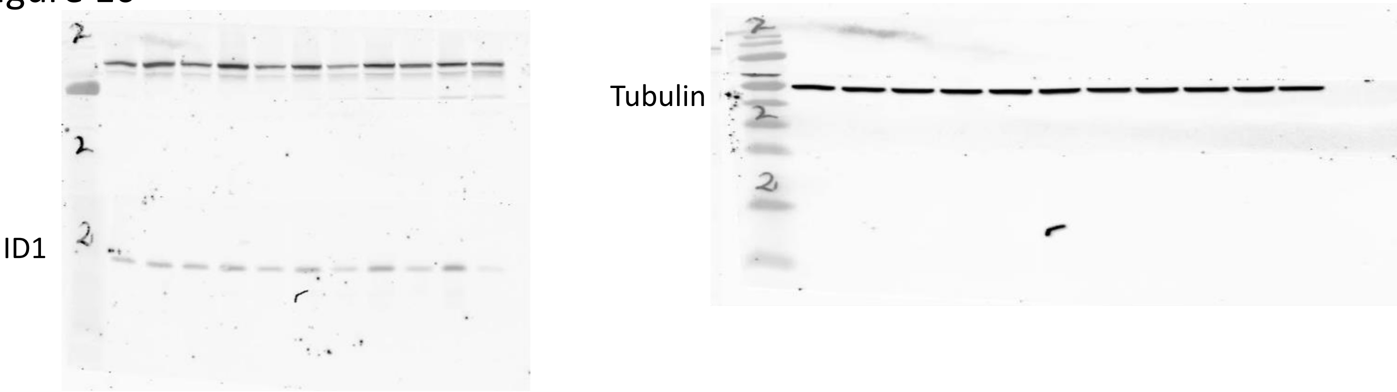

Figure 1d

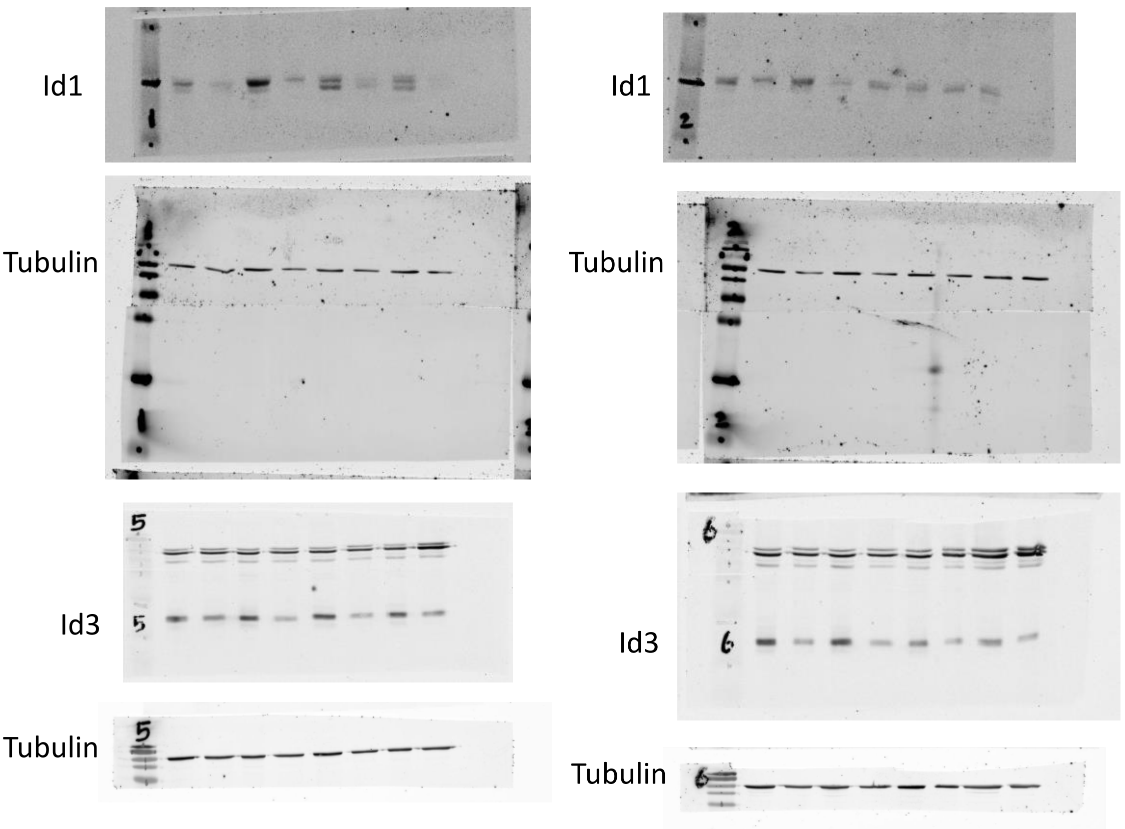

Figure 2d

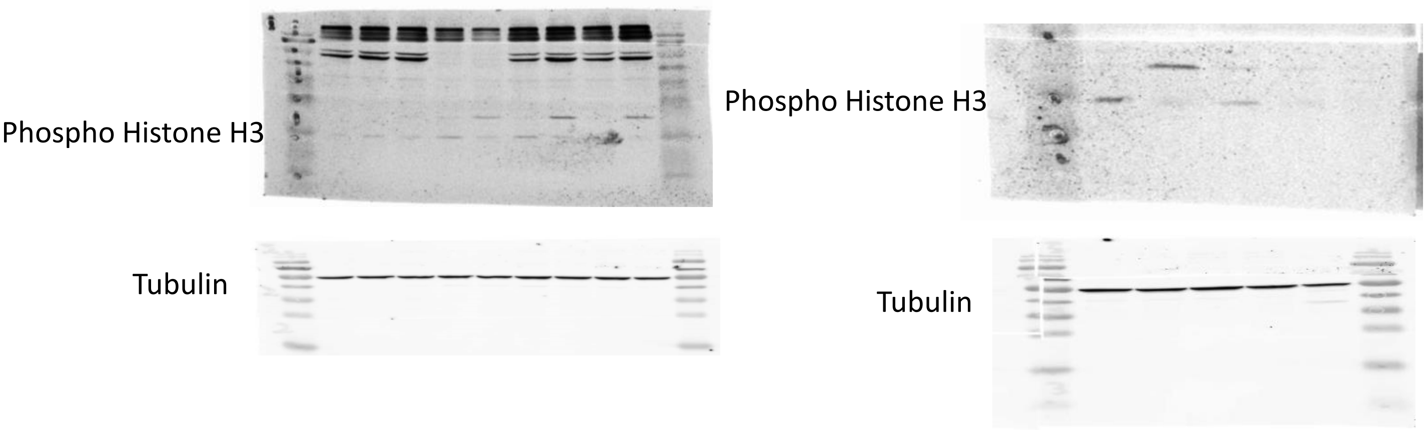

Figure 4b

Id1

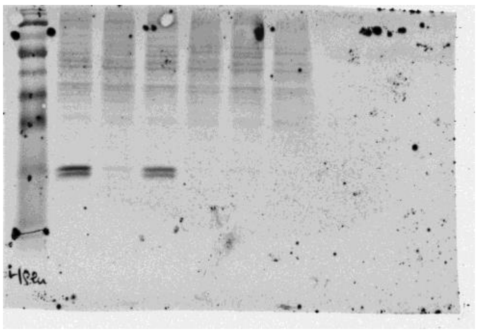

Id3

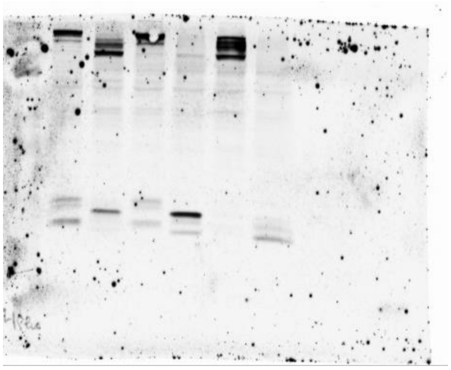

Actin

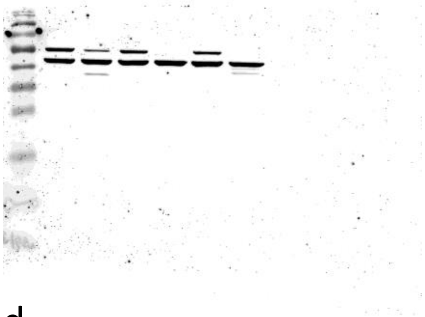

Figure 4d

Beta-catenin

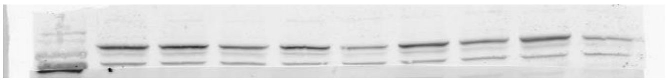

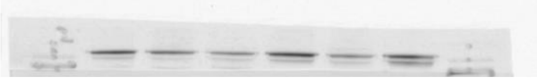

Cyclin D1

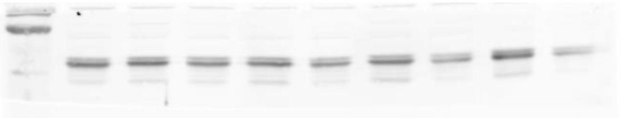

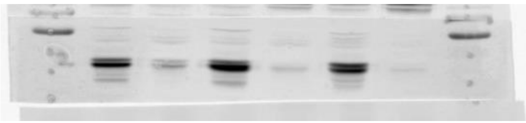

Tubulin

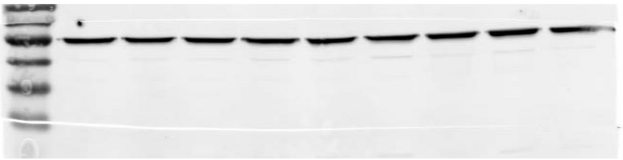

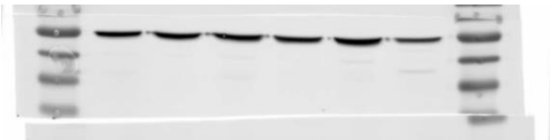

Figure 4g

Id1

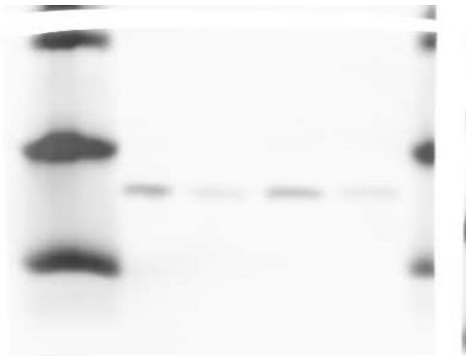

Id3

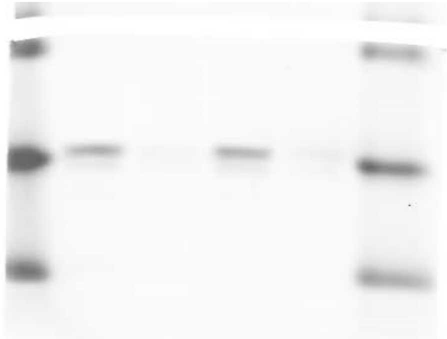

Tubulin

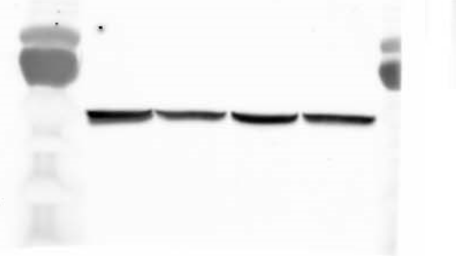

Tubulin

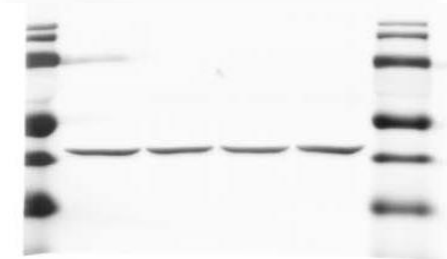

Figure 4h

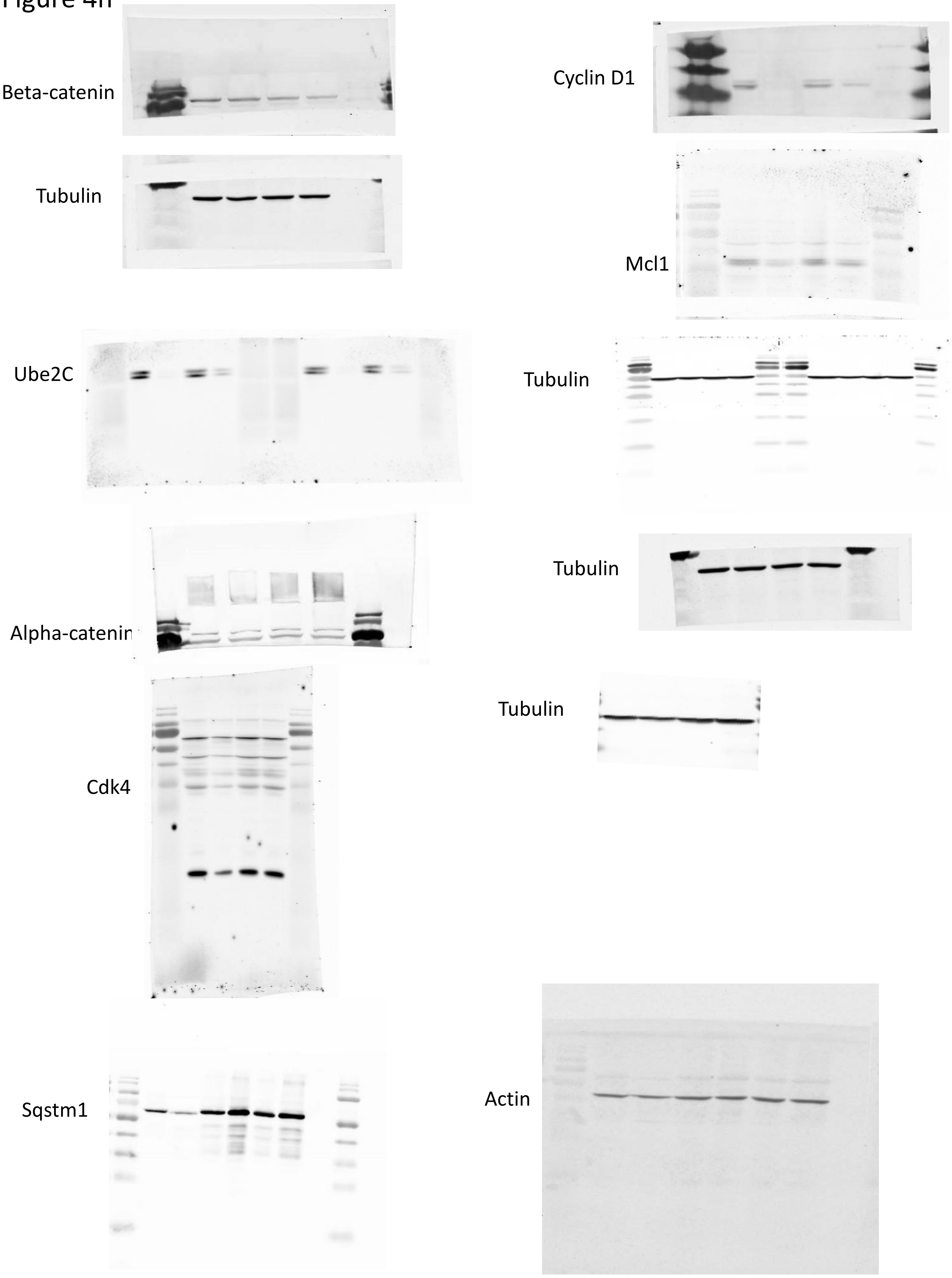
Beta-catenin
Tubulin
Cyclin D1
Mcl1
Ube2C
Tubulin
Tubulin
Alpha-catenin
Tubulin
Cdk4
Sqstm1
Actin

Figure 6a

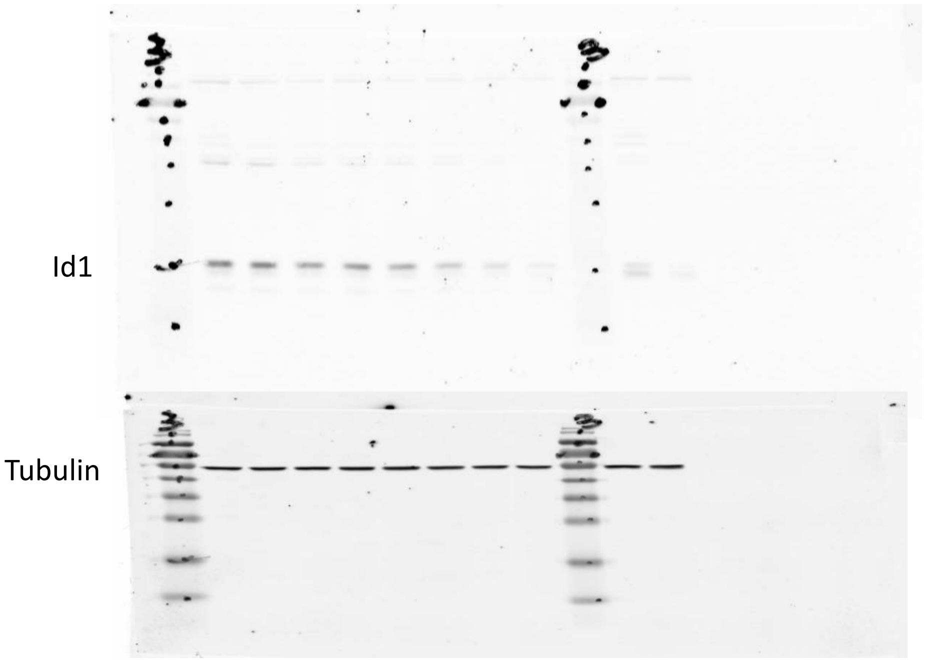

Figure 6c

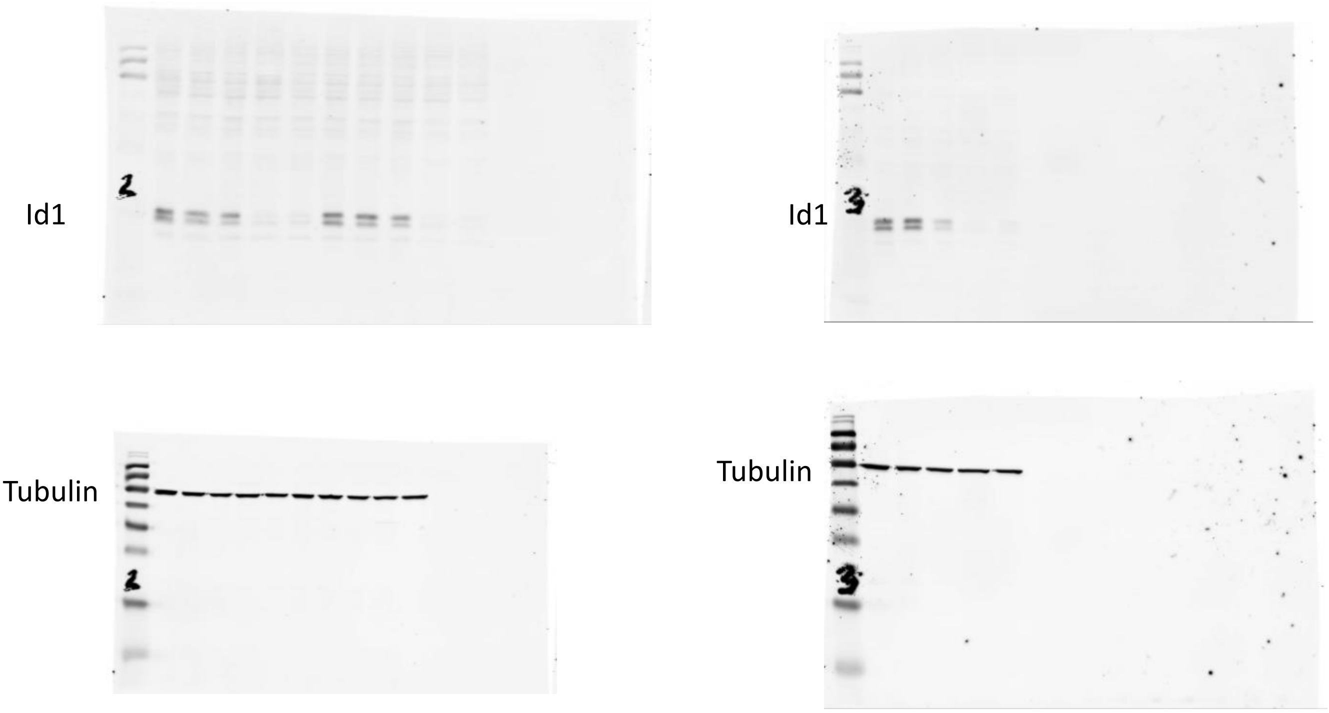

Supplementary Figure 1b

ID1

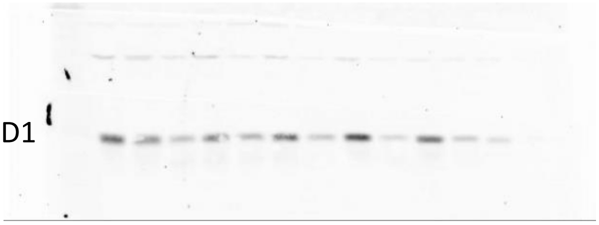

ID1

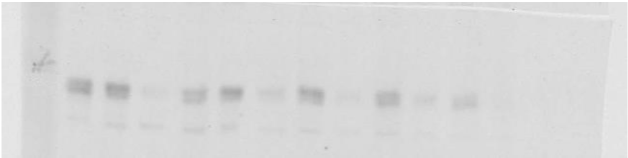

Tubulin

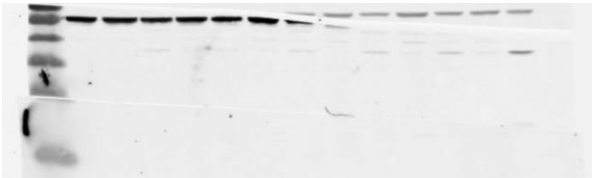

Tubulin

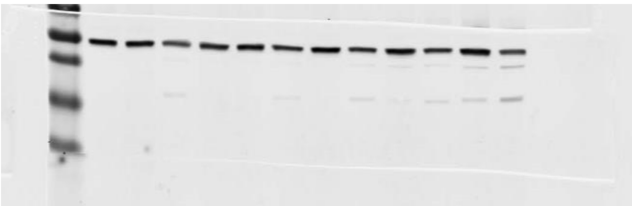

ID3

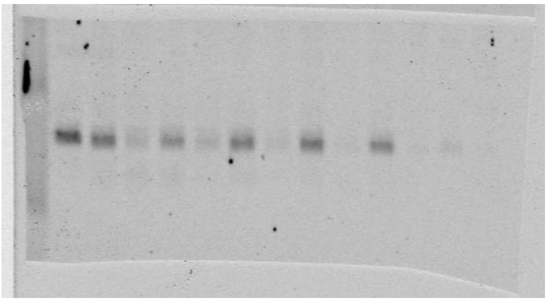

Tubulin

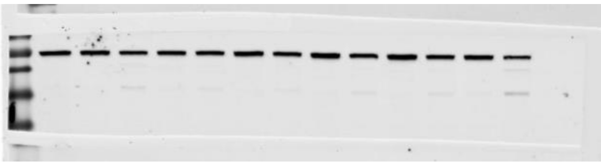

ID4

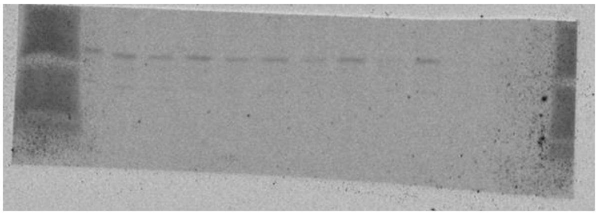

Tubulin

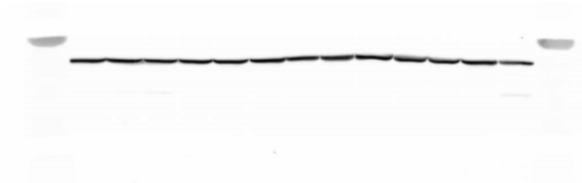

Supplementary Figure 1c

ID3

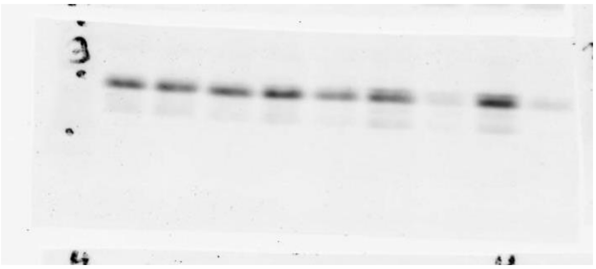

Tubulin

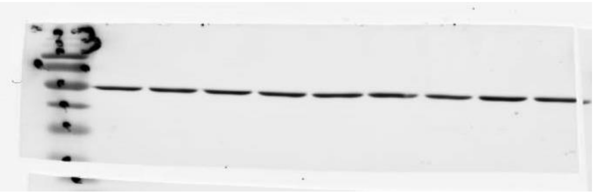

Supplementary Figure 4a

Id1

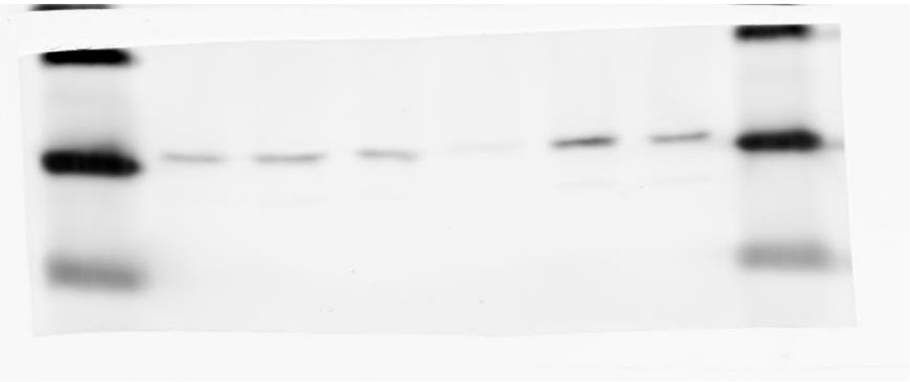

Tubulin

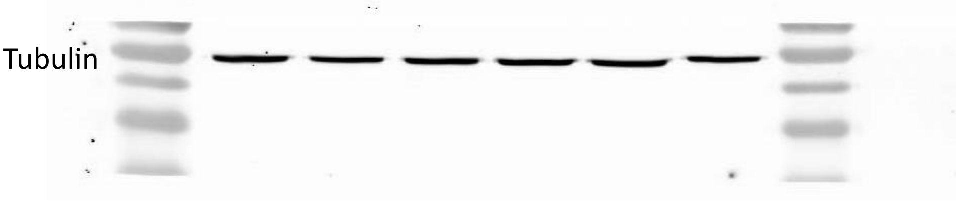

Id3

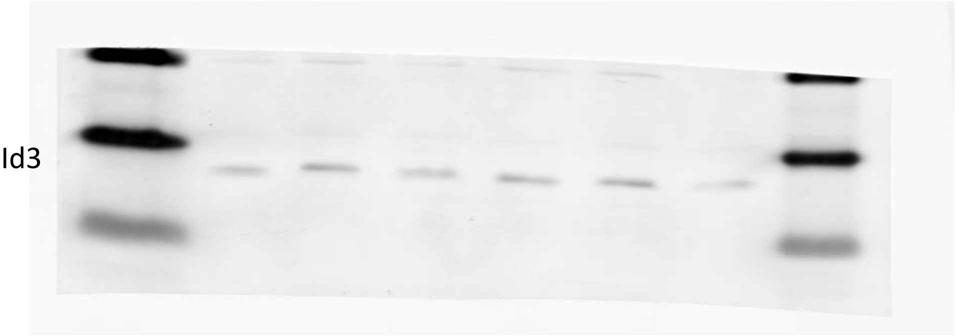

Tubulin

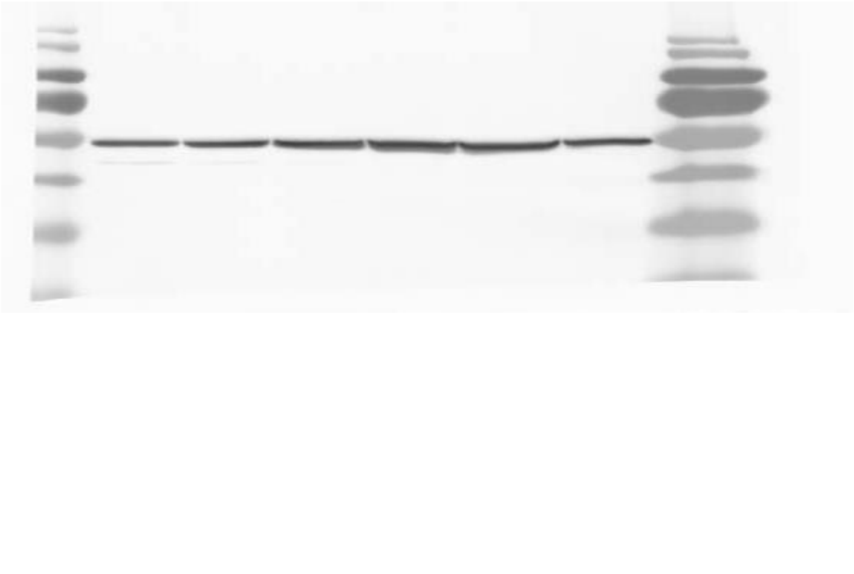

Supplementary Figure 4b

MCL1

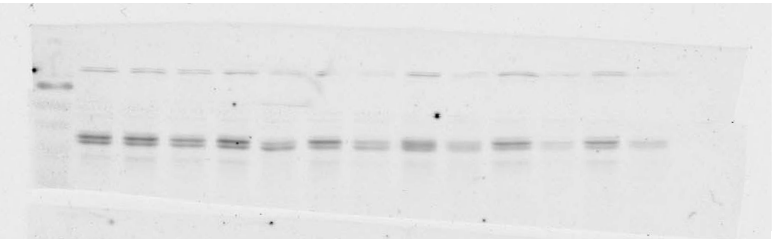

Tubulin

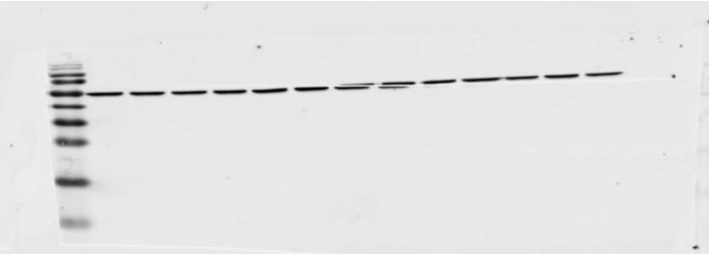

Sqstm1

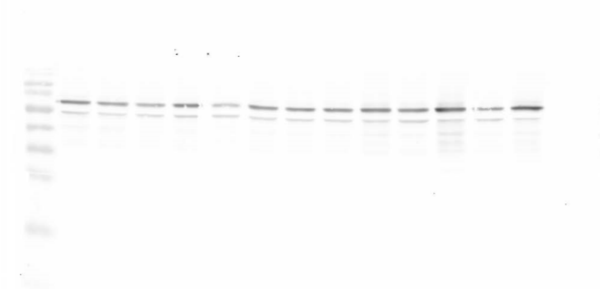

Actin

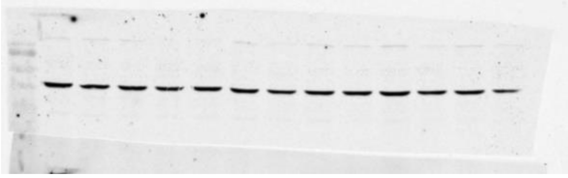

Alpha-catenin

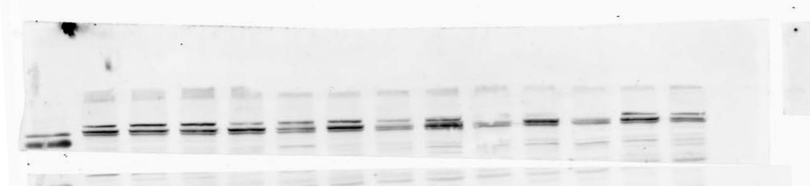

Cdk4

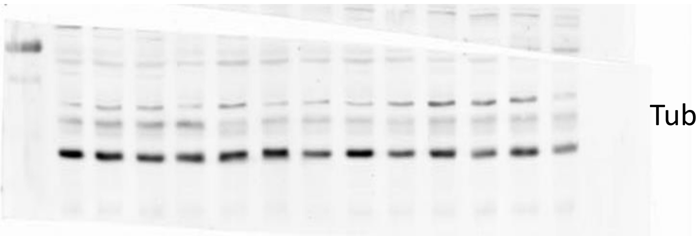

Tubulin

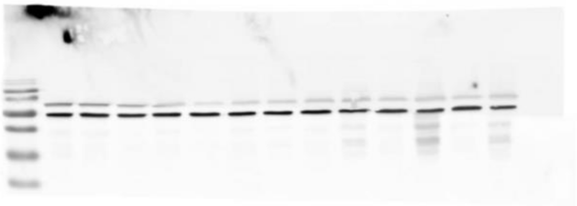

Ube2C

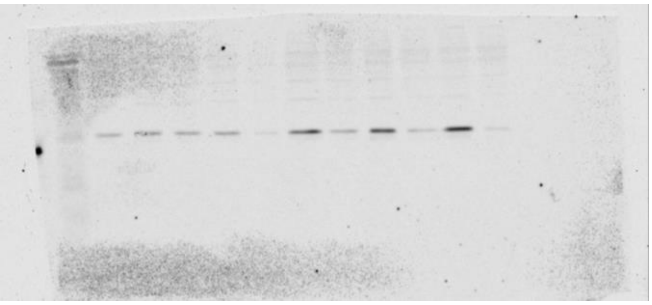

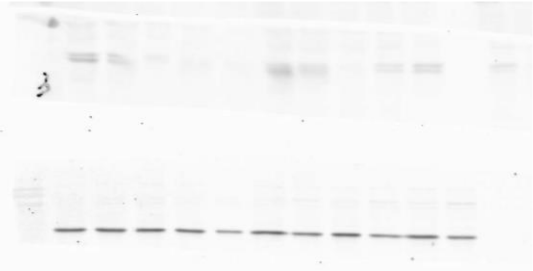

Tubulin

Supplementary Figure 5a

Beta-catenin

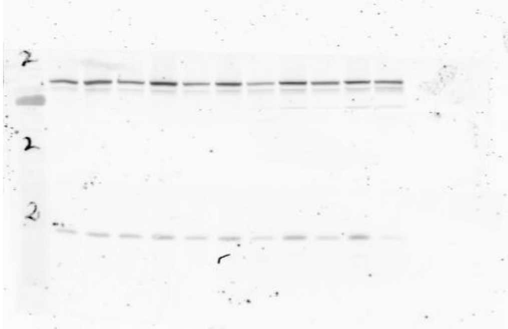

Cyclin D1

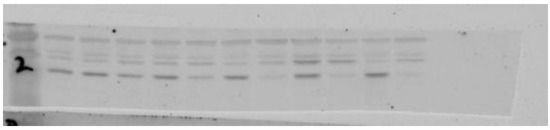

Tubulin

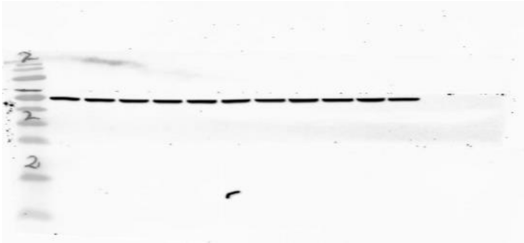

CDK4

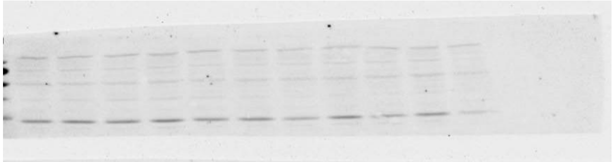

UBE2C

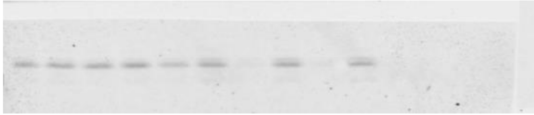

Tubulin

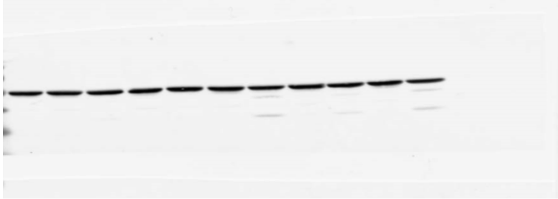

MCL1

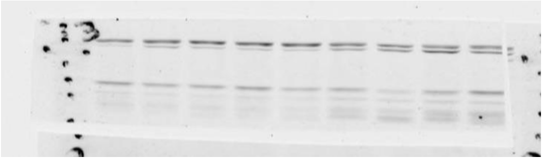

Tubulin

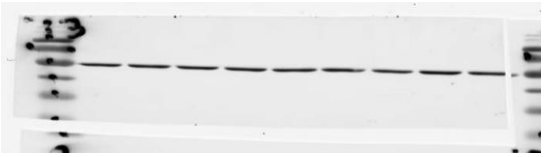

SQSTM1

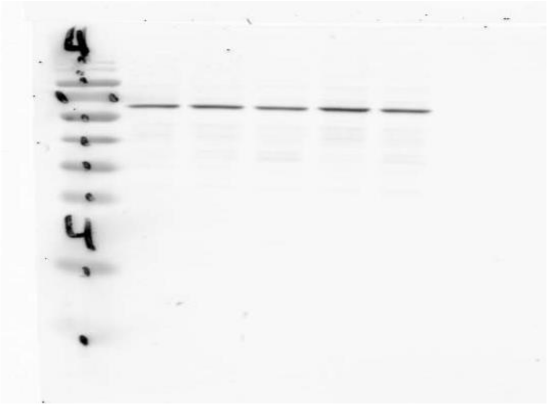

Actin

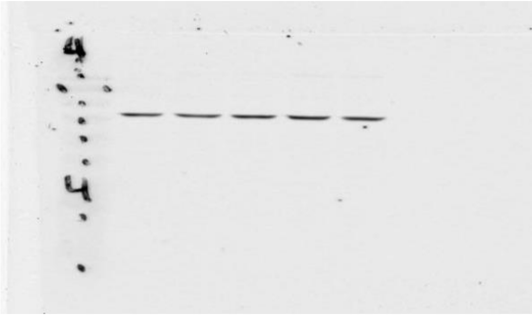

Supplementary Figure 5b

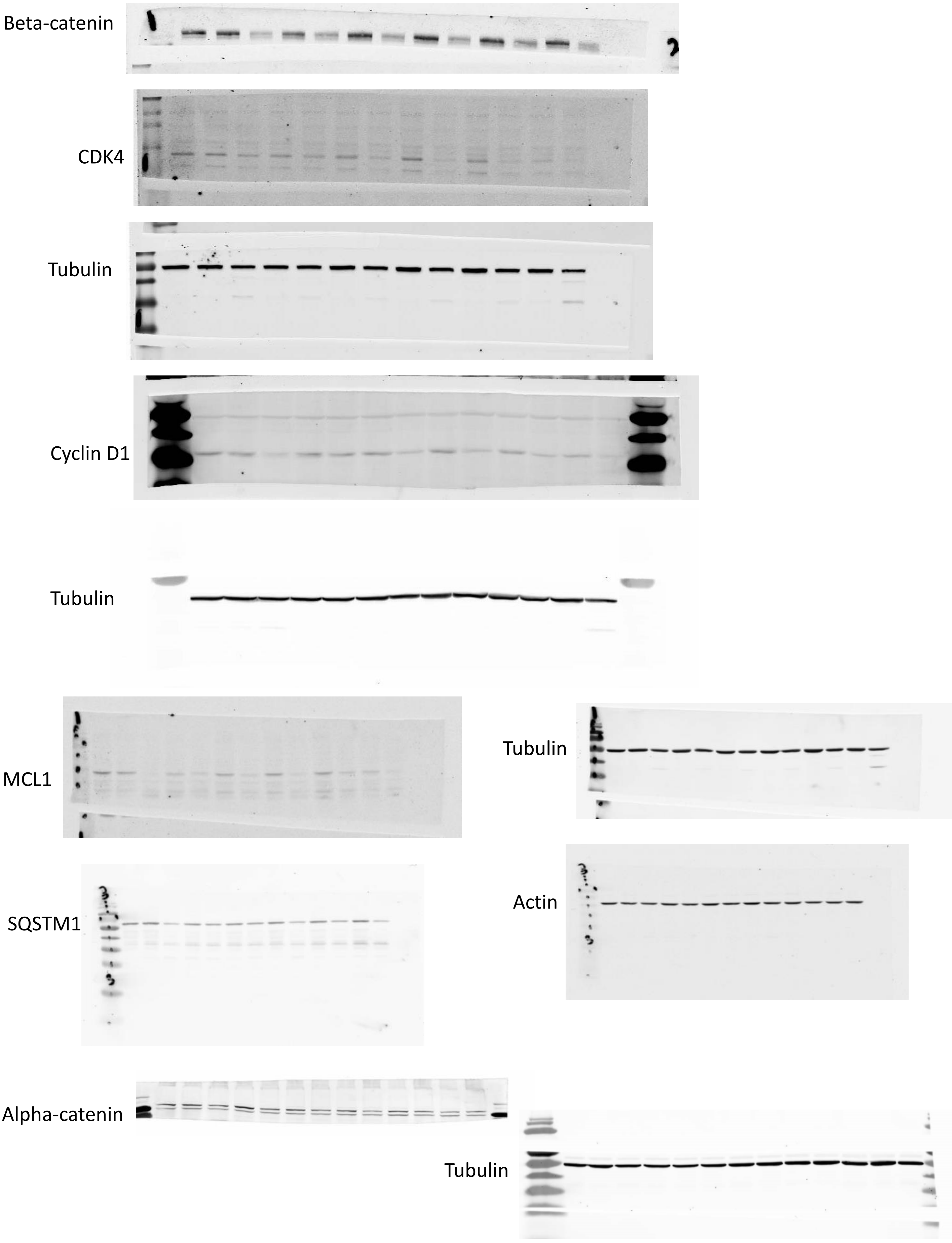

Supplementary Figure 6d

Tubulin →

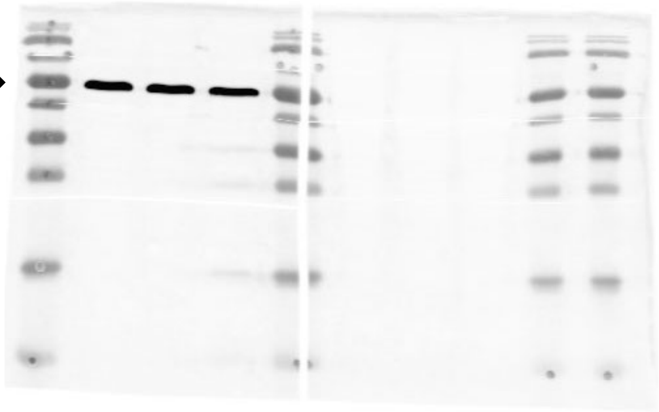

Id1 →

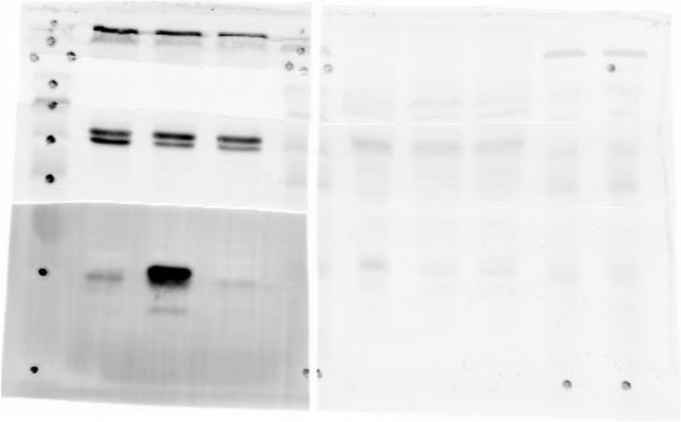

Actin →

Id3 →

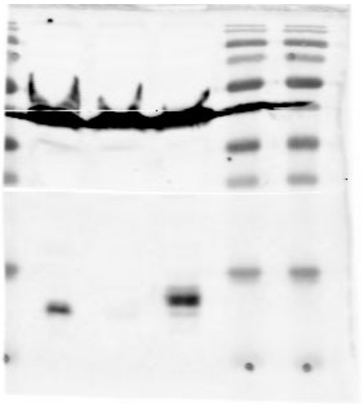

Supplementary Figure 6d

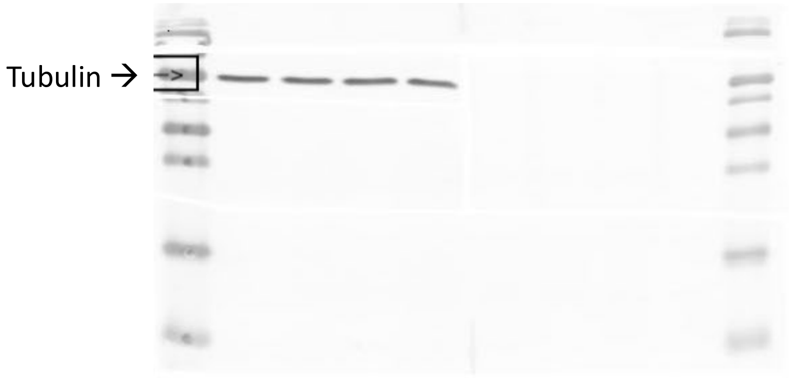

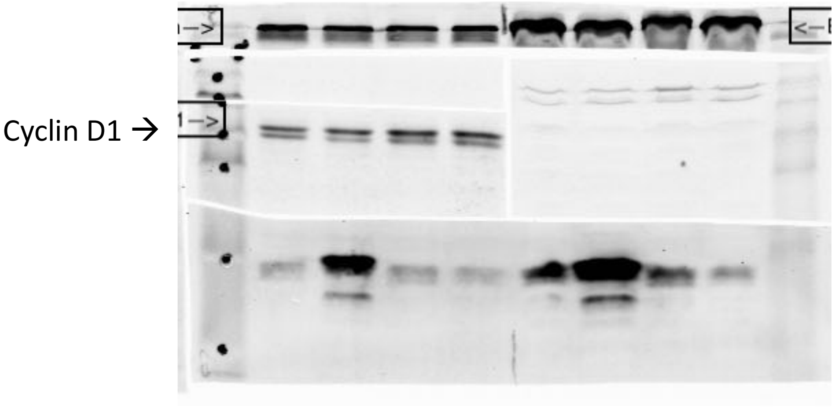

Supplementary Figure 10a

Vimentin

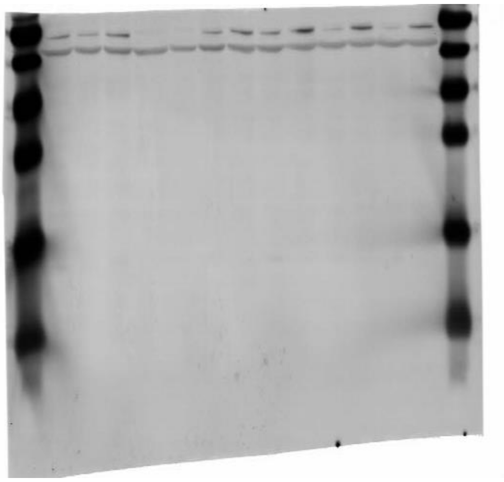

Tubulin

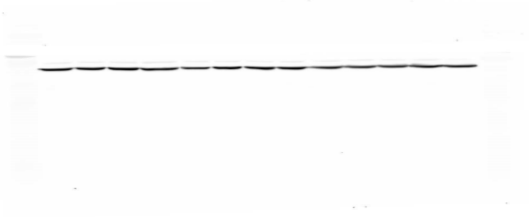

Snail

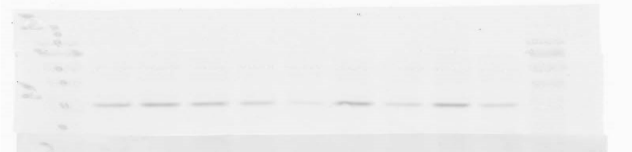

Snail

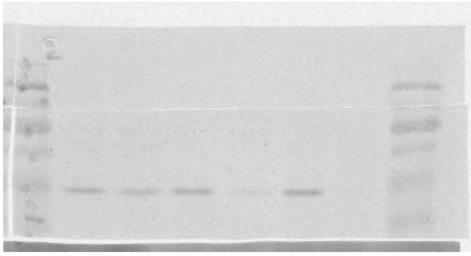

Tubulin

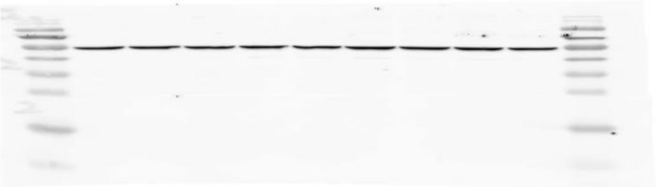

Tubulin

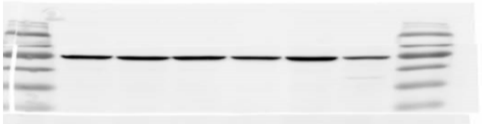

Twist1

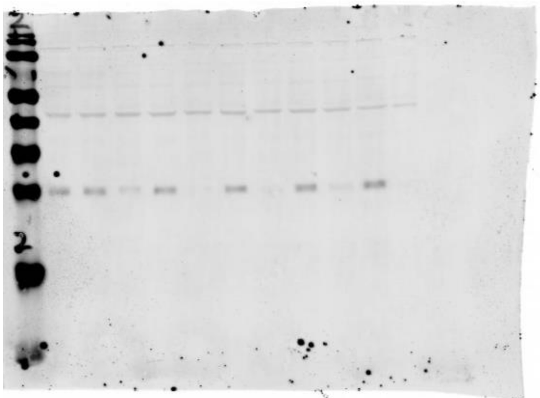

Actin

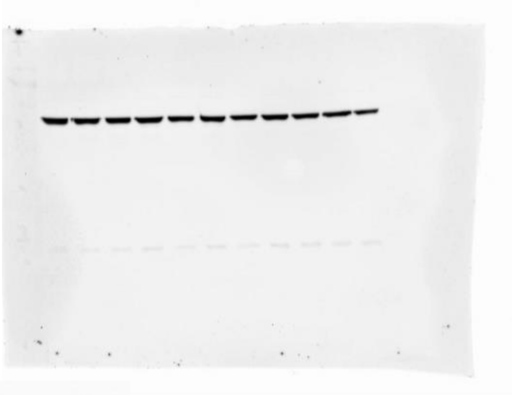

Zeb1

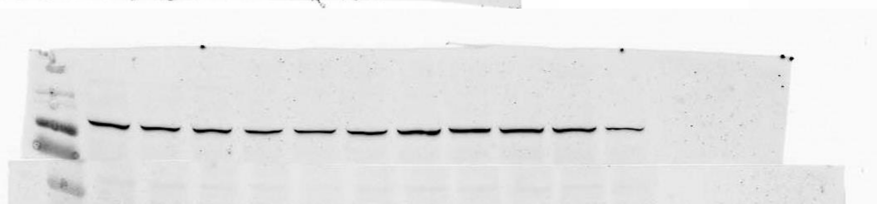

Tubulin

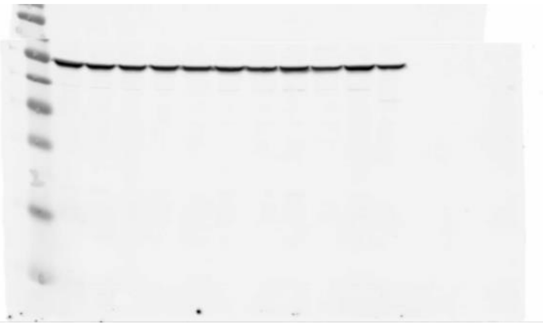

Supplementary Figure 11a

ID1

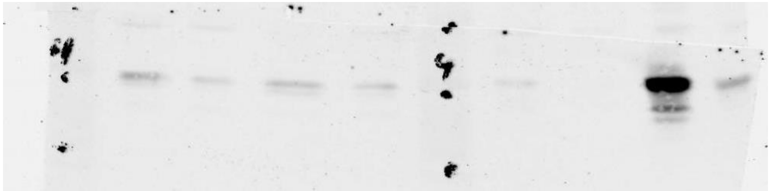

Tubulin

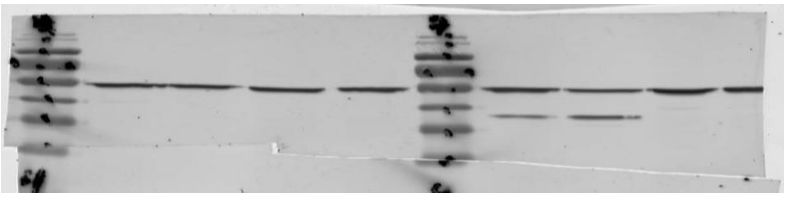

Supplementary Figure 11b

Id1

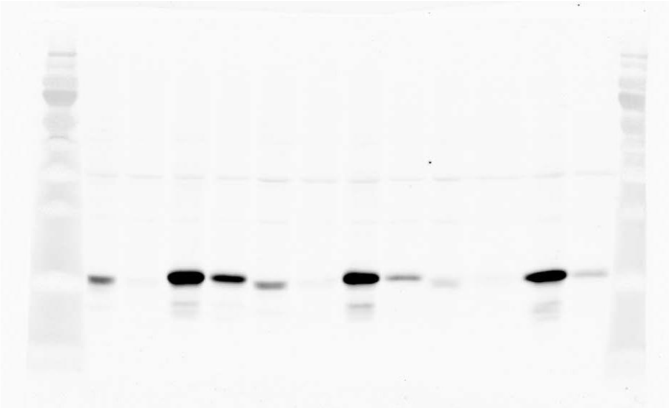

Tubulin

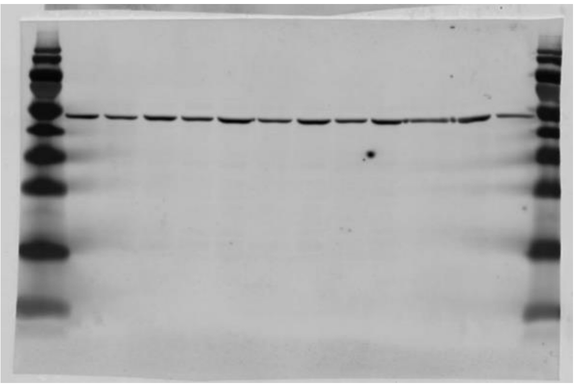

Supplementary Figure 11c

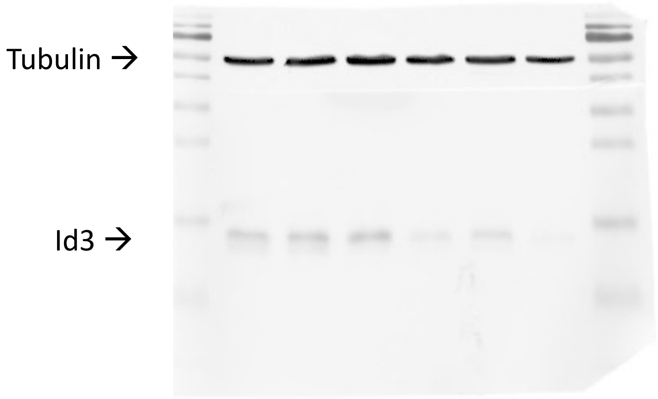

Supplementary Figure 12a

Id1

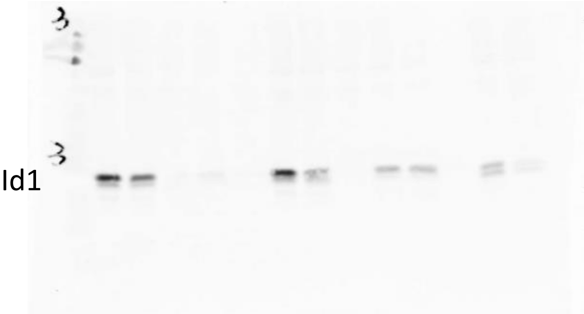

Id1

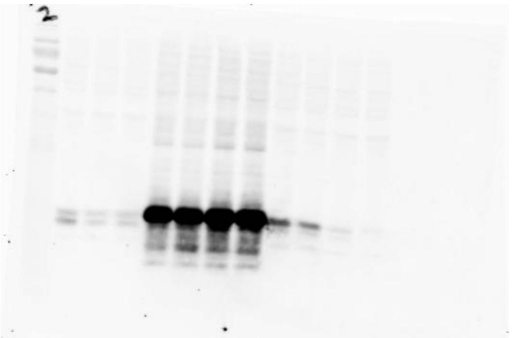

Id1

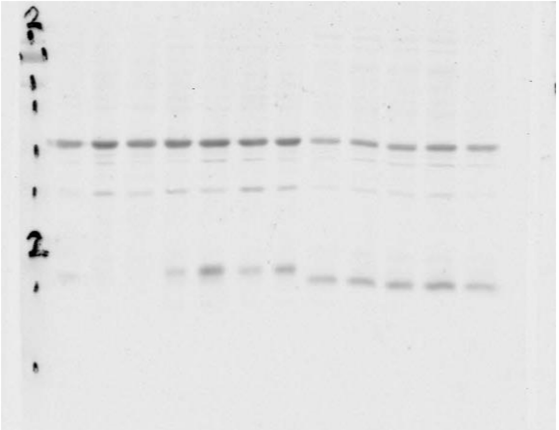

Tubulin

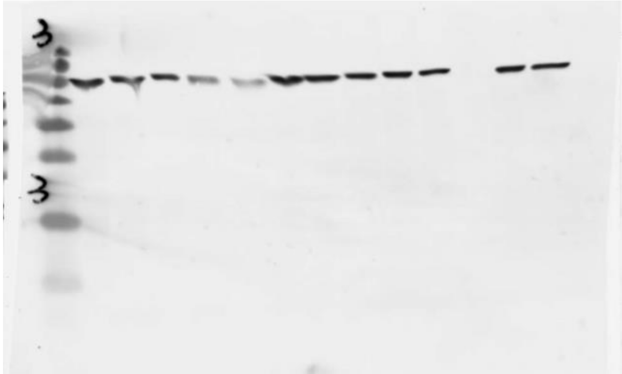

Tubulin

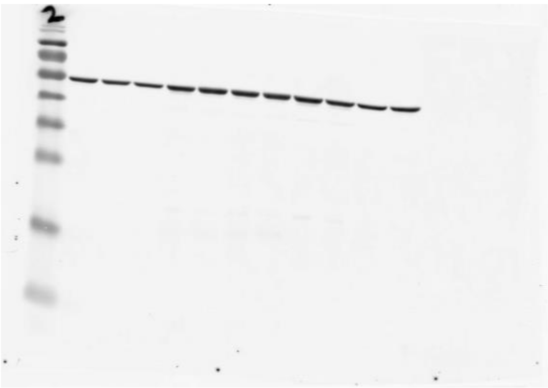

Tubulin

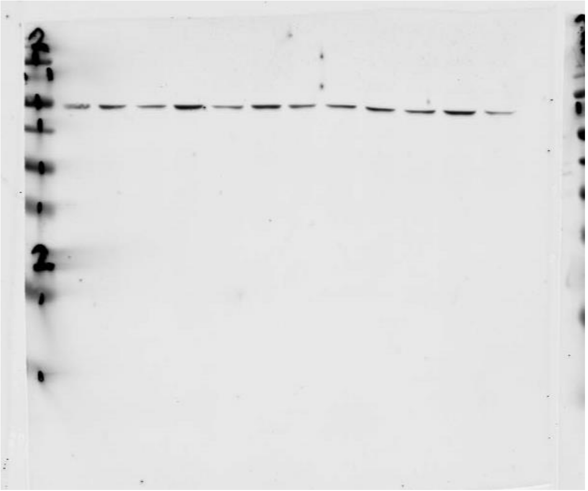

Supplementary Figure 12b

Id3

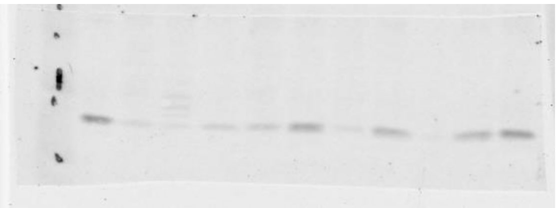

Tubulin

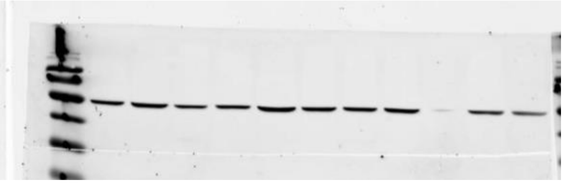

Id4

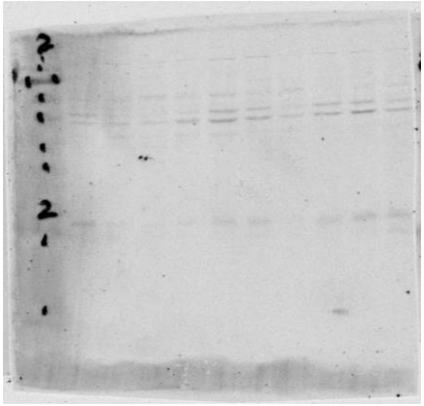

Tubulin

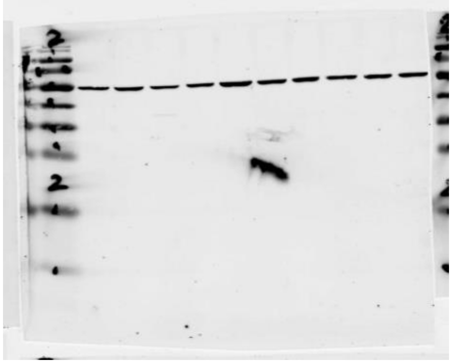

Supplementary Figure 12c

Beta-catenin

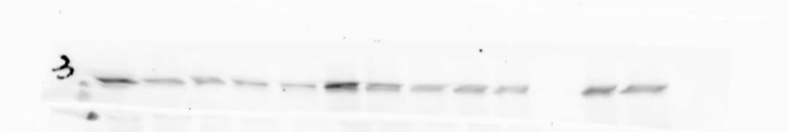

Beta-catenin

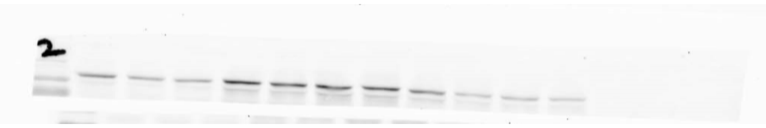

Cyclin D1

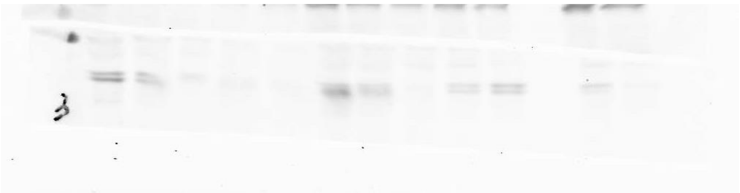

Cyclin D1

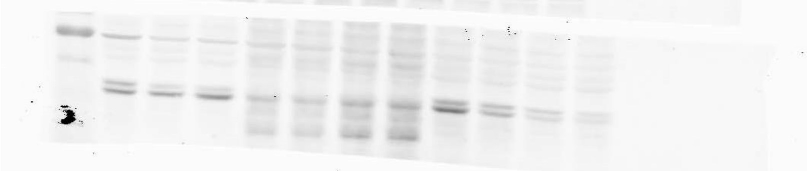

Tubulin

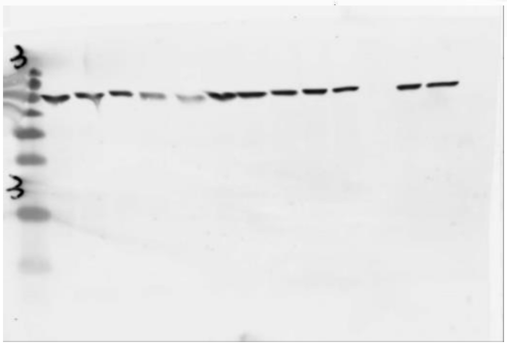

Tubulin

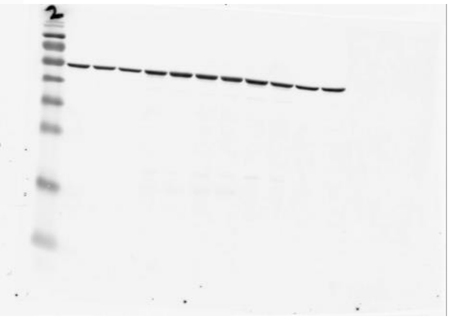

Figure 1f – EMSA

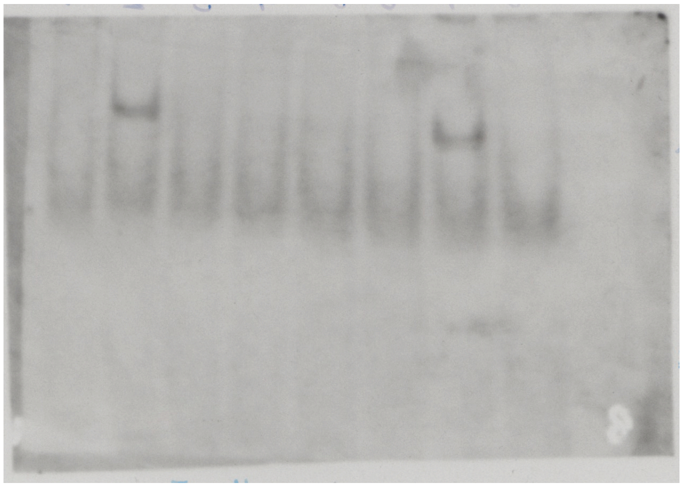

Figure 1g – EMSA

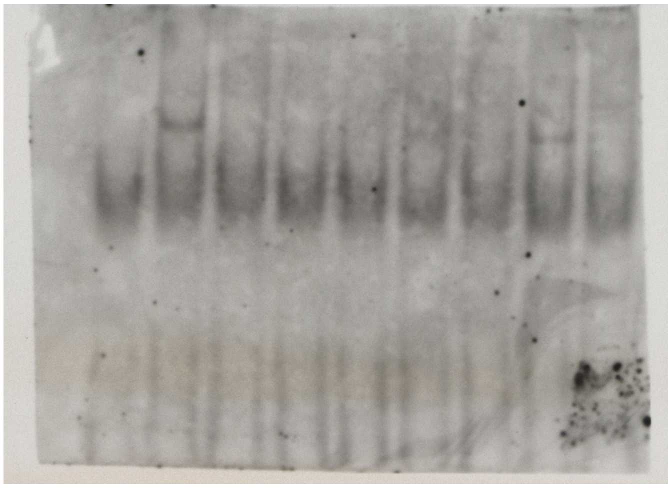

Figure 1g – Western blot

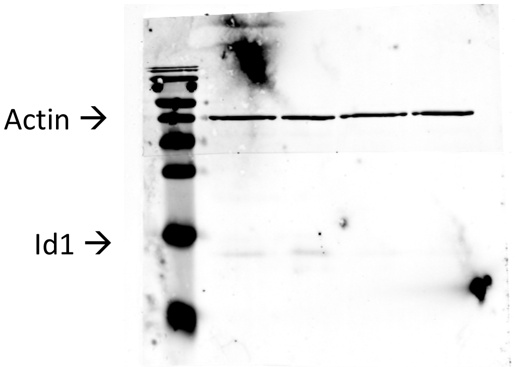

Supplementary Figure 1e – EMSA (HMLE Ras Twist)

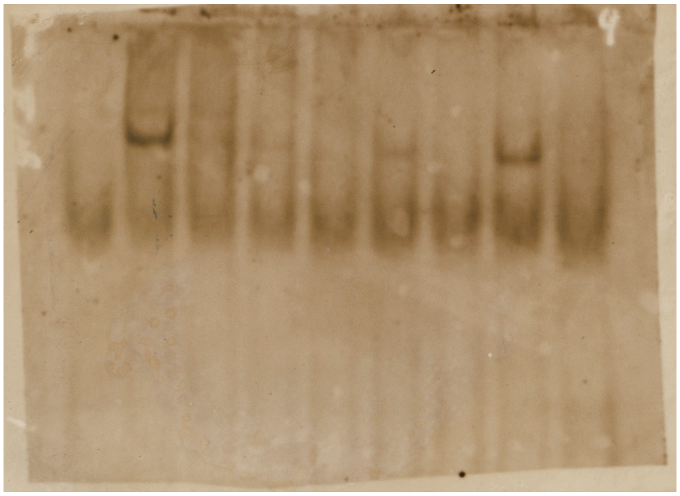

Supplementary Figure 1e – EMSA (PDX IBT)

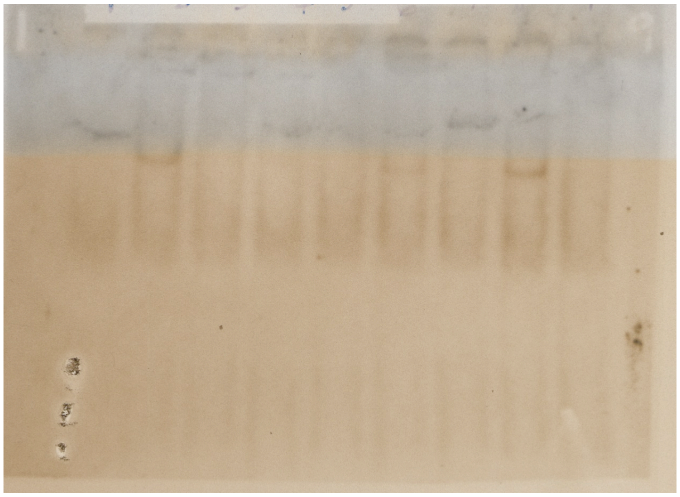

Supplementary Table 1. IC50 values, as determined by MTT assay, for AGX51-treated cell lines. Related to Figure 1.

Cell Type	Cell line	IC50 value after 24 hours of treatment
cell line	4T1	26.66 μM
cell line	HMLE *RAS Twist*	8.7 μM
TNBC cell line	MDA-MB-157	22.28 μM
	MDA-MB-436	30.91 μM
	MDA-MB-231	NA (60 μM=70%)
HER-2 +ve cell line	MDA-MB-453	NA (60 μM=57%)
	BT-474	NA (60 μM=53%)
	MDA-MB-361	NA (60 μM=53%)
	SK-BR-3	36.55 μM
ER +ve cell line	MCF-7	60 μM
	T47-D	NA (60 μM=65%)
PDX cell line	PDX-BR7	10.89 μM
	PDX-IBT	11.97 μM
	PDX-BR11	18.56 μM
cell line	4T1 i.shRNA-*Id1* (-dox.)	25.40 μM
cell line	4T1 i.shRNA-*Id1* (+dox.)	9.03 μM
cell line	4T1 i.shRNA-*Id3* (-dox.)	24.98 μM
cell line	4T1 i.shRNA-*Id3* (+dox.)	8.55 μM

Legend: +, positive; TNBD, Triple negative breast cancer; ER, estrogen receptor; PDX, patient derived xenograft; i.shRNA, inducible shRNA; dox., doxycyclin

Supplementary Table 2. Proteins differently expressed by SILAC in 4T1 cells treated with AGX51 for 4 hours.
Related to Figure 3.

			Fold difference		
**Gene**	**Protein**	**olecular Weig**	**DMSO (Heavy) vs. AGX51**	**DMOS (Light1) vs. AGX (Heavy1)**	**DMOS (Light2) vs. AGX (Heavy2)**
Zbed5	B2RPU8	84 kDa	-2.78	-2.85	-2.64
Sqstm1	SQSTM	44 kDa	-2.36	-2.43	-3.17
Ctnnb1	CTNB1	85 kDa	-2.27	-1.35	-1.41
Morf4l2	MO4L2	32 kDa	-2.24	-1.57	-1.55
Ccnd1	CCND1	33 kDa	-2.17	-1.62	-3.1
Mcl1	MCL1	35 kDa	-2.14	-1.77	-2.01
Ctnna1	CTNA1	100 kDa	-1.63	-2.03	-1.94
Xirp2	XIRP2	428 kDa	-1.63	-1.97	-2.3
Paf	PAF15	12 kDa	-1.51	-1.79	-1.71
Asf1b	ASF1B	22 kDa	-1.5	-1.39	-1.55
Cdk4	CDK4	34 kDa	-1.48	-1.35	-1.44
Uck2	UCK2	29 kDa	-1.44	-1.35	-1.63
Id1	A2AHY3	18 kDa	-1.39	-2.07	-1.71
Ube2c	UBE2C	20 kDa	-1.39	-1.5	-1.59
Vim	VIME	54 kDa	1.36	3.52	1.36
Lonp1	LONM	106 kDa	1.38	2.24	1.96
Phb	PHB	30 kDa	1.38	4.21	1.97
Ptrh2	PTH2	20 kDa	1.38	3.17	1.56
Pam16	TIM16	14 kDa	1.39	2.8	1.52
Sucla2	SUCB1	50 kDa	1.39	2.01	1.48
Cox4i1	COX41	20 kDa	1.44	3.02	1.67
Gnl3	GNL3	61 kDa	1.44	1.5	1.35
Acaa2	THIM	42 kDa	1.5	2.02	1.67
Hadha	ECHA	83 kDa	1.5	2.39	1.68
Ak4	KAD4	25 kDa	1.51	1.46	1.35
Vdac1	VDAC1	31 kDa	1.53	4.29	1.67
Acot13	ACO13	15 kDa	1.54	2.25	1.62
Mrps6	RT06	14 kDa	1.55	1.89	1.41
Fxn	FRDA	23 kDa	1.55	1.69	1.47
Aco2	ACON	85 kDa	1.56	1.98	1.59
Pdhb	ODPB	39 kDa	1.58	1.64	1.6
Hspa9	GRP75	73 kDa	1.58	2.12	1.91
Lrpprc	LPPRC	157 kDa	1.59	2.39	1.5
Idh3a	IDH3A	40 kDa	1.61	2.06	1.63
Gcat	KBL	45 kDa	1.62	2.1	1.49
Pck2	PCKGM	71 kDa	1.62	2.14	1.67
Slirp	SLIRP	11 kDa	1.62	2.68	1.66
Mdh2	MDHM	36 kDa	1.63	2.12	1.78
Got2	AATM	47 kDa	1.63	2.1	1.66
Grpel1	GRPE1	24 kDa	1.63	1.96	1.48
Acat1	THIL	45 kDa	1.63	1.98	1.48
Sod2	SODM	25 kDa	1.66	2.11	1.62
Eci1	ECI1	32 kDa	1.67	2.36	1.54
Cs	CISY	52 kDa	1.67	2.02	1.45
Sdha	SDHA	73 kDa	1.68	2.07	1.52
Idh2	IDHP	51 kDa	1.68	2.22	1.56
Aldh2	ALDH2	57 kDa	1.69	2.49	1.73
Acadl	ACADL	48 kDa	1.71	2.7	1.83
Hadhb	ECHB	51 kDa	1.72	2.73	1.49
Shmt2	Q9CZN7	56 kDa	1.74	2.3	1.66
Trap1	TRAP1	80 kDa	1.74	1.91	1.42
Mthfd1l	C1TM	106 kDa	1.75	2.21	1.65
Hspd1	CH60	61 kDa	1.75	2.42	1.98
Aldh18a1	P5CS	87 kDa	1.75	1.98	2.17
Atp5d	ATPD	18 kDa	1.75	2.8	1.78
Mrps34	RT34	26 kDa	1.75	2.67	1.78
Idh3b	Q91VA7	42 kDa	1.76	2.73	1.64
Atp5b	ATPB	56 kDa	1.78	2.95	1.97
Idh3g	IDHG1	43 kDa	1.78	1.86	1.72
Atp5a1	ATPA	60 kDa	1.79	3.41	1.95
Acot9	ACOT9	51 kDa	1.79	2.2	1.55
Mcoln2	MCLN2	65 kDa	1.79	1.49	2.01
110037F02Ri	E9PZY8	207 kDa	1.8	1.8	1.56
C1qbp	Q8R5L1	31 kDa	1.83	2.47	1.79
Mrps23	RT23	20 kDa	1.83	1.38	1.74
Hsd17b10	Q99N15	27 kDa	1.83	2.3	1.64
Hspe1	CH10	11 kDa	1.84	2.14	1.78
Dld	DLDH	54 kDa	1.85	2.26	1.62
Me2	MAOM	66 kDa	1.85	3.14	1.86
Atp5j2	ATPK	10 kDa	1.92	5.64	2.23
Mrps16	RT16	15 kDa	1.99	2.5	1.78
Mrps17	RT17	13 kDa	2.02	2.33	1.93
Mrpl12	RM12	22 kDa	2.03	2.33	1.69
Mrpl40	D3Z7C0	19 kDa	2.04	2.13	1.69
Prdx3	PRDX3	28 kDa	2.06	2.39	1.89
Dlat	ODP2	68 kDa	2.1	2.55	1.96
Mrpl53	RM53	13 kDa	2.12	2.43	1.81
Dlst	ODO2	49 kDa	2.13	2.01	2.04
Cox5a	COX5A	16 kDa	2.15	2.75	2.02
Glud1	DHE3	61 kDa	2.17	2.01	1.88
Atp5o	ATPO	23 kDa	2.25	4.4	1.96
Atp5h	ATP5H	19 kDa	2.36	5.02	2.45
Nbas	E9Q411	266 kDa	7.01	2.58	1.56
Ifrd1	IFRD1	50 kDa	1536.69	1.95	2.34

Supplementary Table 3. IC50 values, as determined by MTT assay, for AGX51- and AGX8-treated cell lines. Related to Figure 3.

	IC50 value (24 hours)	
**Cell line**	**AGX51**	**AGX8**
4T1	26.66 μM	32.68 μM
HMLE *RAS Twist*	8.7 μM	42.25 μM
4T1 i.shRNA-*Id1* (-dox.)	25.40 μM	39.49 μM
4T1 i.shRNA-*Id1* (+dox.)	9.03 μM	38.77 μM
4T1 i.shRNA-*Id3* (-dox.)	24.98 μM	42.19 μM
4T1 i.shRNA-*Id3* (+dox.)	8.55 μM	41.59 μM

Legend: i.shRNA, inducible shRNA; dox., doxycyclin

Note: AGX51 data is also shown in Extended Data Table 1

Supplementary Table 4. Final tumor volumes of MDA-MB-231 tumors treated with paclitaxel, AGX51, paclitaxel + AGX51 or DMSO. Related to Figure 5.

Treatment group	Tumor volume (mm3)
DMSO	683.91
DMSO	492.82
DMSO	411.77
DMSO	400.60
DMSO	413.37
60mg/kg AGX51 (19 days)	527.73
60mg/kg AGX51 (19 days)	647.84
60mg/kg AGX51 (19 days)	410.90
60mg/kg AGX51 (19 days)	307.18
60mg/kg AGX51 (19 days)	373.24
15mg/kg Paclitaxel+60mg/kg AGX51 (19 days)	161.33
15mg/kg Paclitaxel+60mg/kg AGX51 (19 days)	70.59
15mg/kg Paclitaxel+60mg/kg AGX51 (19 days)	88.88
15mg/kg Paclitaxel+60mg/kg AGX51 (19 days)	50.34
15mg/kg Paclitaxel+60mg/kg AGX51 (19 days)	59.00
15mg/kg Paclitaxel+20mg/kg AGX51 (19 days)	182.17
15mg/kg Paclitaxel+20mg/kg AGX51 (19 days)	295.06
15mg/kg Paclitaxel+20mg/kg AGX51 (19 days)	79.96
15mg/kg Paclitaxel+20mg/kg AGX51 (19 days)	143.02
15mg/kg Paclitaxel+20mg/kg AGX51 (19 days)	42.15
15mg/kg Paclitaxel+6.7mg/kg AGX51 (19 days)	206.13
15mg/kg Paclitaxel+6.7mg/kg AGX51 (19 days)	165.44
15mg/kg Paclitaxel+6.7mg/kg AGX51 (19 days)	184.99
15mg/kg Paclitaxel+6.7mg/kg AGX51 (19 days)	142.83
15mg/kg Paclitaxel+6.7mg/kg AGX51 (19 days)	25.81
15mg/kg Paclitaxel+60mg/kg AGX51 (7 days)	142.16
15mg/kg Paclitaxel+60mg/kg AGX51 (7 days)	109.29
15mg/kg Paclitaxel+60mg/kg AGX51 (7 days)	96.58
15mg/kg Paclitaxel+60mg/kg AGX51 (7 days)	124.37
15mg/kg Paclitaxel+60mg/kg AGX51 (7 days)	50.97
15mg/kg Paclitaxel	305.21
15mg/kg Paclitaxel	152.97
15mg/kg Paclitaxel	267.26
15mg/kg Paclitaxel	259.79
