# Supplementary material for: Anti-tumor effects of an ID antagonist with no observed acquired resistance
Source: NPJ Breast Cancer. 2021 May 24;7:58. doi: 10.1038/s41523-021-00266-0 (PMC8144414; doi:10.1038/s41523-021-00266-0)
Supplement: Supplementary file 2 — Reporting summary. [file 41523_2021_266_MOESM2_ESM.pdf]

nature research

Corresponding author(s): Robert Benezra

Last updated by author(s): Jan 27, 2020

Reporting Summary

Nature Research wishes to improve the reproducibility of the work that we publish. This form provides structure for consistency and transparency in reporting. For further information on Nature Research policies, see our Editorial Policies and the Editorial Policy Checklist.

Statistics

For all statistical analyses, confirm that the following items are present in the figure legend, table legend, main text, or Methods section.

n/a	Confirmed	
☐	☒	The exact sample size (*n*) for each experimental group/condition, given as a discrete number and unit of measurement
☒	☐	A statement on whether measurements were taken from distinct samples or whether the same sample was measured repeatedly
☐	☒	The statistical test(s) used AND whether they are one- or two-sided<br>*Only common tests should be described solely by name; describe more complex techniques in the Methods section.*
☒	☐	A description of all covariates tested
☐	☒	A description of any assumptions or corrections, such as tests of normality and adjustment for multiple comparisons
☐	☒	A full description of the statistical parameters including central tendency (e.g. means) or other basic estimates (e.g. regression coefficient) AND variation (e.g. standard deviation) or associated estimates of uncertainty (e.g. confidence intervals)
☐	☒	For null hypothesis testing, the test statistic (e.g. *F*, *t*, *r*) with confidence intervals, effect sizes, degrees of freedom and *P* value noted<br>*Give P values as exact values whenever suitable.*
☒	☐	For Bayesian analysis, information on the choice of priors and Markov chain Monte Carlo settings
☒	☐	For hierarchical and complex designs, identification of the appropriate level for tests and full reporting of outcomes
☐	☒	Estimates of effect sizes (e.g. Cohen's *d*, Pearson's *r*), indicating how they were calculated

Our web collection on statistics for biologists contains articles on many of the points above.

Software and code

Policy information about availability of computer code

Data collection	Western blot quantification was carried out using channel 700 and channel 800 intensity data from Odyssey application software version 3.0.30 (LI-COR). Whole proteome data was collected and analyzed using Scaffold Q+ version 4.4.5 (Proteome Software, Portland, Oregon, USA). Flow data was collected using Facsdiva v. 8.0.2.
Data analysis	No non-commercially available custom code was used in the analyses. Whole proteome data was collected and analyzed using Scaffold Q+ version 4.4.5 (Proteome Software, Portland, Oregon, USA). Data was analyzed by GraphPad Prism, version 8.1.0 and Microsoft Excel, version 15.4.0.

For manuscripts utilizing custom algorithms or software that are central to the research but not yet described in published literature, software must be made available to editors and reviewers. We strongly encourage code deposition in a community repository (e.g. GitHub). See the Nature Research guidelines for submitting code & software for further information.

Data

Policy information about availability of data

All manuscripts must include a data availability statement. This statement should provide the following information, where applicable:

- Accession codes, unique identifiers, or web links for publicly available datasets
- A list of figures that have associated raw data
- A description of any restrictions on data availability

Raw data are available for the following figures: 1E, 2A, 3B,C,D, 4A,E, 5A,B,F,G 6B, S1A,D,F,G, S2E,I,J S5A,B S6, S7A, S8C. There are no restrictions on data availability

Field-specific reporting

Please select the one below that is the best fit for your research. If you are not sure, read the appropriate sections before making your selection.

☒ Life sciences ☐ Behavioural & social sciences ☐ Ecological, evolutionary & environmental sciences

For a reference copy of the document with all sections, see nature.com/documents/nr-reporting-summary-flat.pdf

Life sciences study design

All studies must disclose on these points even when the disclosure is negative.

Sample size	Three replicates were generally used for each experimental condition for in vitro experiments and 5 mice per group were typically used in each mouse experiment. The sample sizes were determined based on an expected large effect size. With 3 replicated per condition, an effect size as small as 3 can be detected with 80% power at a two-sided significance level of 0.05 using a two- sample t-test. With 5 mice per group, an effect size as small as 2 can be detected with 80% power at a two-sided significance level of 0.05 using a two- sample t-test.
Data exclusions	No data were excluded from the analyses
Replication	All replication attempts were successful.
Randomization	Randomization was not relevant in this study.
Blinding	Blinding was not possible in administration of the test compound AGX51, due to the preparation and appearance of the test compound.

Reporting for specific materials, systems and methods

We require information from authors about some types of materials, experimental systems and methods used in many studies. Here, indicate whether each material, system or method listed is relevant to your study. If you are not sure if a list item applies to your research, read the appropriate section before selecting a response.

Materials & experimental systems

n/a	Involved in the study	
☐	☒	Antibodies
☐	☒	Eukaryotic cell lines
☒	☐	Palaeontology and archaeology
☐	☒	Animals and other organisms
☒	☐	Human research participants
☒	☐	Clinical data
☒	☐	Dual use research of concern

Methods

n/a	Involved in the study	
☒	☐	ChIP-seq
☐	☒	Flow cytometry
☒	☐	MRI-based neuroimaging

Antibodies

Antibodies used	The following primary antibodies were used Id1, Id2, Id3, Id4 (195-14, 9-2-8, 17-3, 82-12, respectively, all from Biocheck), phospho Histone H3 (9701, Cell Signaling), Cyclin D1 (2978, Cell Signaling), Beta-catenin (9562, Cell Signaling), Cdk4 (sc-260, Santa Cruz), Mcl1 (5453, Cell Signaling), Ube2C (14234, Cell Signaling), Sqstm1 (H00008878-M01, Abnova), Alpha-catenin (C2081, SIGMA-ALDRICH), E-cadherin (3195 Cell Signaling), Vimentin (5741, Cell Signaling), Snail (3879, Cell Signaling), Twist (ab50887,Abcam), Zeb1 (NBP1-05987, Novus Biologicals), Actin (A2066, SIGMA), Tubulin (T4026, SIGMA).
Validation	Antibodies were used as described by the manufacturer in terms of species reactivity and applications of use.

Eukaryotic cell lines

Policy information about cell lines

Cell line source(s)	The 4T1 murine mammary tumor cell line, MDA-MB-157, MDA-MB-436, MDA- MB-231, MDA-MB-453, MDA-MB-361, BT-474, SK-BR-3, MCF-7, T47-D and HCT116 were purchased from ATCC (Manassas, VA, USA). Luciferase labeled 4T1 cells were described previously (Granot et al.), as were luciferase and GFP-labeled 4T1 cells (Zimel et al., 2017). 4T1 cells overexpression Id1 were derived by transducing cell with pBabe-Id1 plasmids as described previously (Stankic et al., 2013). HMLE RAS Twist and HMLE RAS Twist ID1 cells were described previously (Stankic et al., 2013).
Authentication	4T1 cells were authenticated by short tandem repeat analysis and karyotyping. Other cell lines have not been authenticated since being obtained from their source.

Mycoplasma contamination	The cell lines were not tested for mycoplasma contamination recently but routine testing had been negative for years prior and no growth defects associated with such contamination have been observed in any of the cell lines used in the studies presented.
Commonly misidentified lines (See ICLAC register)	No misidentified lines were used in this study.

Animals and other organisms

Policy information about studies involving animals; ARRIVE guidelines recommended for reporting animal research

Laboratory animals	Weight measurements and standard blood analyses were carried out on 8-12 week-old male CD1 mice. Orthotopic mammary fat pad tumors were generated in 8-12 week-old, female athymic nu/nu mice (Simonsen Laboratories). Lung metastases were generated by injecting 6-8 week-old, female, Balb/c mice (Taconic). Spontaneous colon tumors were induced by treating 30, 4-week old male A/J mice (Jackson Laboratory).
Wild animals	The study did not involve wild animals
Field-collected samples	The study did not involve samples collected from the field
Ethics oversight	Animal studies were carried out in accordance with institutional regulations (IACUC protocol 06-10-025).

Note that full information on the approval of the study protocol must also be provided in the manuscript.

Flow Cytometry

Plots

Confirm that:

- [x] The axis labels state the marker and fluorochrome used (e.g. CD4-FITC).
- [x] The axis scales are clearly visible. Include numbers along axes only for bottom left plot of group (a 'group' is an analysis of identical markers).
- [ ] All plots are contour plots with outliers or pseudocolor plots.
- [x] A numerical value for number of cells or percentage (with statistics) is provided.

Methodology

Sample preparation	The 4T1 mammary carcinoma was originally derived from the mouse mammary gland.<br>4T1 cells permanently carrying pWPI-hId3 (GFP+) or pWPI (GFP+) cassette were seeded in a 10-cm dish overnight, and were harvested by treating with 0.25% (w/v) trypsin, 2 mM EDTA without Ca++ and Mg++ solution. About 1x107 cells were collected and pelleted at 1200 rpm for 5 min. Cells were re-suspended in 500 ul in a PBS without Ca++ and Mg++ with 1% FBS. Re-suspended cells were filtered through Blue Flow Cytometry Cap for FACS Tubes with 35µm Strainer Mesh (Stellar Scientific). 4T1 cells (GFP-negative) were used as negative control for FACS. After FACS cells were collected in tubes treated with 15% FBS in PBS.
Instrument	FACS Aria II (Aria3)
Software	Facsdiva v. 8.0.2. FCS express.
Cell population abundance	Cell population abundance after sorting 4T1 cells transduced with the lentiviral construct pWPI was 3.7-3.8% with 30,700 - 34,708 GFP-positive cells. Cell population abundance after sorting 4T1 cells transduced with the lentiviral construct pWPI-hId3 was 6% with 83,926 GFP-positive cells, and 4T1 pWPI-hId1 cells was 0.7% with 44,564 - 47,599 GFP-positive cells. The purity of the sample was determined based on the GFP-fluorescence.
Gating strategy	Gating strategy was based on a hierarchy of the gates. At first, a scatter gate (P1) was selected, where SSC-A vs FSC-A (y, x axis), were plotted to exclude cellular debris. Then double doublet discrimination gates (P2 and P3) were selected, where FSC-A vs FSC-H and SSC-W vs SSC-H (y, x axis), were plotted. Finally, a GFP+ gate (P5) was selected where PE-A vs GFP-A. The boundaries between GFP-positive and negative cells was based on the intensity of the GFP fluorescence.

- [ ] Tick this box to confirm that a figure exemplifying the gating strategy is provided in the Supplementary Information.
